# Incorporating Non-Coding Annotations into Rare Variant Analysis

**DOI:** 10.1371/journal.pone.0154181

**Published:** 2016-04-29

**Authors:** Tom G. Richardson, Colin Campbell, Nicholas J Timpson, Tom R. Gaunt

**Affiliations:** 1 MRC Integrative Epidemiology Unit, School of Social and Community Medicine, University of Bristol, Bristol, United Kingdom; 2 Intelligent Systems Laboratory, University of Bristol, Bristol, United Kingdom; The University of Hong Kong, HONG KONG

## Abstract

**Background:**

The success of collapsing methods which investigate the combined effect of rare variants on complex traits has so far been limited. The manner in which variants within a gene are selected prior to analysis has a crucial impact on this success, which has resulted in analyses conventionally filtering variants according to their consequence. This study investigates whether an alternative approach to filtering, using annotations from recently developed bioinformatics tools, can aid these types of analyses in comparison to conventional approaches.

**Methods & Results:**

We conducted a candidate gene analysis using the UK10K sequence and lipids data, filtering according to functional annotations using the resource CADD (Combined Annotation-Dependent Depletion) and contrasting results with ‘nonsynonymous’ and ‘loss of function’ consequence analyses. Using CADD allowed the inclusion of potentially deleterious intronic variants, which was not possible when filtering by consequence. Overall, different filtering approaches provided similar evidence of association, although filtering according to CADD identified evidence of association between *ANGPTL4* and High Density Lipoproteins (P = 0.02, N = 3,210) which was not observed in the other analyses. We also undertook genome-wide analyses to determine how filtering in this manner compared to conventional approaches for gene regions. Results suggested that filtering by annotations according to CADD, as well as other tools known as FATHMM-MKL and DANN, identified association signals not detected when filtering by variant consequence and vice versa.

**Conclusion:**

Incorporating variant annotations from non-coding bioinformatics tools should prove to be a valuable asset for rare variant analyses in the future. Filtering by variant consequence is only possible in coding regions of the genome, whereas utilising non-coding bioinformatics annotations provides an opportunity to discover unknown causal variants in non-coding regions as well. This should allow studies to uncover a greater number of causal variants for complex traits and help elucidate their functional role in disease.

## Introduction

Genome wide association studies (GWAS) have had a profound influence on the number of disease associated common variants detected across the genome, although recently a greater emphasis has been placed on the impact that rarer variants can have on disease[1, 2]. The advent of next generation sequencing (NGS) has facilitated the development of rare variant association approaches by collapsing variants within the same gene together and analysing their combined effect on phenotypic traits[3]. The aim of current endeavors is to identify deleterious regions of variants with potentially larger effect sizes than those typically identified in GWAS[4], subsequently improving the explained heritability of common diseases. Furthermore, rare variant analyses can help identify genes that are relevant to a particular disease, where evidence gained from single variant approaches may be limited.

Rare variant association studies have had varying degrees of success in recent years[5, 6]. The number of variants with little to no effect (neutral variants) and also variants with contrasting directions of effect within a collapsed region often weaken the statistical power of analyses. As a result, methods based around the analysis of variance-components (e.g. C-Alpha[7], Sequence Kernel Association Test (SKAT)[8]) have been developed to overcome these challenges. Furthermore, filtering variants by their functional consequence (e.g. only analysing variants within a region that are predicted to have a ‘nonsynonymous’ or ‘loss of function’ impact) has become common practice to reduce the potential number of neutral variants within a collapsed region or functional unit.

Filtering in this manner can have limitations for several reasons. Firstly, even if a variant is annotated correctly, this does not necessarily mean that it will have a deleterious effect (e.g. if a nonsynonymous variant is located within the transmembrane region of a receptor gene it may not drastically alter the function of the protein). Secondly, it is also possible that other types of variants within the coding region of the gene (i.e. silent mutations) can potentially have a deleterious effect but may get filtered out. Lastly, filtering variants according to their consequence is limited to coding regions, therefore making annotations for filtering non-coding regions (i.e. intronic regions of a gene or intergenic regions of the genome) an attractive commodity, particularly with the current influx of whole genome sequence (WGS) data.

The Combined Annotation-Dependent Depletion (CADD)[9] method objectively integrates a range of different annotation metrics into a single measure (C score). By doing so, CADD aims to provide a more reliable estimate of deleteriousness for all known variants and therefore an overall rank for this metric across the genome. Other bioinformatics tools, such as SIFT [10] and Polyphen-2 [11] have previously been used to filter variants for rare variant analyses [12], although importantly these tools use protein-based metrics and are therefore confined to coding regions. Although CADD has been used to further evaluate the impact of SNPs after identification in association studies, this resource has not yet been utilised to filter variants according to prior knowledge about likely function before undertaking a low frequency or rare variant association analysis. We therefore hypothesised that, in comparison to filtering by variant consequence, using CADD may be more informative in identifying functional variants for a complex trait, whilst keeping the number of neutral variants to a minimum. Moreover, when applying collapsing methods to gene regions, CADD allows analyses to be undertaken in intronic regions of the gene which can potentially harbour functional variants [13], which would not be possible when filtering by variant consequence or prediction tools confined to coding regions.

Using WGS data from the UK10K project (http://www.uk10k.org), we have undertaken low frequency and rare variant analyses to evaluate whether filtering variants according to CADD scaled C-Scores identifies association signals not detected when filtering according to variant consequence. Our hypothesis was that incorporating non-coding bioinformatics annotations based on predicted variant functionality would be a valuable asset for future studies which conduct these types of approaches.

## Results

3,781 whole-genome samples from the UK10K cohort arm [14] were available for analysis (1,927 from ALSPAC, 1,854 from TwinsUK) after variant calling and quality control. After merging individuals with each cardiovascular, final sample sizes ranged between 3,538 and 3,191 (3,538 for Body Mass Index (BMI), 3,309 for Systolic and Diastolic Blood Pressure (SBP & DBP), 3,210 for High Density Lipoproteins (HDL), 3,191 for Low Density Lipoproteins (LDL), 3,206 for Total Cholesterol (TC) and 3,202 for Triglycerides (TG)).

### Candidate Gene Analysis

We used CADD[9] to obtain scaled C scores for all 44.9 million possible variants and indels in the UK10K whole genome sequence data. After removing variants which failed QC, we filtered all variants in three ways:

1. Variants responsible for a ‘nonsynonymous’ substitution according to the Variant Effect Predictor [15] (VEP). VCFtools [16] was subsequently used to condense these regions down to just those variants.
2. Variants responsible for a ‘loss of function’ according to VEP (i.e. ‘stop losses/gains’, ‘splice sites’ or ‘frameshift indels’). VCFtools was used again to condense these regions down to just those variants.
3. Variants with a CADD C-Score ≥15 (i.e. the 5% most damaging variants predicted across the genome). This is a suggested cutoff by the developers of CADD to identify potentially pathogenic variants as it is the median value for all possible canonical splice site changes and nonsynonymous variants (http://cadd.gs.washington.edu/info).

We collapsed variants together across candidate genes and analysed them using SKAT[8] with their associated traits according to Liu et al[17]. These genes were *ANGPTL4*, *BCAM*, *CBLC*, *CD300LG*, *HNF4A*, *LDLR*, *LIPC*, *LIPG*, *LPL*, *PCSK9* and *PVR*. Analyses were repeated after applying each variant filtering method, as well as applying two different minor allele frequency (MAF) cutoffs of MAF≤5% and MAF≤1%. Importantly, filtering by variant consequence was confined to coding regions, whereas filtering by CADD definitions also allowed the inclusion of potentially deleterious non-coding variants which reside in intronic regions of genes. The results of this analysis, as well as those in subsequent analyses of this study, were not adjusted for multiple comparisons. This was because we were interested in the comparison of filtering approaches in terms of identifying association signals, rather than evaluating whether these signals are real, which is important for analyses regardless of filtering method. Furthermore, the same number of analyses were conducted for each filtering method as this would have otherwise incorporated bias into the study.

Using a MAF cutoff of 5%, we observed evidence of association between *LIPG* and HDL (P = 0.02) as well as between *PVR* and LDL (P = 0.02) after filtering to only include nonsynonymous variants. For the loss of function variant analyses, *CD300LG*, *BCAM* and *ANGPTL4* were associated with HDL, LDL and TG respectively (P = 0.02, P = 0.04 and P = 0.03). Using CADD to filter variants provided evidence of association between three of the previously mentioned genes and traits, as well as between *ANGPTL* and HDL (P = 0.02). The majority of these effects appeared to be driven by rare variants, as evidence of association was observed after applying a MAF cutoff of 1%. The only exception to this was the association between *LIPG* and HDL after filtering to include nonsynonymous variants, which did not provide strong evidence of association using this cutoff (P = 0.07). Tables 1 and 2 show the complete results of our gene-level low frequency variant and rare association analyses, using a cutoff of 5% and 1% MAF respectively.

**Table 1:** Results of gene-level low frequency variant association tests using various variant filters (MAF ≤ 5%).

		Nonsynonymous variants		Loss-of-Function variants		CADD variants (C-Score ≥ 15)	
Gene	Lipid trait	nVars	P-value	nVars	P-value	nVars	P-value
*LIPC*	HDL	30	0.85	3	0.81	57	0.18
*LPL*	HDL	20	0.54	4	0.13	12	0.81
*ANGPTL4*	HDL	11	0.17	2	0.98	5	0.02
*LIPG*	HDL	**16**	**0.02**	2	0.89	**11**	**0.02**
*HNF4A*	HDL	23	0.15	5	0.28	29	0.80
*CD300LG*	HDL	15	0.11	**3**	**0.02**	4	0.37
*PCSK9*	LDL	21	0.21	3	0.09	11	0.82
*BCAM*	LDL	36	0.28	7	**0.04**	**5**	**0.02**
*CBLC*	LDL	16	0.86	5	0.77	3	0.39
*PVR*	LDL	**12**	**0.02**	2	0.08	8	0.12
*LDLR*	LDL	43	0.93	10	0.94	11	0.29
*ANGPTL4*	TG	11	0.25	**2**	**0.03**	**5**	**0.05**
*LPL*	TG	20	0.83	4	0.51	12	0.56

nVars = number of variants analysed, HDL = High Density Lipoproteins, LDL = Low Density Lipoproteins, TG = Triglycerides, MAF = Minor Allele Frequency. Results in bold represent p-values ≤ 0.05. No multiple testing threshold was applied to the results of this analysis as the purpose was to compare filtering approaches. All p-values were calculated using SKAT.

**Table 2:** Results of gene-level rare variant association tests using various variant filters (MAF ≤ 1%).

		Nonsynonymous variants		Loss-of-Function variants		CADD variants (C-Score ≥ 15)	
Gene	Lipid trait	nVars	P-value	nVars	P-value	nVars	P-value
*LIPC*	HDL	27	0.32	2	0.40	54	0.37
*LPL*	HDL	18	0.12	4	0.13	11	0.54
*ANGPTL4*	HDL	11	0.17	2	0.98	**5**	**0.02**
*LIPG*	HDL	15	0.07	2	0.89	**10**	**0.05**
*HNF4A*	HDL	21	0.32	5	0.28	28	0.60
*CD300LG*	HDL	14	0.10	**3**	**0.02**	3	0.28
*PCSK9*	LDL	19	0. 70	2	0.72	11	0.82
*BCAM*	LDL	32	0.16	7	**0.04**	**3**	**0.02**
*CBLC*	LDL	12	0.57	4	0.43	2	0.26
*PVR*	LDL	**11**	**0.02**	1	N/A	8	0.13
*LDLR*	LDL	41	0.92	9	0.93	11	0.28
*ANGPTL4*	TG	11	0.25	**2**	**0.03**	**5**	**0.05**
*LPL*	TG	18	0.45	4	0.51	11	0.26

nVars = number of variants analysed, HDL = High Density Lipoproteins, LDL = Low Density Lipoproteins, TG = Triglycerides, MAF = Minor Allele Frequency. Results in bold represent p-values ≤ 0.05. No multiple testing threshold was applied to the results of this analysis as the purpose was to compare filtering approaches. All p-values were calculated using SKAT.

### Genome-wide Analysis of Gene-based Association Signals using CADD

We also identified variants with a CADD C-Score ≥15 across the genome and aggregated them together across all gene regions according to UCSC definitions (reference genome hg19). Variants were then collapsed together and analysed with 7 cardiovascular traits (BMI, SBP, DBP, HDL, LDL, TC & TG) using SKAT after applying a MAF cutoff of 5%. We repeated this process in a second set of analyses using a MAF cutoff of 1% to investigate rarer variation. This process was repeated except filtering to include variants which led to a ‘nonsynonymous’ or ‘loss-of-function’ consequence (i.e. regardless of CADD C-Score) according to dbSNP annotations (build 137) in two other separate sets of analyses. Only regions which had at least 2 variants remaining after filtering were eligible for analyses, as a single remaining variant would offer no added value when analysed using SKAT compared to using a single variant test.

**Fig 1:**
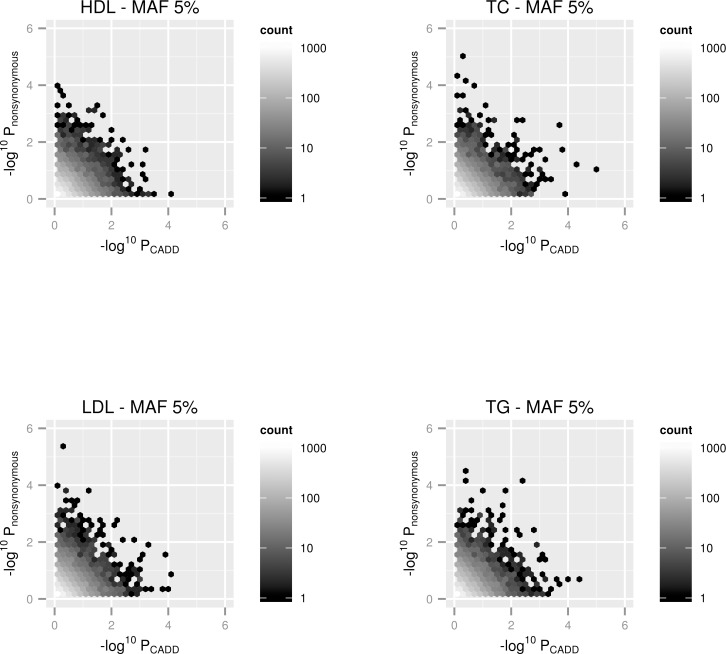
Hexbin plots representing gene-based SKAT analyses for all genes across the genome using a MAF cutoff of 5% with 4 lipid traits. The x-axis represents the–log10 transformed p-value from the analysis after filtering according to CADD annotations. The y-axis represents the–log10 transformed p-value from the analysis after filtering according to ‘nonsynonymous’ annotations. Only gene regions which had at least 2 variants in them after filtering by both methods were plotted.

**Fig 2:**
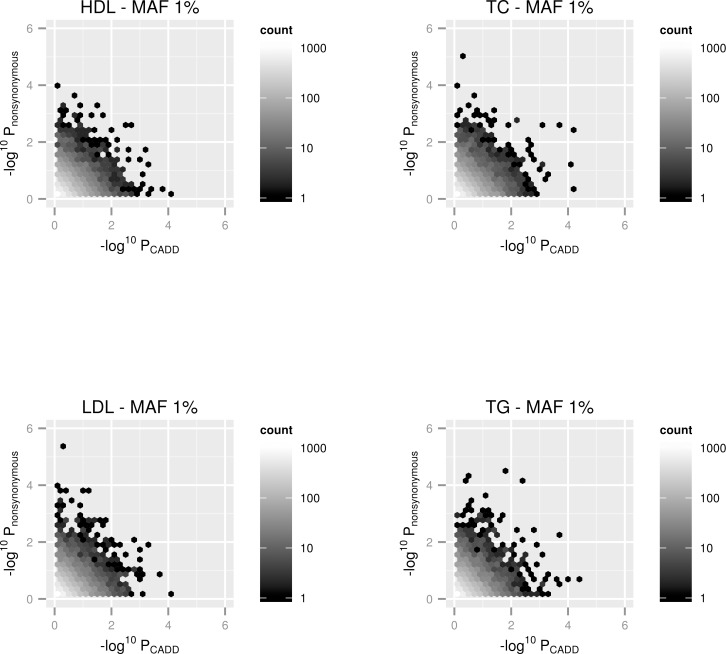
Hexbin plots representing gene-based SKAT analyses for all genes across the genome using a MAF cutoff of 1% with 4 lipid traits. The x-axis represents the–log10 transformed p-value from the analysis after filtering according to CADD annotations. The y-axis represents the–log10 transformed p-value from the analysis after filtering according to ‘nonsynonymous’ annotations. Only gene regions which had at least 2 variants in them after filtering by both methods were plotted.

Gene-based p-values from the CADD filtered analyses were matched with p-values from the ‘nonsynonymous’ filtered analysis and the results were–log10 transformed and plotted for each trait and MAF cutoff. This meant that only genes which had at least 2 variants within their region after filtering in both sets of analyses (i.e. ≥ 2 ‘nonsynonymous’ & ≥ 2 variants with a CADD C-Score ≥15) were plotted on these graphs. Due to the frequency of points on these plots, overall trends would have been very challenging to identify using scatter plots. We therefore used hexbin plots for this task [18], which allows density of the number of points within each region of the plot to be incorporated. Overall there was little evidence that filtering according to CADD annotations provided either stronger or weaker evidence of association across the genome in comparison filtering by nonsynonymous consequence. This can be observed in the hexbin plots as the gradient of colour is consistent through the plots and does not favour either axis. Moreover, some of the lowest gene-based p-values were only observed using one filtering approach for each trait, implying that there was not always strong concordance between the different methods (i.e. evidence of association was only observed when using CADD filtering and vice versa). Figs 1 and 2 show the hexbin plots for the 4 lipid traits investigated in this analysis for low frequency (MAF≤5%) and rare variant (MAF≤1%) analyses respectively. Plots for the other cardiovascular traits can be found in the S1 File.

Gene-based p-values from all CADD filtered analyses were also matched with those observed from the loss-of-function analyses. However, simple scatter plots were sufficient for this data as there were far fewer than in the comparison with nonsynonymous filtering. It is also worth clarifying that these loss-of-function variants may also have been included in the nonsynonymous analysis, as variants can be classed as both (i.e. a variant which alters the amino acid sequence of a protein and also results in a stop codon). As before, there was no overall trend to suggest that filtering using either approach provided stronger evidence of association with cardiovascular traits. Again there were results which represented a lack of concordance between filtering approaches, suggesting that evidence of association with traits would only be observed when filtering with one method but not the other. Scatter plots for these analyses can be found in S1 File. Plots were also generated for the results of identical analyses except using the SKAT-O [19] and MiST [20] collapsing methods, although the overall findings were the same using these approaches. These plots can also be found in S1 File.

### Genome-wide Analysis of Gene-based Association Signals using FATHMM-MKL and DANN

The previous analysis was repeated except using two alternative bioinformatics tools rather than CADD. These were FATHMM-MKL [21] and DANN [22], which use a machine learning and deep neural network approach respectively to assess the functional consequence of variants. The metrics for both tools are bounded between 0 and 1, where a larger score means that variants are predicted to have a more deleterious effect. Applying a threshold of 0.9 for both tools (i.e. only variants predicted to have a strong deleterious effect) left 1,208,786 variants using FATHMM-MKL and 1,331,967 variants using DANN in our dataset.

FATHMM-MKL and DANN analyses were conducted separately. Analyses were undertaken as before, collapsing variants by gene regions and analysing them with each cardiovascular trait in turn after applying a MAF cutoff of 5% and 1%. Gene regions were analysed twice using the SKAT-O and MiST tests and only regions which had at least 2 variants after filtering were eligible for analysis. Hexbin and scatter plots were again used to display results compared to the ‘nonsynonymous’ results and the ‘loss-of-function’ results respectively. The FATHMM-MKL plots suggested similar inferences to the CADD based analyses, whereas certain DANN analyses using SKAT-O (particularly for TG) appeared to have more concordance with results using nonsynonymous annotations. Plots from all these analyses can be found in S1 File.

## Discussion

We have conducted a candidate gene study to evaluate whether incorporating variant annotations using a non-coding bioinformatics tool can aid rare variant analyses over filtering by variant consequence. Filtering according to CADD provided evidence of association between genes and lipids similar to filtering by variant type, as well as moderate association between *ANGPTL4* and HDL (P = 0.02). We have also undertaken extensive genome wide analyses to determine how filtering variants using bioinformatics annotations compares to filtering by variant consequence within gene regions, when conducting low frequency and rare variant collapsing analyses. These results were plotted as hexbin and scatter plots, which suggested the different approaches to filtering variants yield different sets of associated loci. The consequence of this meant that certain association signals were only detected when filtering by annotations from the bioinformatics tools and not according to variant consequence, and vice versa. This suggests that future studies should benefit from this alternative approach to identify association signals potentially overlooked using conventional methods, due to the inclusion of non-coding regions which may harbour potentially deleterious variants.

The reason why this novel approach to filtering variants may identify association signals not detected when filtering by variant consequence could be influenced by several factors. Firstly, this could be due to the inclusion of predicted deleterious synonymous variants which therefore improves the statistical power of analyses. Furthermore, using these annotations may lead to removing predicted neutral nonsynonymous variants (i.e. variants which alter amino acid sequence, but not the function of a protein) which reduces the amount of statistical noise incorporated into analyses. Fig 1B of the CADD manuscript by Kircher et al shows that a threshold of 15 should also be including a proportion of other types of variants (e.g. which are predicted to be the most deleterious within each of their categories, along with the majority of nonsynonymous variants). Filtering using a non-coding algorithm also allows the inclusion of variants which reside in intronic regions of genes, which can also have functional consequences on human disease through altering regulator or splicing sequence [23]. Association signals driven by variants within these regions may therefore be of great importance in terms of disease aetiology, although collapsing approaches which filter by variant consequence are confined to coding regions of the genome and therefore these variants are not investigated. Incorporating non-coding bioinformatics annotations into rare variant analysis should therefore aid future studies in terms of addressing the limitations of conventional approaches.

The ability to adjust the inclusion threshold for variants when using prediction algorithm annotations is also advantageous to studies. In this study, we have used a CADD C-Score of ≥ 15 as our inclusion threshold, which is suggested by the authors of CADD to identify potentially pathogenic variants as it is the median value for all possible canonical splice site changes and nonsynonymous variants [9]. However, the optimal threshold of CADD C-Score to uncover causal variants may depend on several factors, such as the trait analysed. With the particular interest regarding the contribution that low frequency and rare variants can have on blood lipid levels currently [17, 24, 25], we decided to analyse lipid and other cardiovascular traits in this study to evaluate our hypothesis. However, filtering using bioinformatics annotations should also benefit analyses undertaken with other complex traits. Likewise, the length of a gene is another factor which may cause varying cutoffs to result in stronger evidence of association detected from analyses. It may therefore be beneficial to use a stricter threshold (i.e. more confidence that variants within a region are deleterious) for larger gene regions, although this will likely depend on the hypothesis of the study.

In this study we have predominantly used the SKAT-O and MiST tests as they have been reported to be amongst the most consistently powerful collapsing methods according to evaluations of gene-based tests [26]. However, regardless of the choice over collapsing method when undertaking a rare variant analysis, the filtering phase is crucial to identifying association signals from causal variants. Although the 3 bioinformatics tools used in this study all have a similar purpose, the manner in which they accomplish this is quite varied. To predict the effect of non-coding variants, CADD uses a support vector machine with a linear kernel, FATHMM-MKL uses multiple kernel learning and DANN uses a deep neural network. Moreover, even though CADD and DANN use the same training data for their tools, FATHMM-MKL uses pathogenic data from the Human Gene Mutation database [27] and control data from the 1000 genomes project [28]. As a result, the correlation between the annotations from these tools is not that strong, which is why they have all been utilised in this study (CADD & FATHMM-MKL = 0.62, CADD & DANN = 0.74 and DANN & FATHMM-MKL = 0.56 according to dbNSFP v3.0 [29]).

Despite the advances in low coverage sequencing techniques over the last few years, the majority of rare variant analyses applied to these data have been underwhelming[30, 31]. Recently, studies have attempted to improve statistical power in their approaches by increasing their sample sizes[17], adapting current methodological techniques[32] or conducting analyses on remote or isolated populations where the allele frequency of rarer variants may be heightened[33]. We suggest that incorporating predicted functionality of variants into analyses (based on the wealth of functional annotation data now available) should prove to be a valuable and feasible addition to these options in identification of association signals for future studies. Moreover, there has been a greater emphasis recently on elucidating the functional role of variants[34–36], for which filtering approaches using resources such as the bioinformatics tools utilised in this study could be integrated to great effect.

The fundamental question when filtering variants according to predicted function concerns the reliability of the bioinformatics resource used. Clearly, the success of any analysis which relies on these resources hinges on their accuracy. Further advances in the accuracy of variant prediction will have a beneficial impact on association studies which incorporate annotations as we have illustrated in this study. Moreover, filtering variants based on prediction algorithms can be undertaken for analyses conducted in non-coding regions of the genome, which would not be possible when filtering according to variant consequence. The majority of GWAS hits discovered to date fall within non-coding regions[37, 38], although rare variant analyses using collapsing approaches have so far been confined to coding regions of the genome. Therefore, filtering according to non-coding prediction algorithms provides a platform for future studies to investigate the role of variants in these regions, such as investigating the impact of variants in flanking regions of genes which may have an impact on regulatory variation from RNA to protein. This should be of particular interest to future studies given the amount of WGS data currently in development.

## Conclusion

Filtering low frequency and rare variants using knowledge based on molecular function and pathogenicity should help identify strong evidence of association not detected using conventional filtering approaches. Follow up analyses which evaluate these signals will be beneficial in the identification of potential mechanisms and causal variants for complex disease.

## Materials and Methods

### Cohort Description

The UK10K consortium has two main project arms. In this study, we have used data from the cohorts’ arm which was designed to investigate the contribution of genome wide genetic variation to a range of quantitative traits. This arm contains individuals from two intensively studied cohorts of European ancestry, ALSPAC (Avon Longitudinal Study of Parents and Children) and TwinsUK:

#### ALSPAC

ALSPAC is a population-based cohort study investigating genetic and environmental factors that affect the health and development of children. The study methods are described in detail elsewhere[39, 40] (http://www.bristol.ac.uk/alspac).

Ethical approval was obtained from the National Research Ethics Service (NRES) Committee, South East London, REC 2. Written informed consent was obtained from parents for all measurements made.

#### TwinsUK

The TwinsUK registry is a cohort of volunteer adult twins from all over the United Kingdom[41]. Initially, only middle-aged women were recruited and as a result 83% of the registry is female. The registry currently contains 51% monozygotic (MZ) and 49% dizygotic (DZ) twins aged 18–103 years. Further details are available online (http://www.twinsuk.ac.uk/).

Informed consent was obtained from participants before they entered the study and ethical approval was granted by the National Research Ethics Service (NRES) Committee, Westminster, London.

### Sequencing Data

DNA Samples from 4,030 UK10K study participants (2,040 offspring from the ALSPAC cohort, 1,990 from the TwinsUK cohort) were subjected to low coverage (6-8x average read depth) whole-genome sequencing (WGS). Sequencing was performed at both the Wellcome Trust Sanger Institute (WTSI) and the Beijing Genomics Institute (BGI). DNA (1–3μg) was sheared to 100–1000 bp using a Covaris E210 or LE220 (Covaris, Woburn, MA, USA). Sheared DNA was size subjected to Illumina paired-end DNA library preparation. Following size selection (300–500 bp insert size), DNA libraries were sequenced using the Illumina HiSeq platform as paired-end 100 base reads according to manufacturer’s protocol.

Data that passed quality control (QC) was aligned to the GRCh37 human reference used in phase 1 of the 1000 Genomes Project. Reads were aligned using BWA (v0.5.9-r16)[42]. Of the 4,030 participants, 3,910 samples (1,976 ALSPAC and 1,934 TwinsUK) went through the variant calling procedure. Low quality samples were identified by comparing the samples to their GWAS genotypes using about 20,000 sites on chromosome 20. A total of 112 samples (48 ALSPAC and 64 TwinsUK) were removed, leaving 3,798 samples (1,928 ALSPAC and 1,870 TwinsUK) that were eligible for the genotype refinement phase.

Missing and low-confidence genotypes in the filtered VCFs were refined out using the imputation procedure in BEAGLE 4[43] with default parameters. Additional sample-level QC steps were carried out on refined genotypes, resulting in 17 samples (16 TwinsUK and 1 ALSPAC) being removed due to either non-reference discordance with GWAS SNV data>5%, multiple relations to other samples or failed sex check. A principal components analysis was conducted using EIGENSTRAT[44] to exclude participants of non-European ancestry after merging our data with a pruned 11 HapMap3 population dataset[45]. 44 subjects (12 TwinsUK and 32 ALSPAC) did not cluster to the European (CEU) cluster and were removed.

The final sample size for association analyses comprised of 3,621 individuals (1,754 TwinsUK and 1,867 ALSPAC).

### Phenotype data

#### ALSPAC

Height was measured to the nearest 0.1cm using a Harpenden stadiometer (Holtain Crosswell, Dyfed, UK) and weight was measured to the nearest 0.1kg using Tanita electronic scales. Body Mass Index (BMI) was calculated as (weight (kg))/(height (m))2. Blood Pressure was measured with a Dinamap 9301 vital monitor completed by trained staff using the appropriate cuff size. Two readings of both systolic and diastolic blood pressure (SBP & DBP respectively) were taken when the study participants were at rest and the mean of each were used as a measurement in our analysis. Both these measurements were taken from the age 9 clinic (mean age: 9.9, range: 8.9–11.5).

Non-fasting blood samples were also taken from participants who attended the age 9 clinic (mean age: 9.9, range: 8.9–11.5). Plasma lipid concentrations (total cholesterol (TC), triglycerides (TG) and high density lipoprotein cholesterol (HDL)) were measured by modification of the standard Lipid Research Clinics Protocol with enzymatic reagents for lipid determination[46]. Low density lipoprotein cholesterol (LDL) concentration was subsequently calculated using the Friedwald equation[47]:

LDL=TC–(HDLc+TG×0.45)

#### TwinsUK

Height was measured to the nearest 0.5cm using a wall-mounted stadiometer and weight (light clothing only) was measured to the nearest 0.1kg using digital scales. Body Mass Index (BMI) was calculated as (weight (kg))/(height (m))^2^. Brachial blood pressure was measured using an automated cuff sphygmomanometer (OMRON HEM713C; Omron Healthcare (UK) Ltd, Henfield, UK). SBP and DBP were measured three times, two of which were highly correlated (0.90 for SBP and 0.92 for DBP) and averaged to get our final phenotype measurements.

Blood samples were taken after at least 6 hours of overnight fasting. The samples were immediately inverted three times and left to rest for 40 minutes at 4°C to obtain complete coagulation. The samples were then centrifuged for 10 min at 2000g and serum was removed. Four aliquots of 1.5 ml were placed into skirted micro centrifuge tubes and then stored in a -45°C freezer until sampling[48]. A colorimetric enzymatic method was used to determine TC, TG and HDL levels. The Friedewald equation was used to calculate LDL levels in subjects.

### Statistical Analysis

We used CADD[9] to obtain scaled C scores for all 44.9 million possible variants and indels in the UK10K whole genome sequence data[14]. After removing variants which failed QC, we filtered all variants in three ways 1) responsible for a ‘nonsynonymous’ substitution according to the Variant Effect Predictor[15] (VEP) 2) responsible for a ‘loss of function’ according to VEP (i.e. ‘stop losses/gains’, ‘splice sites’ or ‘frameshift indels’) and 3) Variants with a CADD C-Score of 15 or higher. We collapsed variants together across candidate genes and analysed them using SKAT[8] with their associated traits according to Liu et al[17]. These genes were *ANGPTL4*, *BCAM*, *CBLC*, *CD300LG*, *HNF4A*, *LDLR*, *LIPC*, *LIPG*, *LPL*, *PCSK9* and *PVR*. Analyses were repeated after applying each variant filtering method, as well as applying two different minor allele frequency cutoffs (MAF≤5%) and (MAF≤1%).

We also identified variants with a CADD C-Score ≥15 across the genome and aggregated them together across all gene regions according to UCSC definitions (reference genome hg19). Variants were then collapsed together and analysed with 7 cardiovascular traits (BMI, SBP, DBP, TC, HDL, LDL & TG) using SKAT after applying a MAF cutoff of 5%. We repeated this process in a second set of analyses using a MAF cutoff of 1% to investigate rarer variation.

Gene-based p-values from the CADD analyses were matched with p-values from the ‘nonsynonymous’ filtered analysis and the results were–log10 transformed and plotted using hexbin plots for each trait and MAF cutoff. This meant that only genes which had at least 2 variants within their region after filtering in both sets of analyses (i.e. ≥ 2 ‘nonsynonymous’ & ≥ 2 variants with a CADD C-Score ≥15) were plotted on these graphs. This was undertaken using the ‘hexbin’ package in R [18] This process was repeated except matching on results from the ‘loss-of-function’ analysis. As this resulted in far fewer data points to be plotted, scatter plots were generated instead using using the R package ‘ggplot2’ [49]. All plots were also regenerated using the results of an identical analysis except using the SKAT-O [19] and MiST tests [20]. This approach was repeated except using two alternative bioinformatics tools to CADD which can also predict the effect of variants in both coding and non-coding regions of the genome (FATHMM-MKL [21] and DANN [22]). R statistical software[50] was used for all statistical analyses and plots.

## Supporting Information

S1 File Gene based low frequency and rare variant analyses using various variant filtering approaches and collapsing methods. (PDF)
