# Supplementary material for: Incorporating Non-Coding Annotations into Rare Variant Analysis
Source: PLoS One. 2016 Apr 29;11(4):e0154181. doi: 10.1371/journal.pone.0154181 (PMC4851421; doi:10.1371/journal.pone.0154181)
Supplement: S1 File — (PDF) [file pone.0154181.s001.pdf]

Supporting Information

S1 Figure: Gene-based low frequency variant analyses across the genome using SKAT for 3 cardiovascular traits (compared with nonsynonymous filtering)

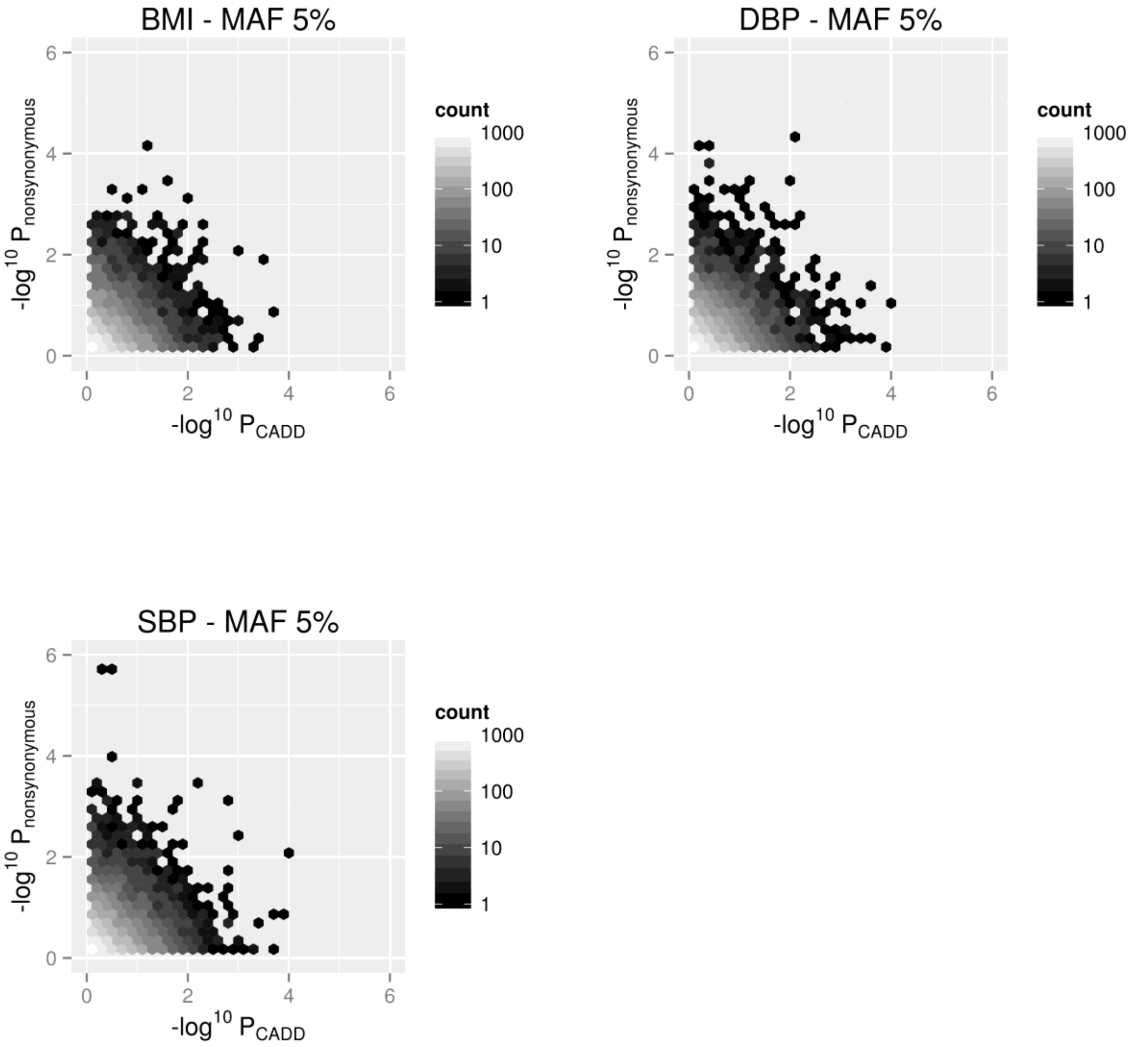

S2 Figure: Gene-based rare variant analyses across the genome using SKAT for 3 cardiovascular traits (compared with nonsynonymous filtering)

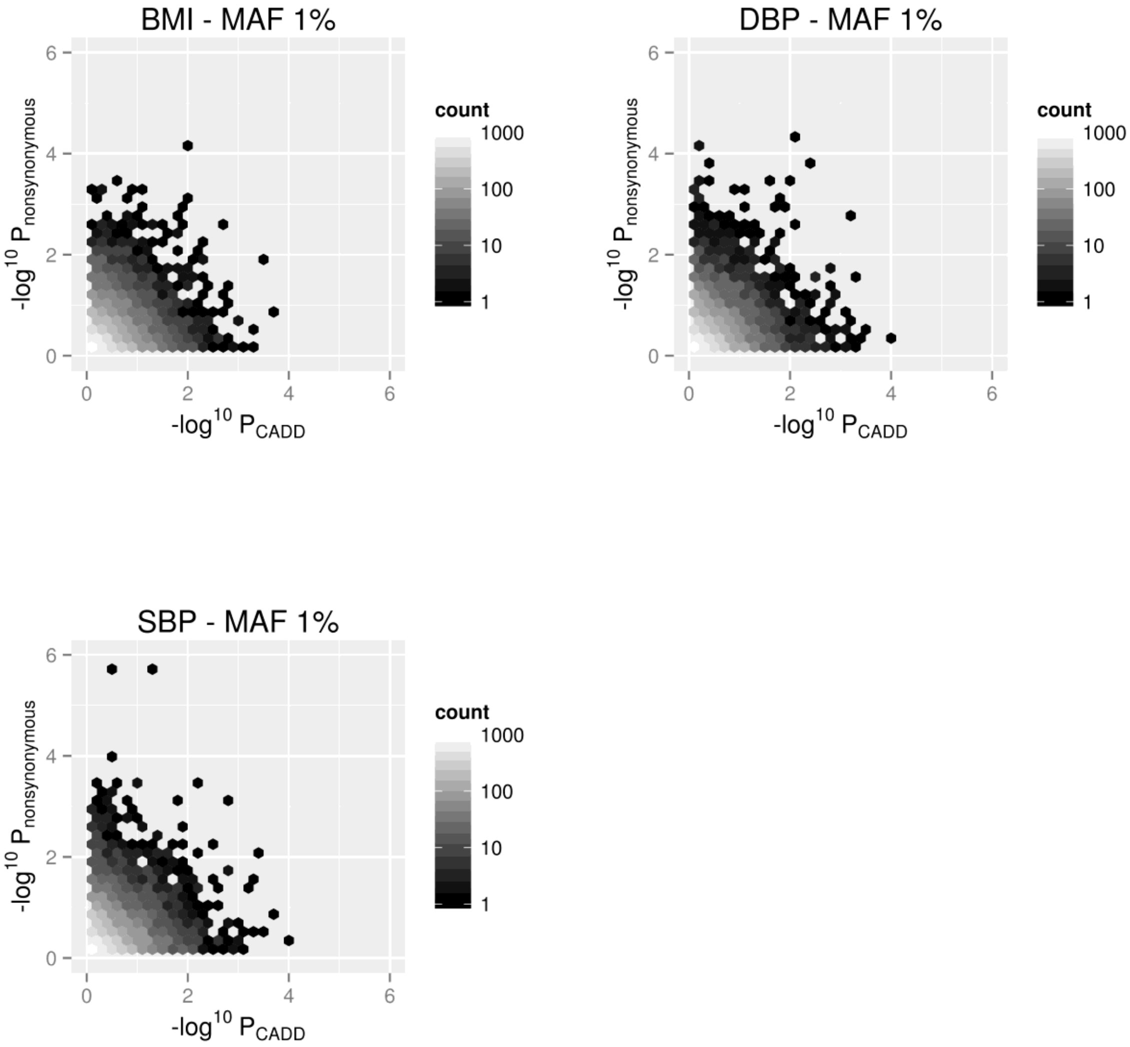

S3 Figure: Gene-based low frequency variant analyses across the genome using SKAT for 7 cardiovascular traits (compared with loss-of-function filtering)

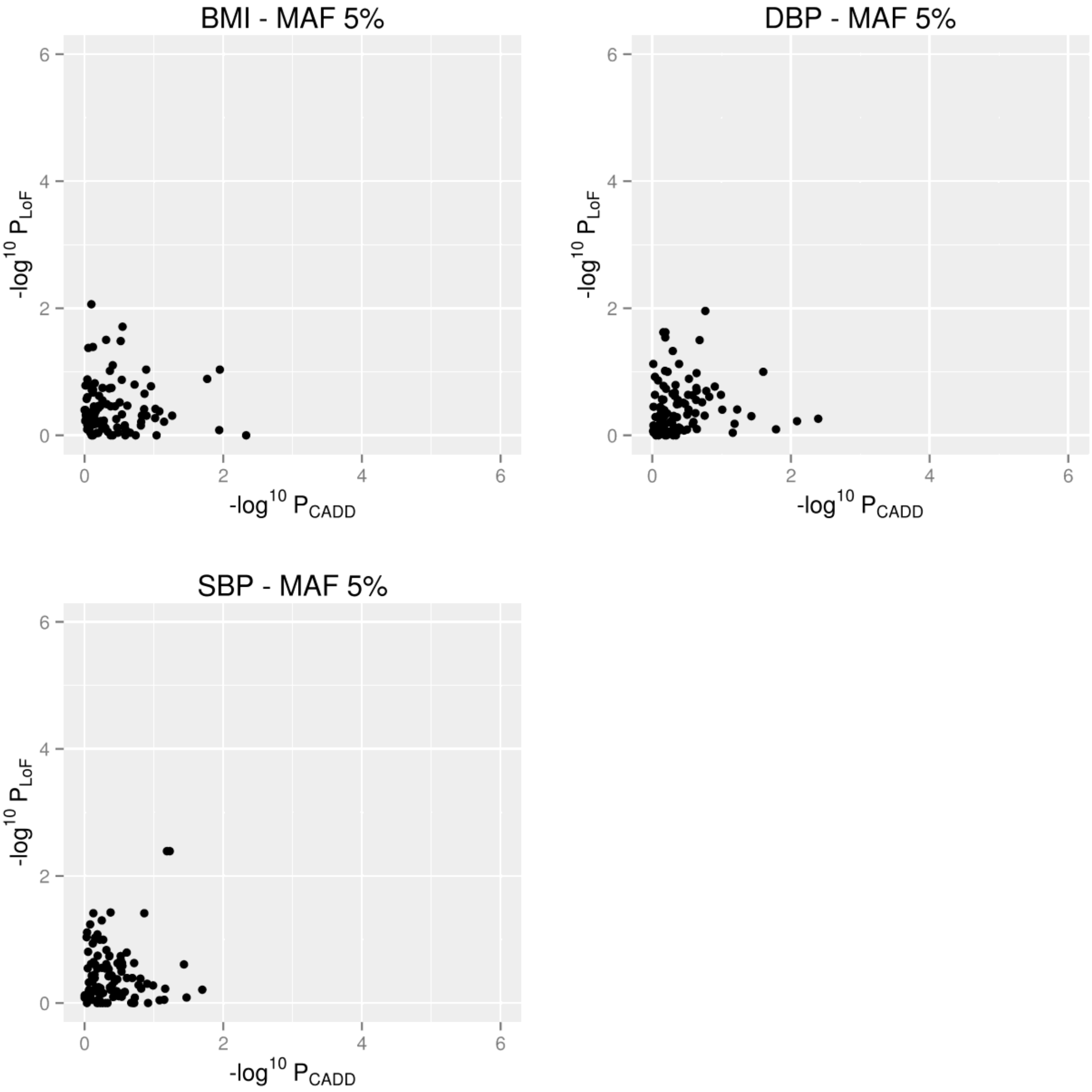

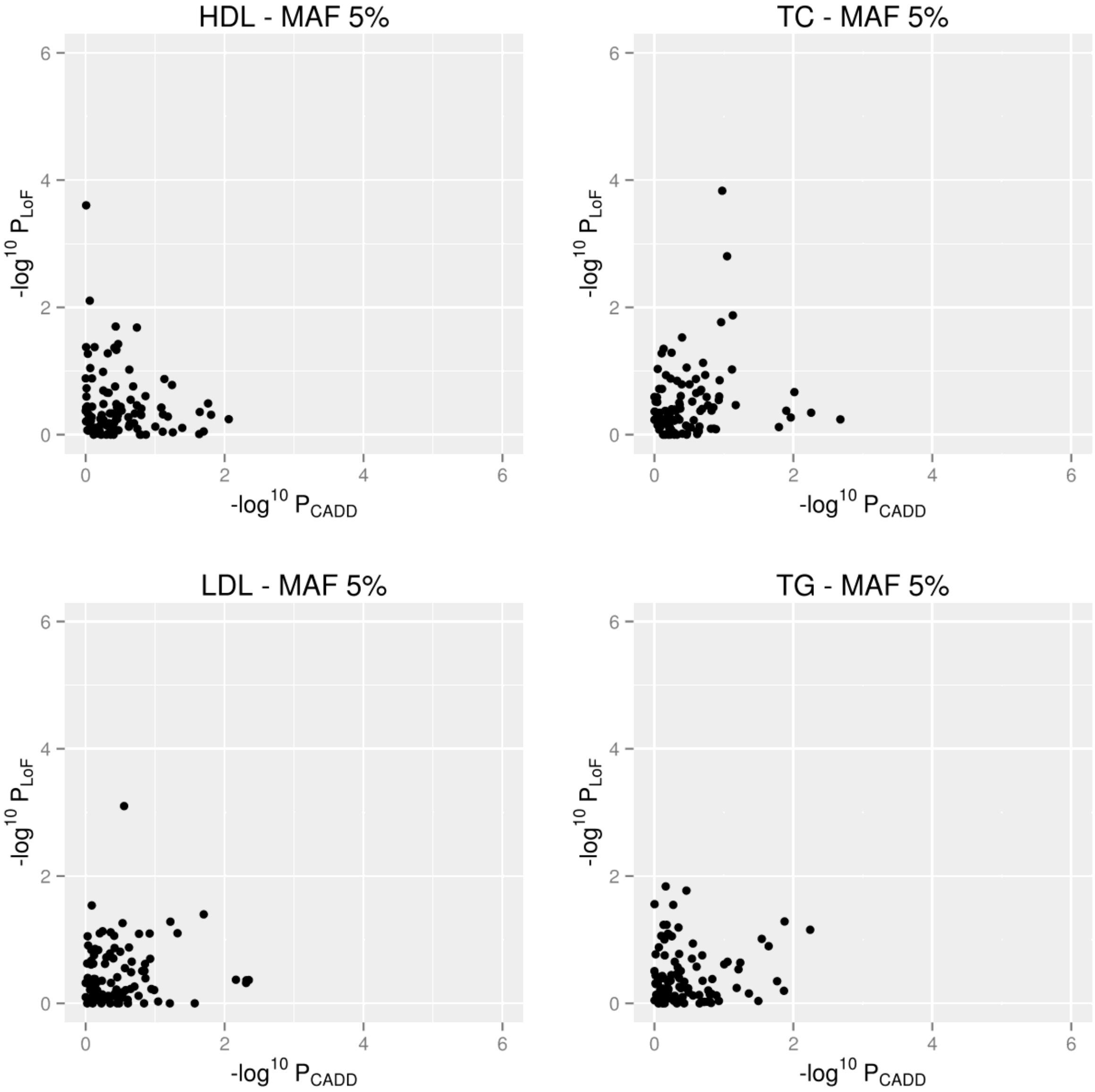
HDL - MAF 5%
TC - MAF 5%
LDL - MAF 5%
TG - MAF 5%
$-\log^{10} P_{LoF}$
$-\log^{10} P_{CADD}$
0
2
4
6

S4 Figure: Gene-based rare variant analyses across the genome using SKAT for 7 cardiovascular traits (compared with loss-of-function filtering)

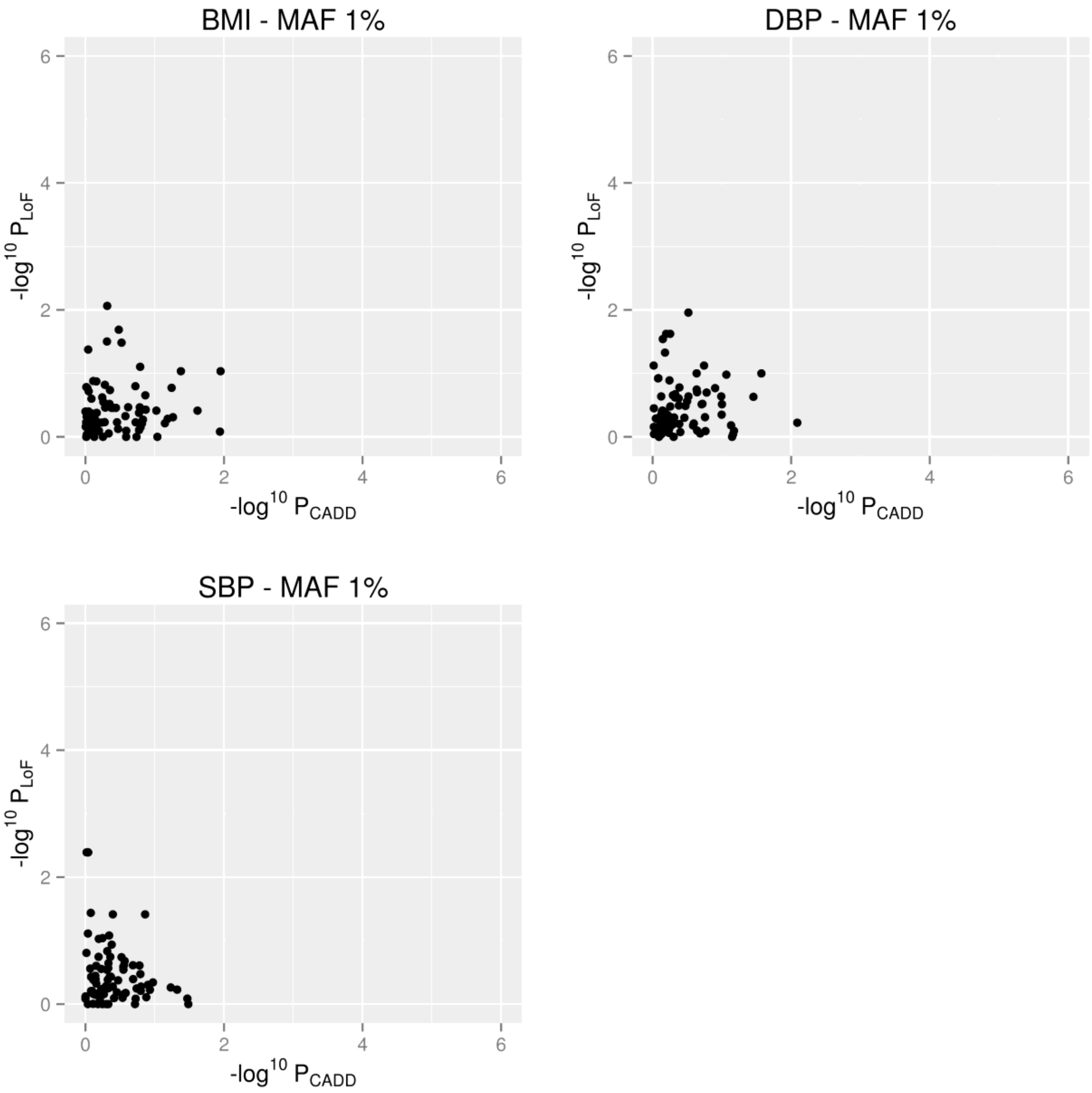

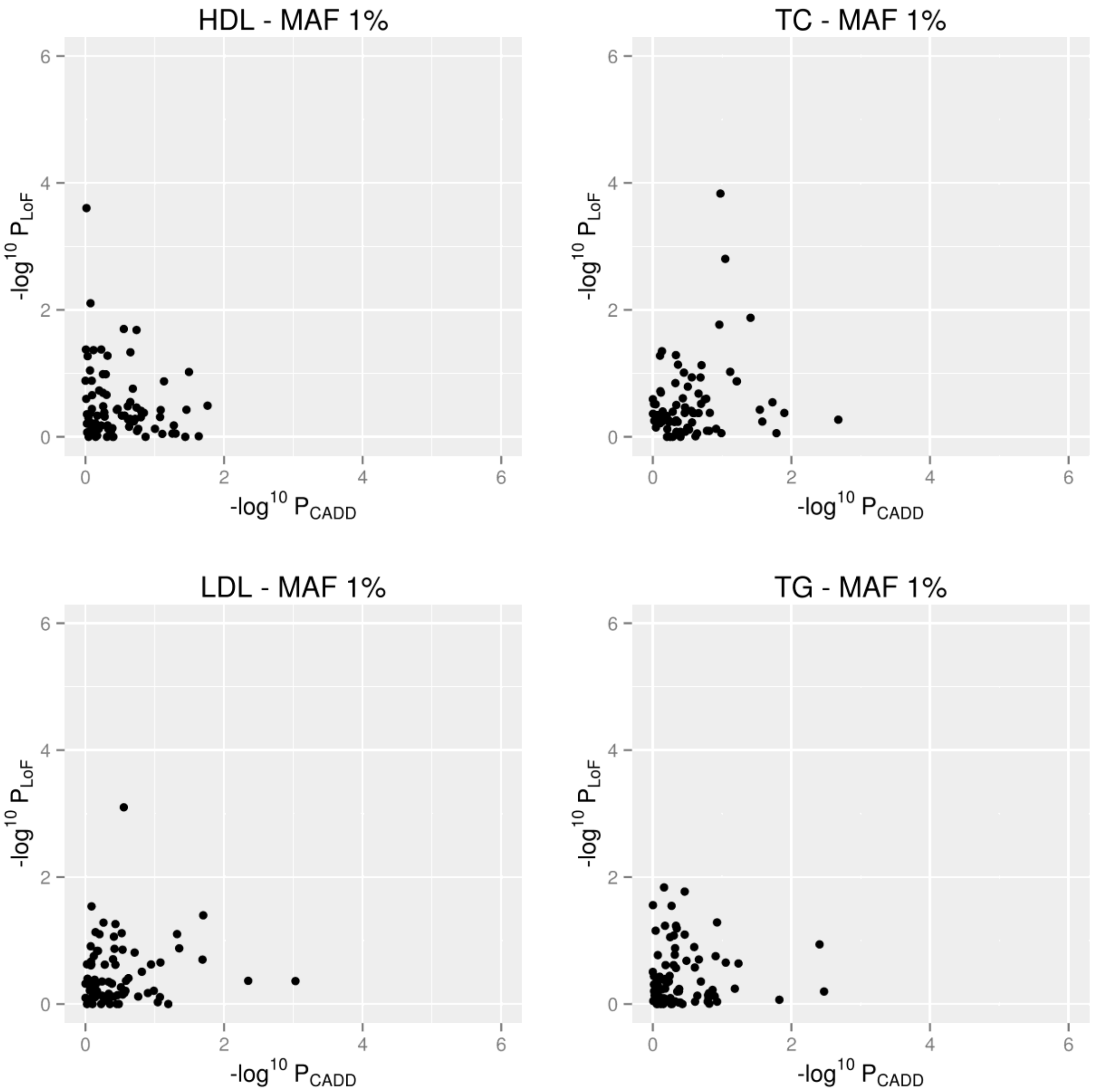

HDL - MAF 1%
TC - MAF 1%
LDL - MAF 1%
TG - MAF 1%
$-\log^{10} P_{LoF}$
$-\log^{10} P_{CADD}$
0
2
4
6

S5 Figure: Gene-based low frequency variant analyses across the genome using SKAT-O for 7 cardiovascular traits (compared with nonsynonymous filtering)

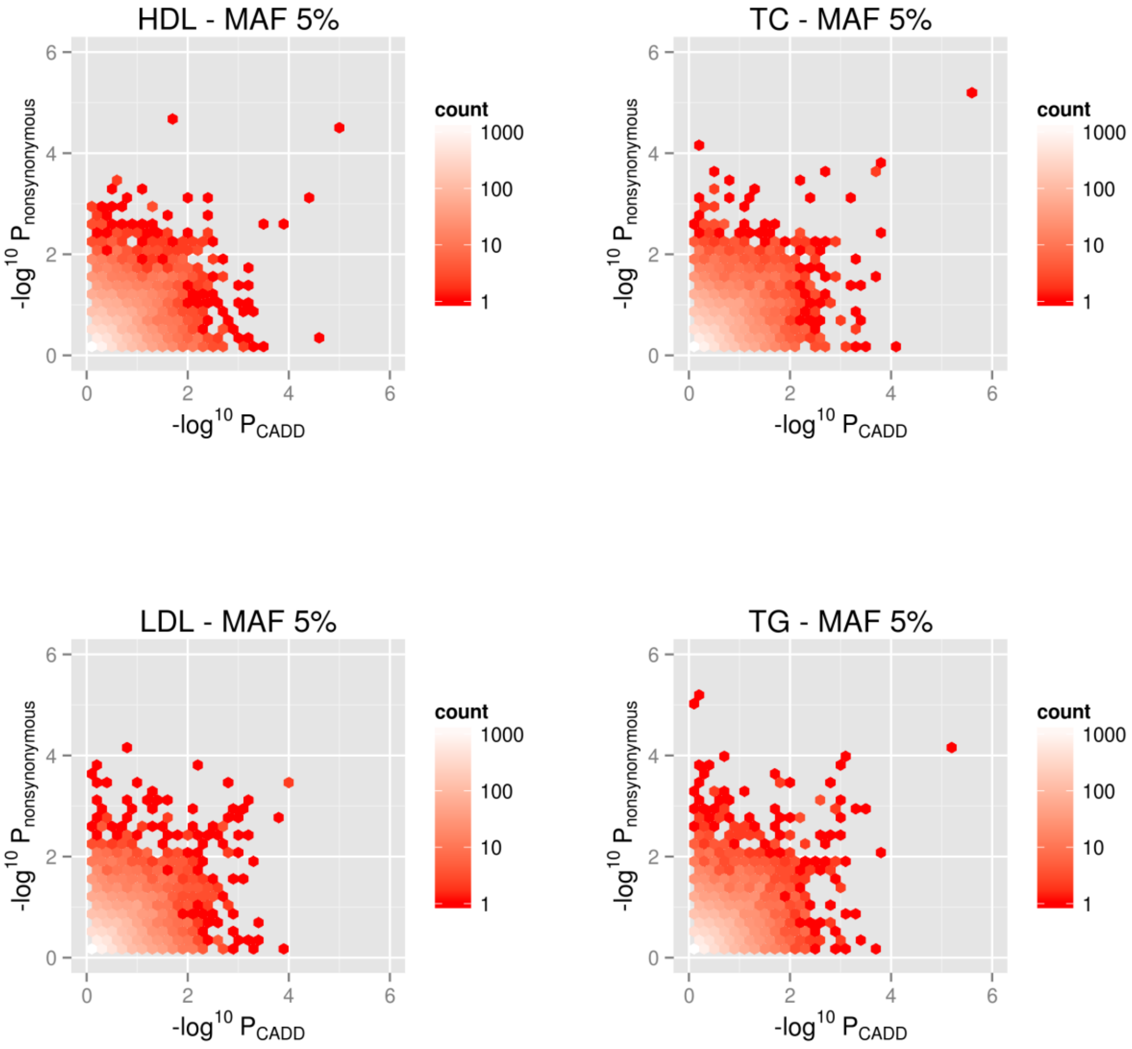

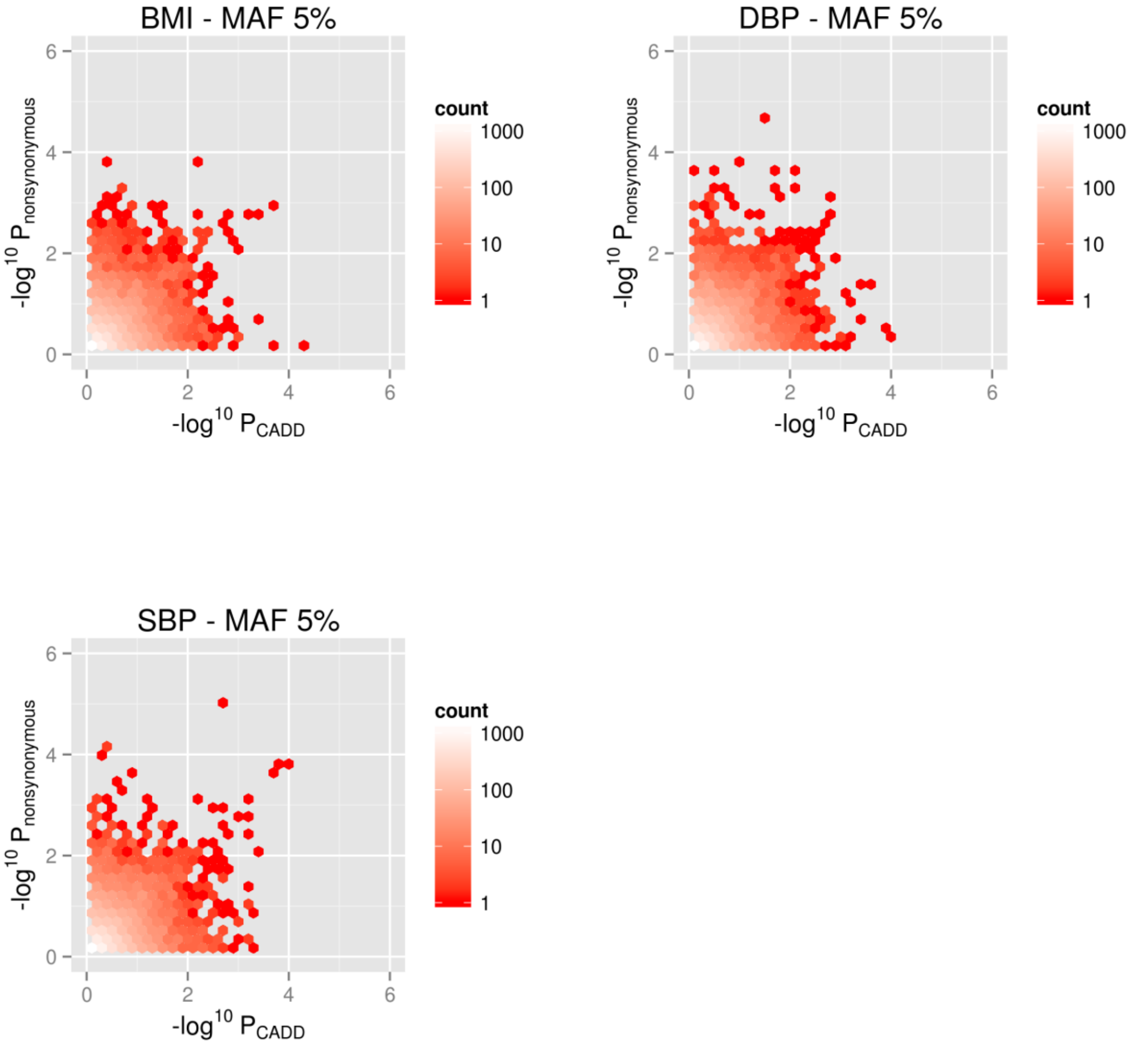

BMI - MAF 5%
DBP - MAF 5%
SBP - MAF 5%
-log10 P nonsynonymous
-log10 P CADD
count
1000
100
10
1

S6 Figure: Gene-based rare variant analyses across the genome using SKAT-O for 7 cardiovascular traits (compared with nonsynonymous filtering)

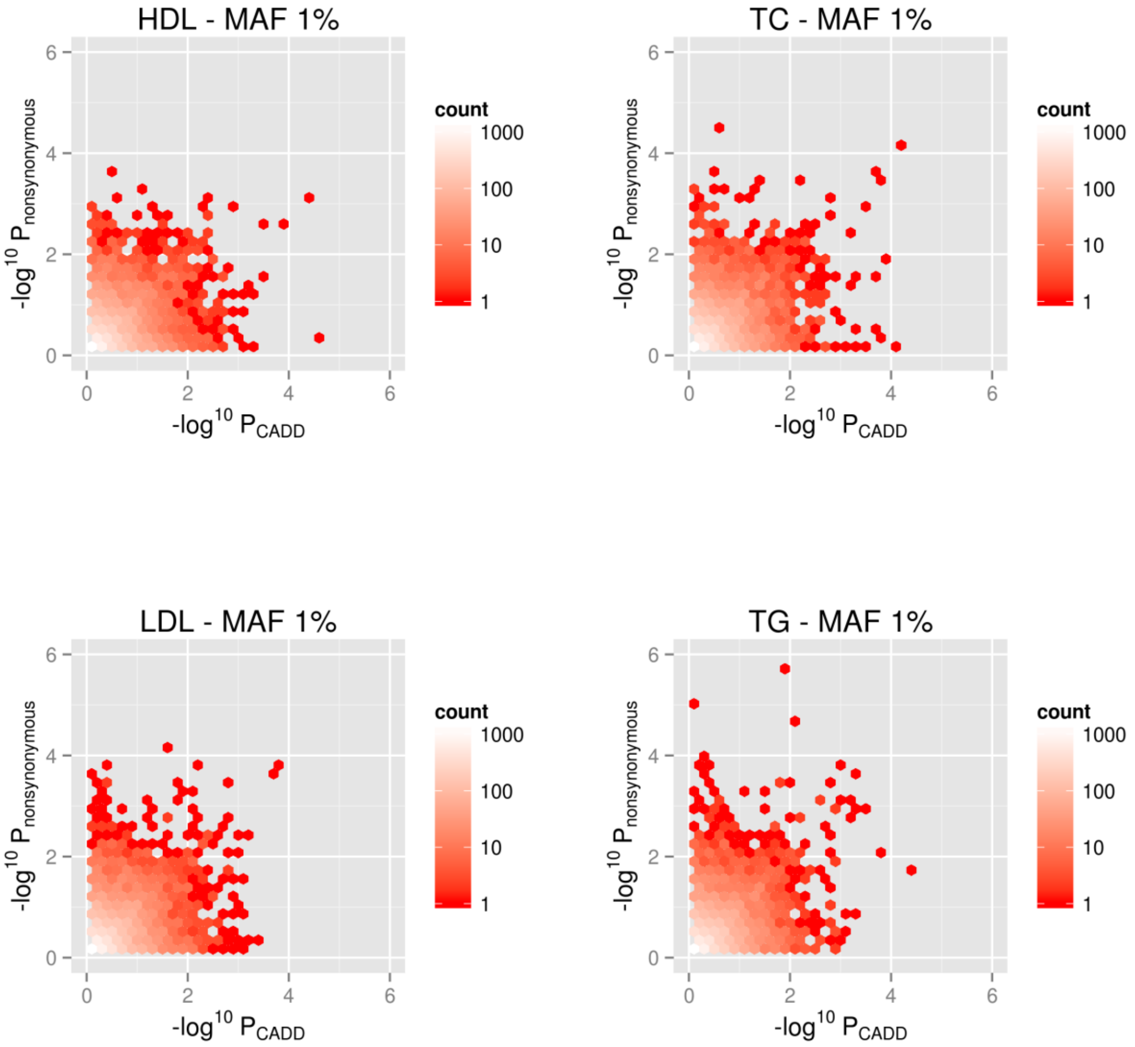

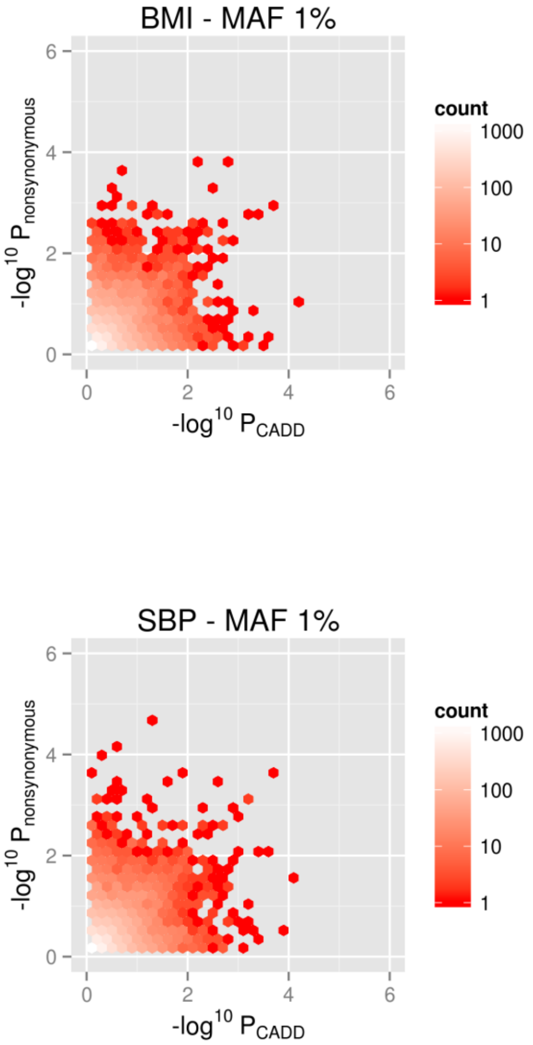

BMI - MAF 1%
DBP - MAF 1%
SBP - MAF 1%
$-\log^{10} P_{nonsynonymous}$
$-\log^{10} P_{CADD}$
0
2
4
6
count
1000
100
10
1

S7 Figure: Gene-based low frequency variant analyses across the genome using SKAT-O for 7 cardiovascular traits (compared with loss-of-function filtering)

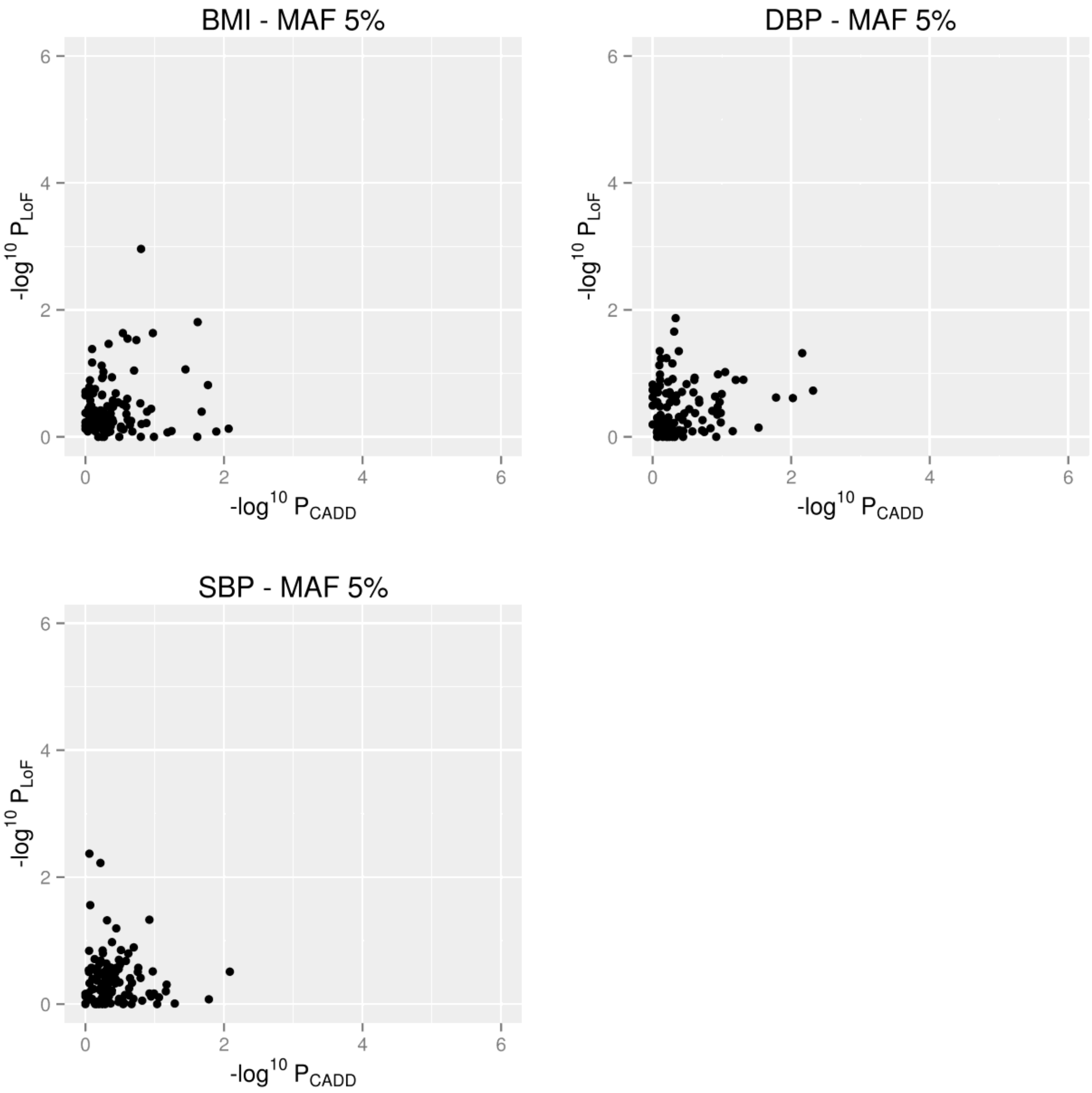

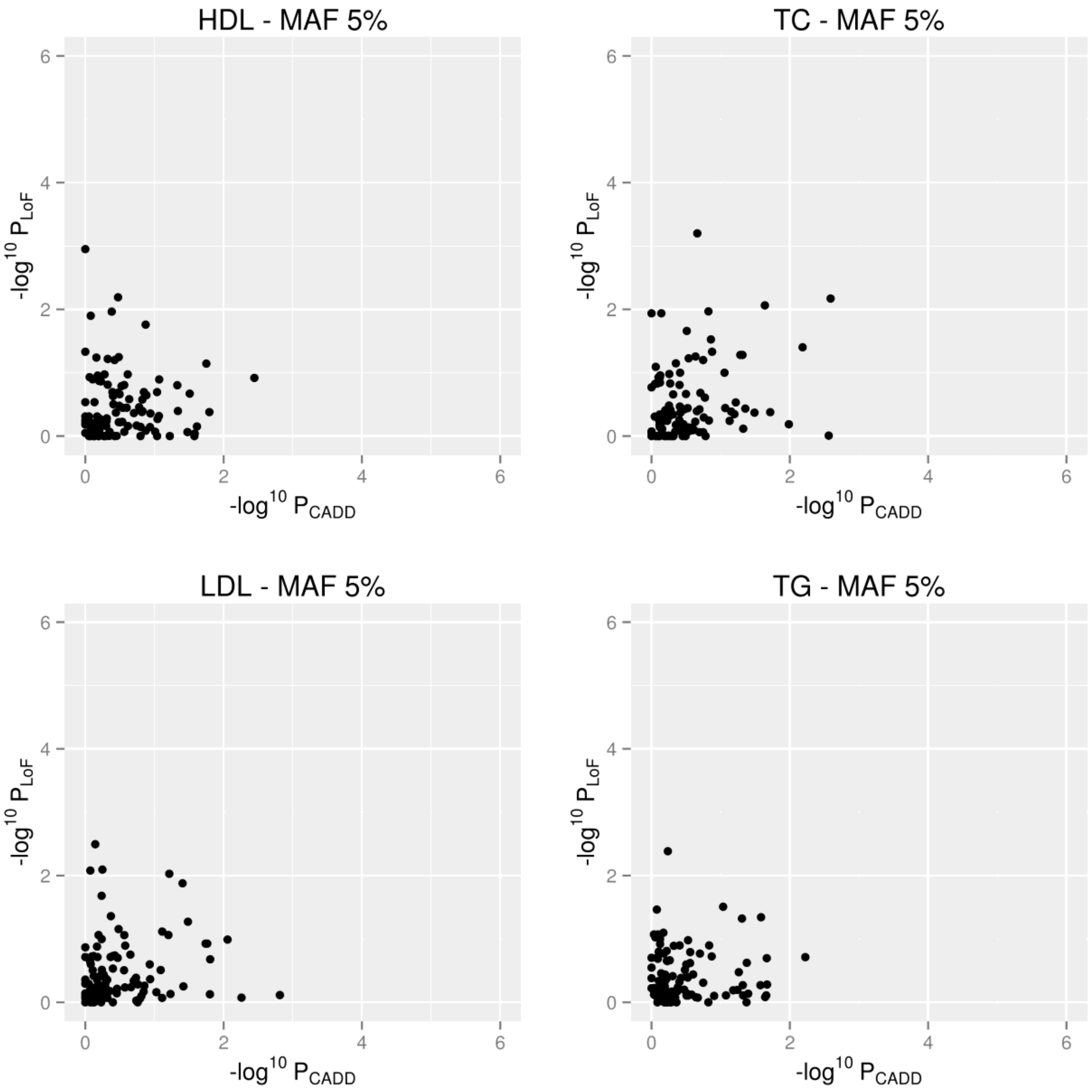

HDL - MAF 5%
TC - MAF 5%
LDL - MAF 5%
TG - MAF 5%
$-\log^{10} P_{LoF}$
$-\log^{10} P_{CADD}$
0
2
4
6

S8 Figure: Gene-based rare variant analyses across the genome using SKAT-O for 7 cardiovascular traits (compared with loss-of-function filtering)

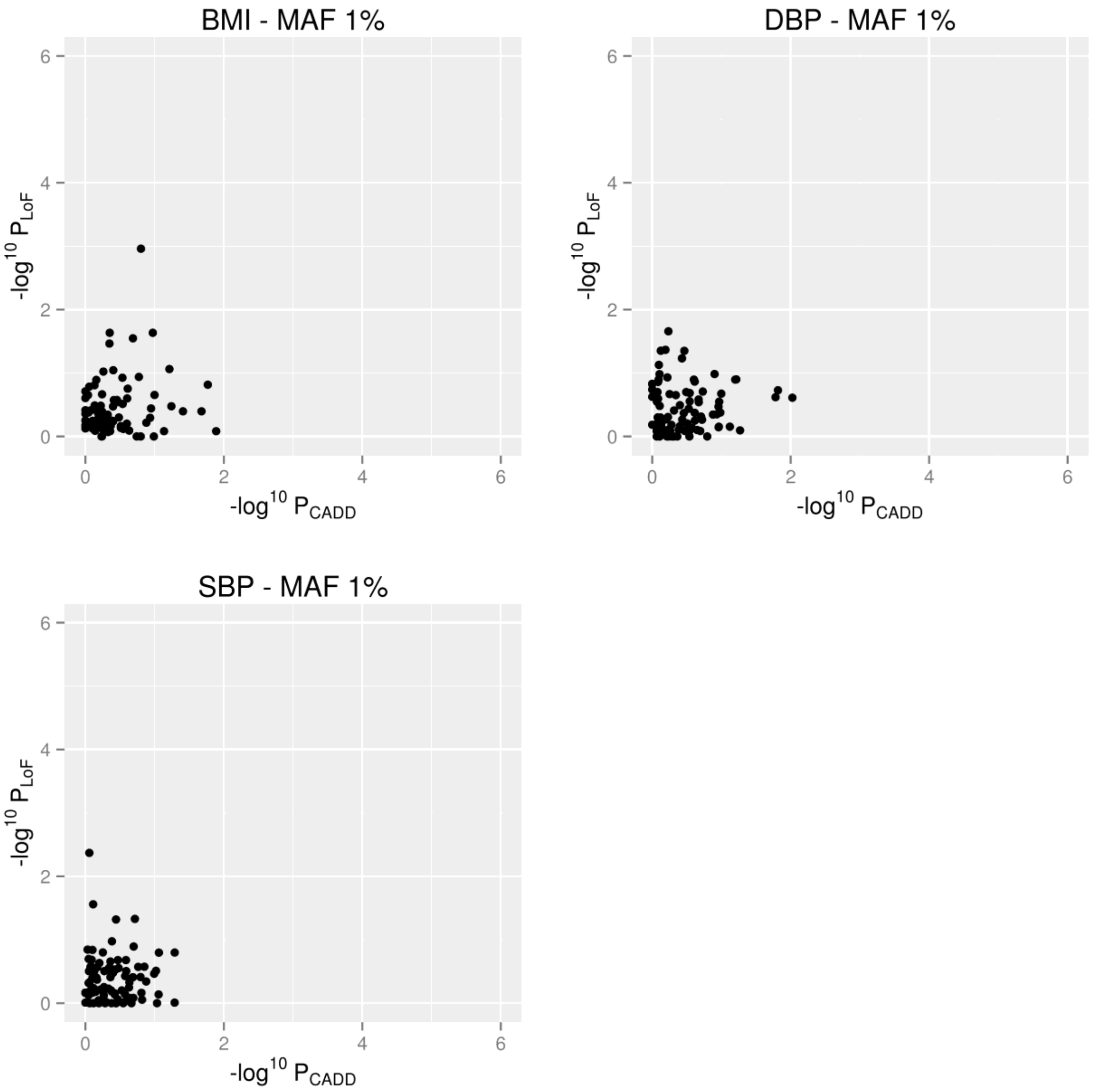

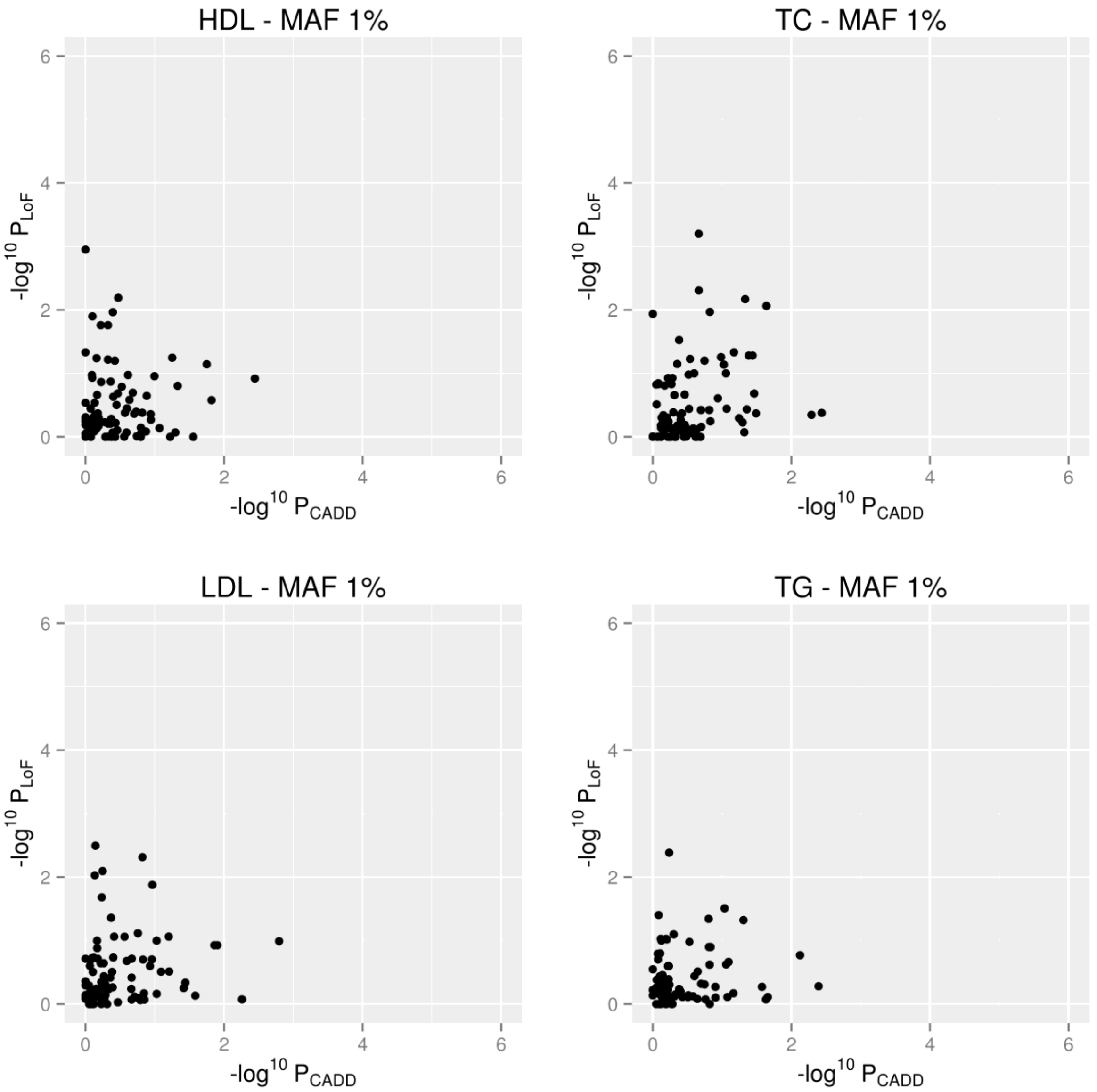
HDL - MAF 1%
TC - MAF 1%
LDL - MAF 1%
TG - MAF 1%
$-\log^{10} P_{LoF}$
$-\log^{10} P_{CADD}$
0
2
4
6

S9 Figure: Gene-based low frequency variant analyses across the genome using MiST for 7 cardiovascular traits (compared with nonsynonymous filtering)

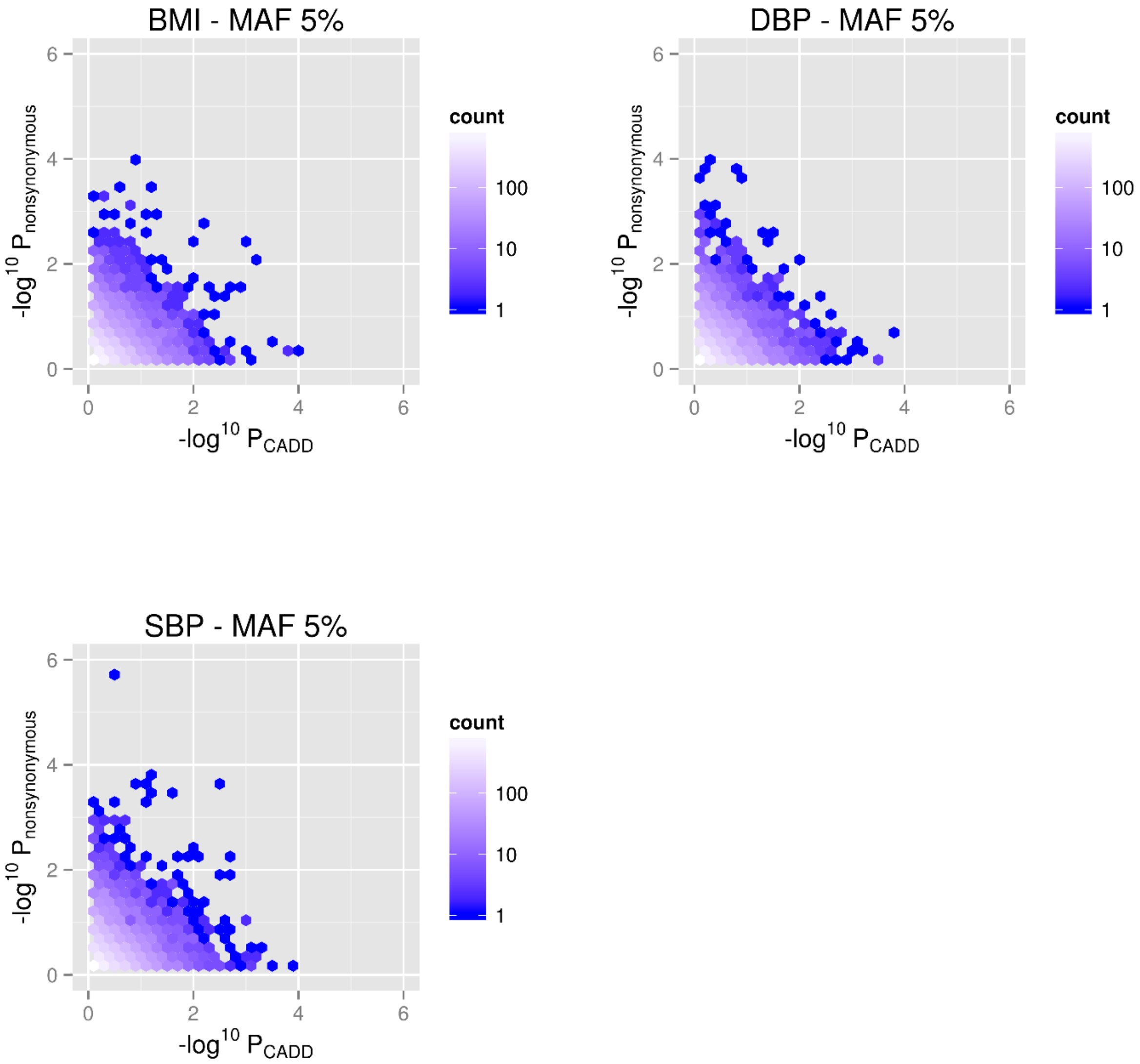

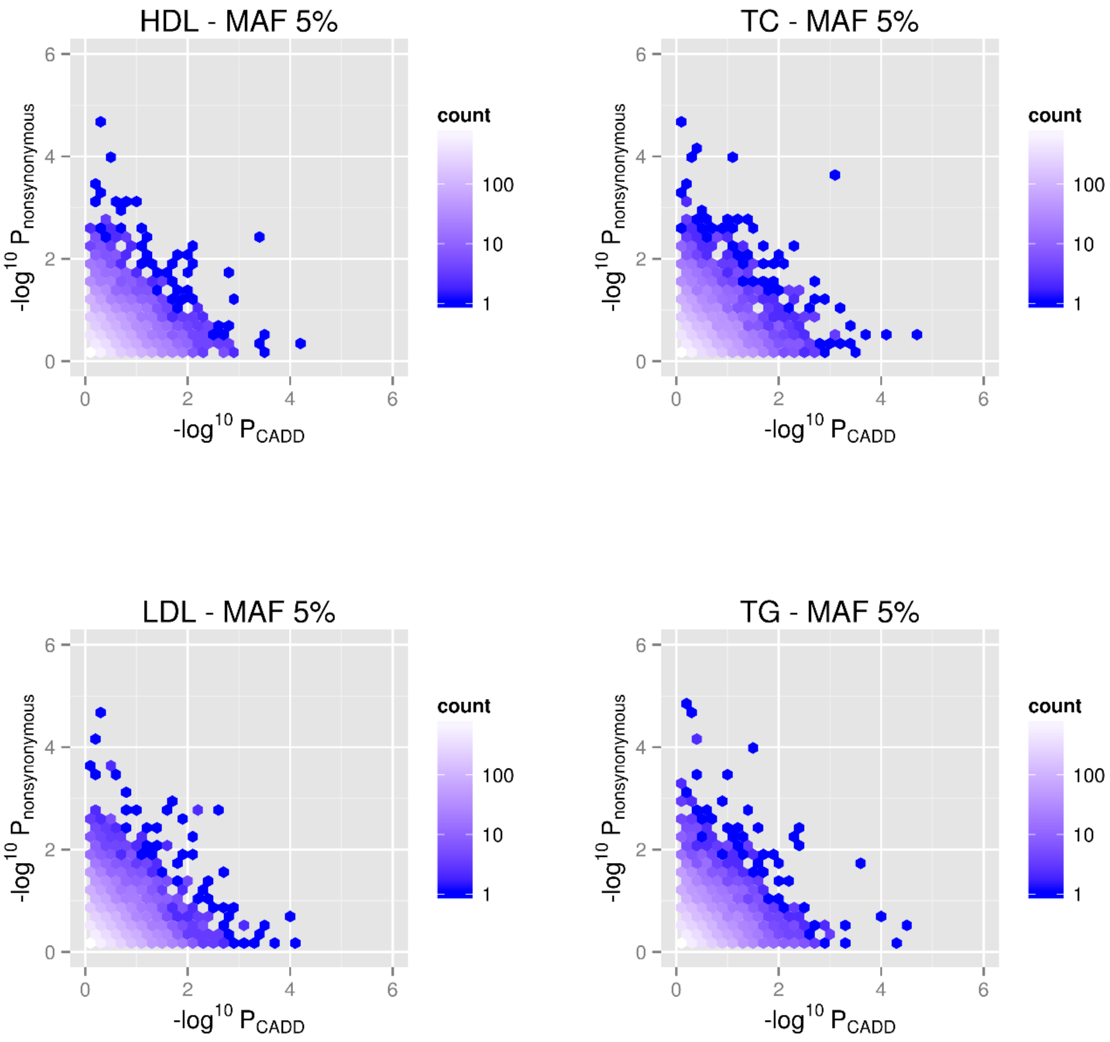
HDL - MAF 5%
TC - MAF 5%
LDL - MAF 5%
TG - MAF 5%
count
100
10
1
-log10 P nonsynonymous
-log10 P CADD
0
2
4
6

S10 Figure: Gene-based rare variant analyses across the genome using MiST for 7 cardiovascular traits (compared with nonsynonymous filtering)

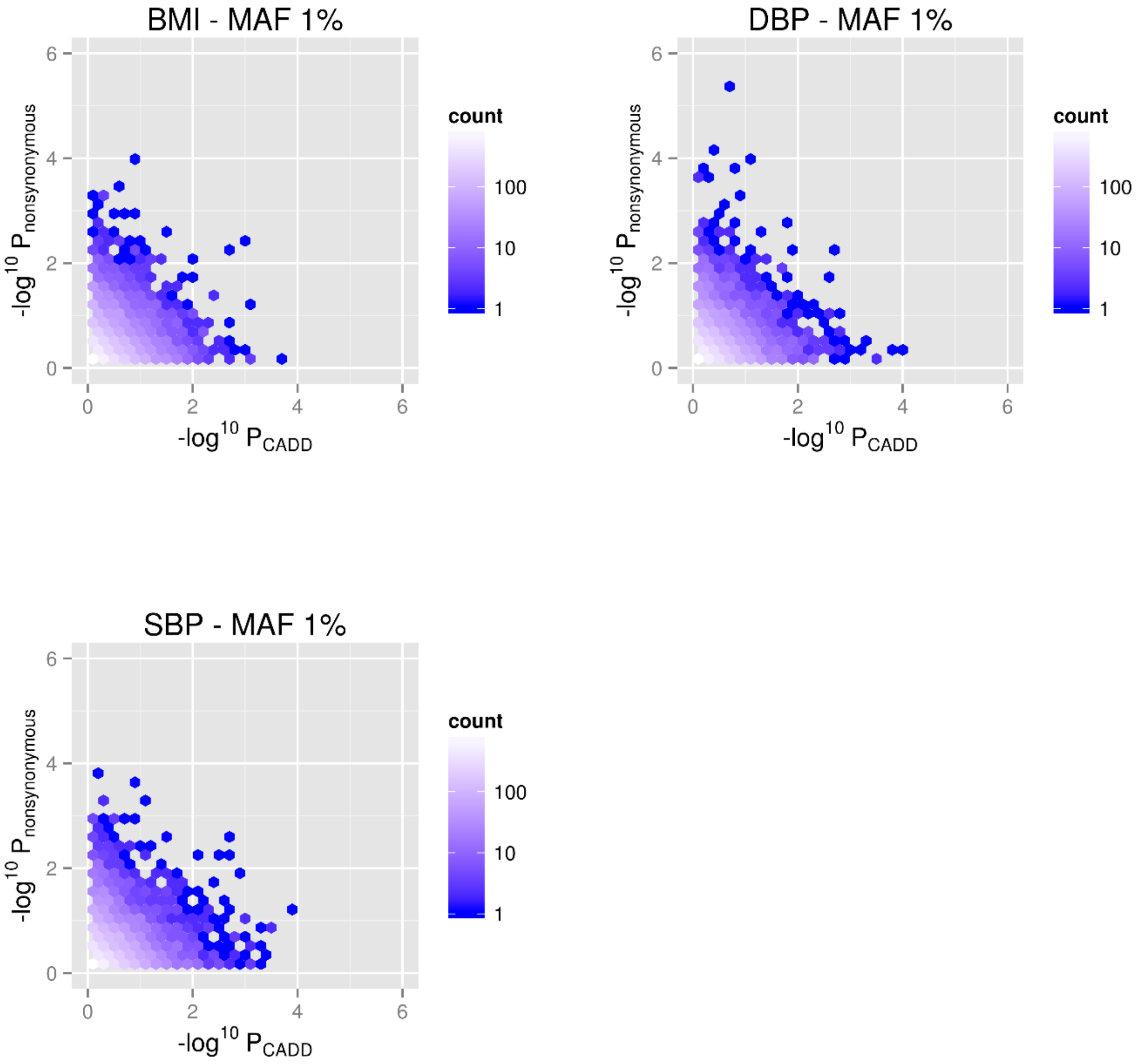

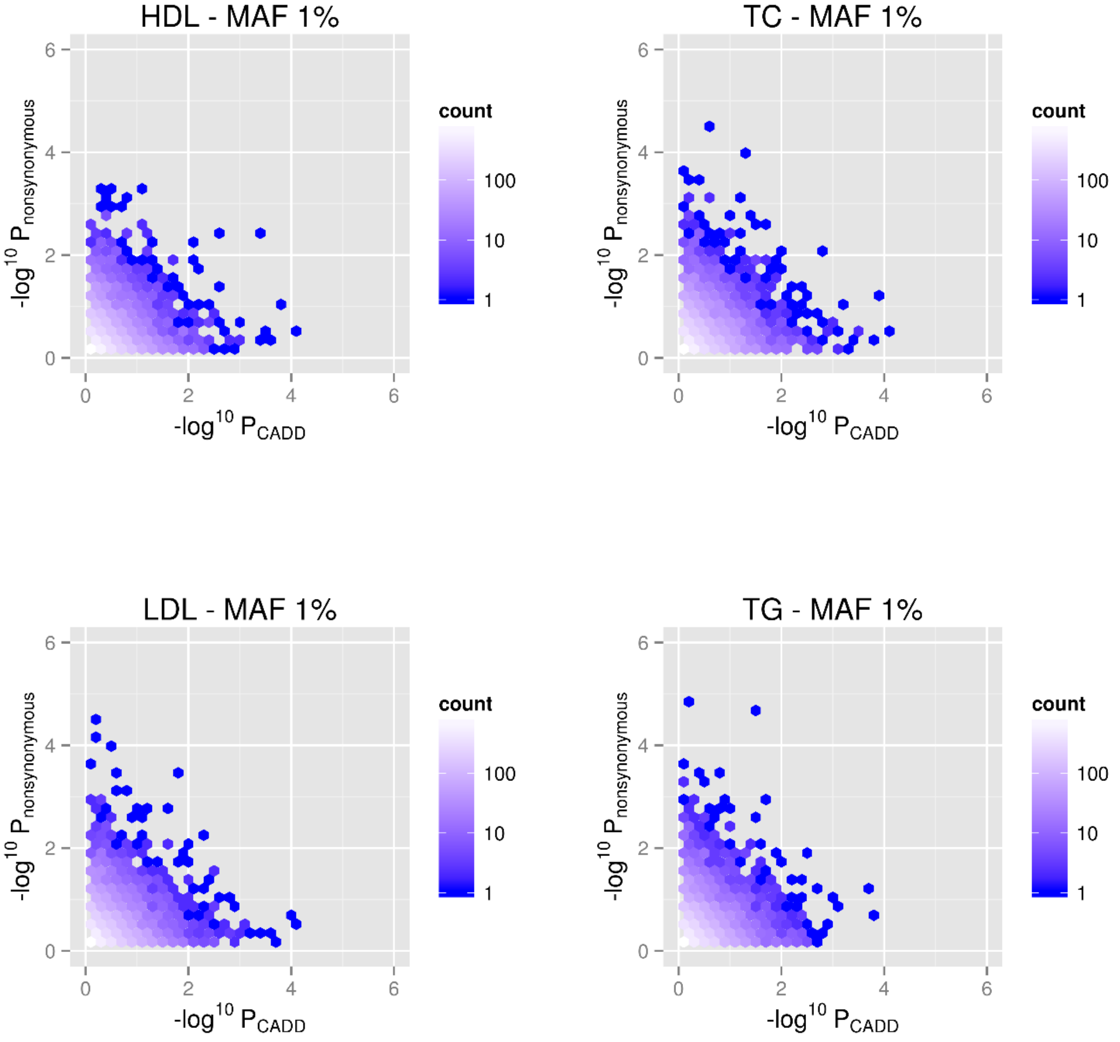

HDL - MAF 1%
TC - MAF 1%
LDL - MAF 1%
TG - MAF 1%
-log10 P nonsynonymous
-log10 P CADD
count
100
10
1
0
2
4
6

S11 Figure: Gene-based low frequency variant analyses across the genome using MiST for 7 cardiovascular traits (compared with loss-of-function filtering)

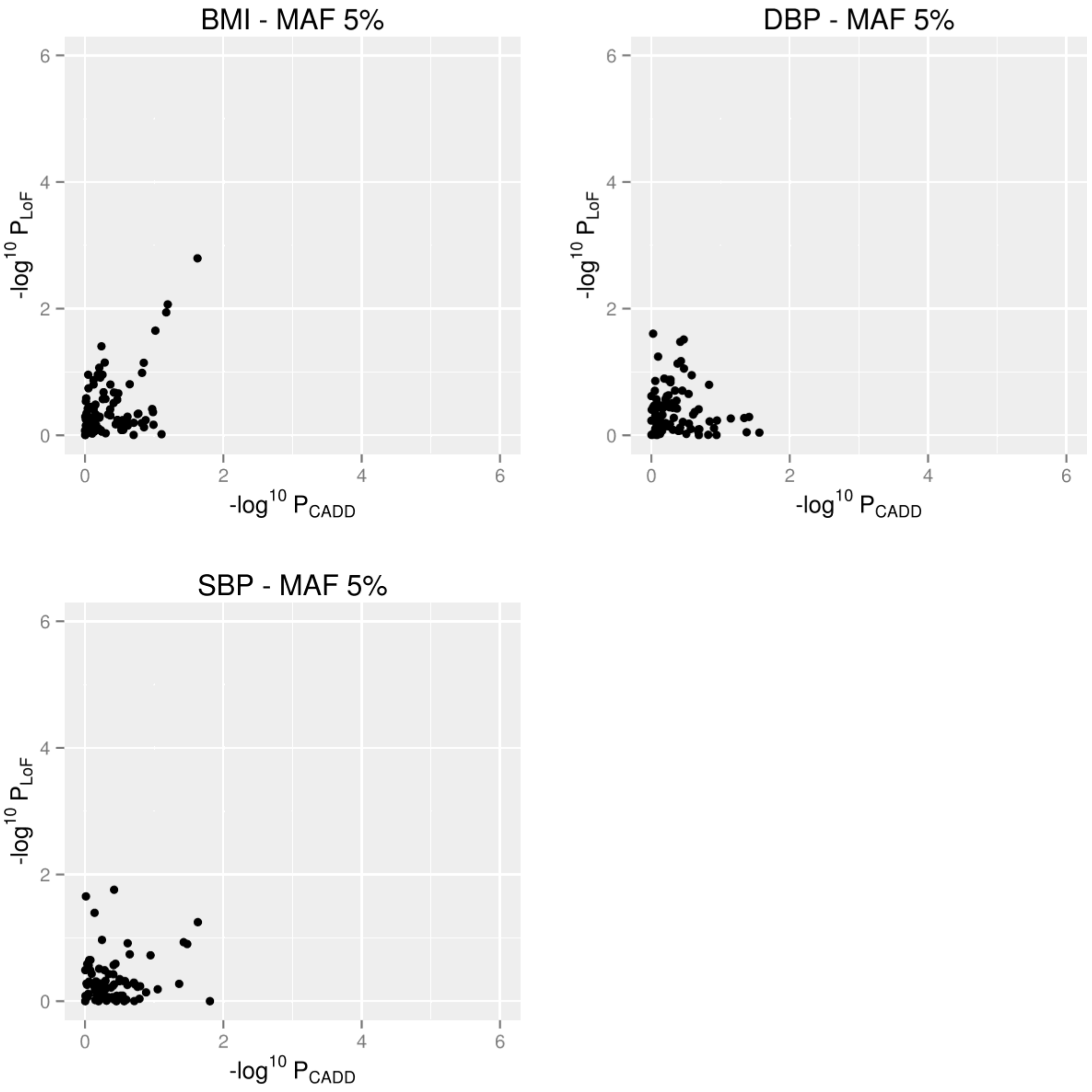

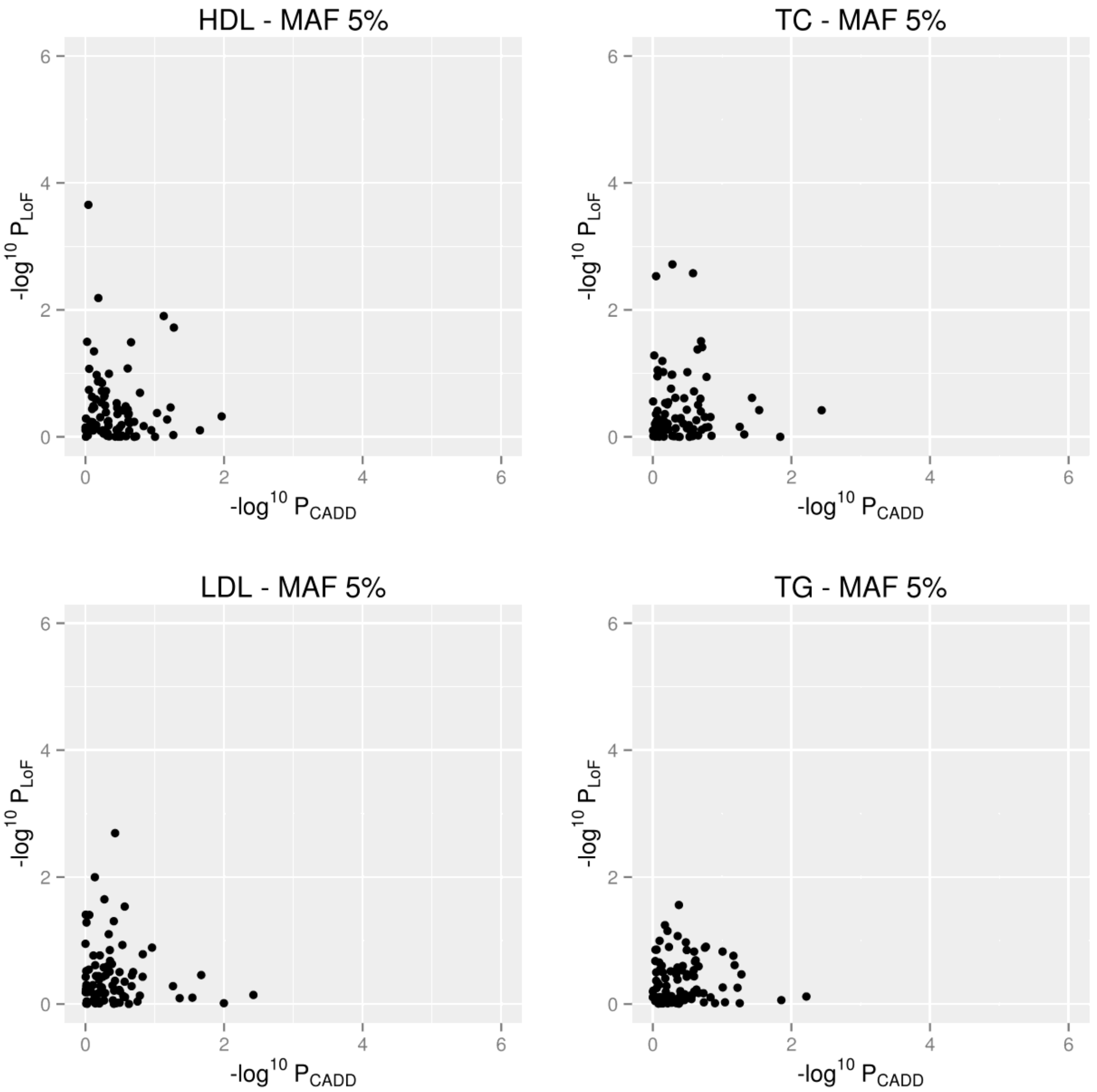

HDL - MAF 5%
TC - MAF 5%
LDL - MAF 5%
TG - MAF 5%
-log10 P_LoF
-log10 P_CADD
0
2
4
6

S12 Figure: Gene-based rare variant analyses across the genome using MiST for 7 cardiovascular traits (compared with loss-of-function filtering)

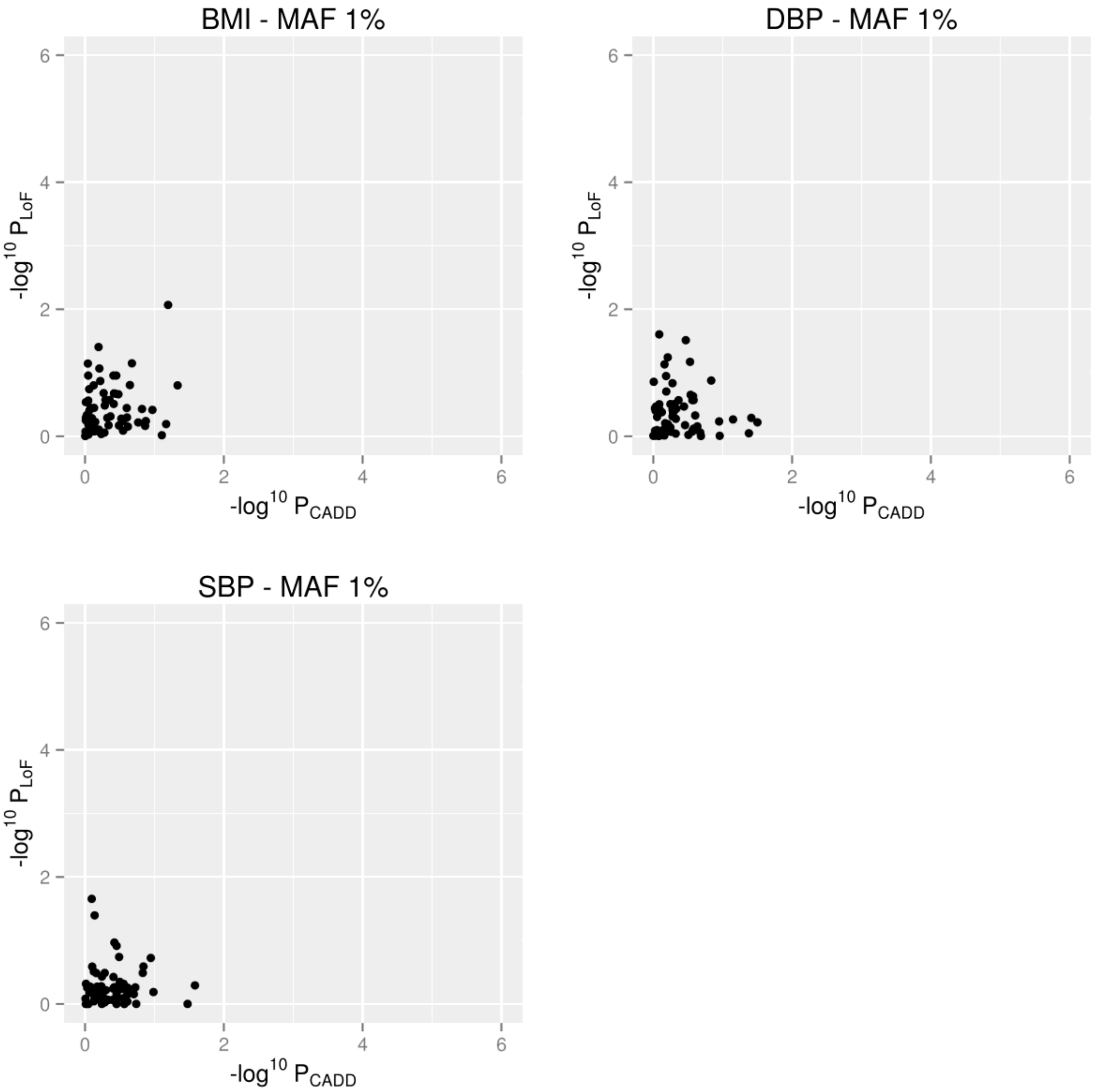

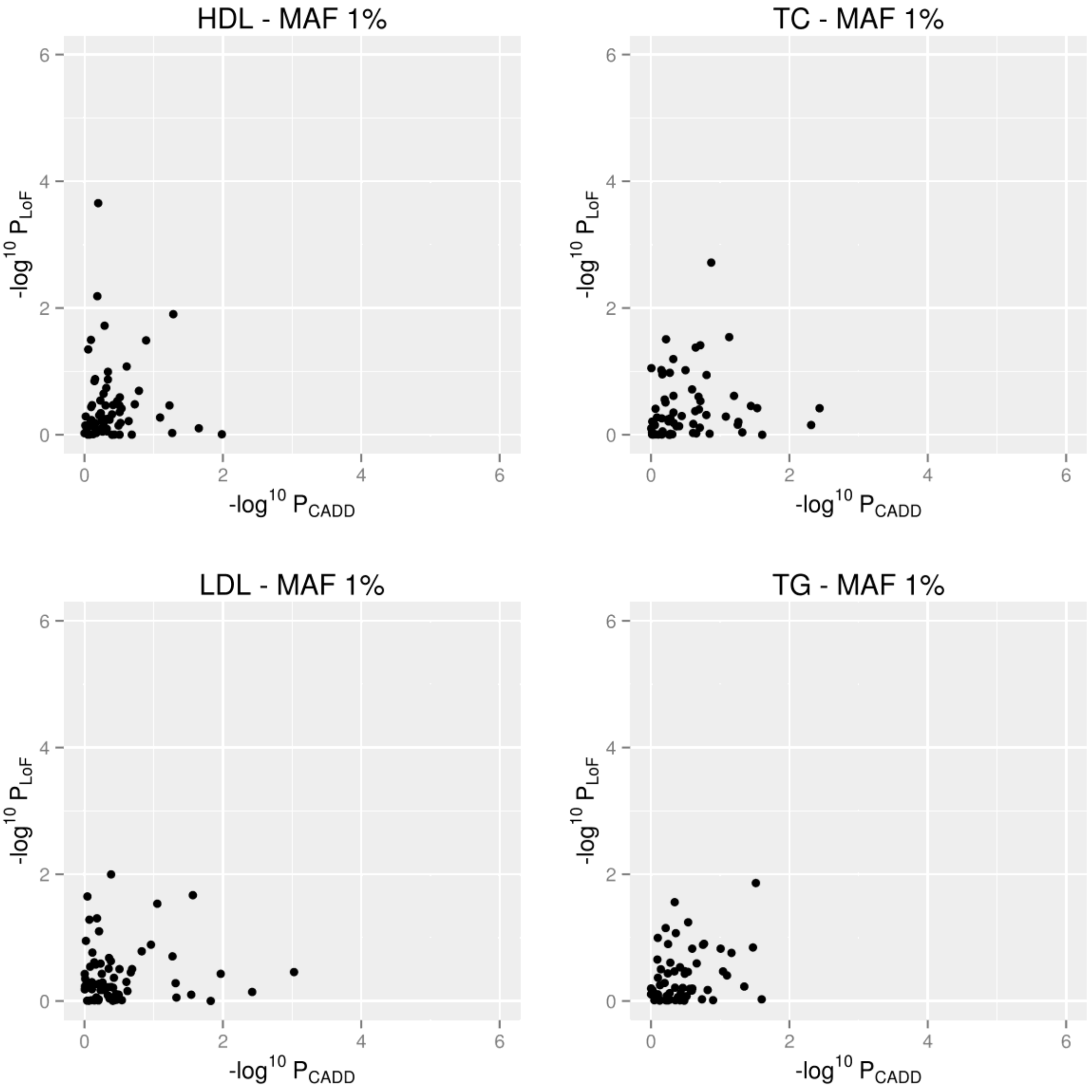

HDL - MAF 1%
TC - MAF 1%
LDL - MAF 1%
TG - MAF 1%
-log10 PLoF
-log10 PCADD
0
2
4
6

FATHMM-MKL tests

S13 Figure: Gene-based low frequency variant analyses across the genome using SKAT-O for 7 cardiovascular traits (compared with nonsynonymous filtering)

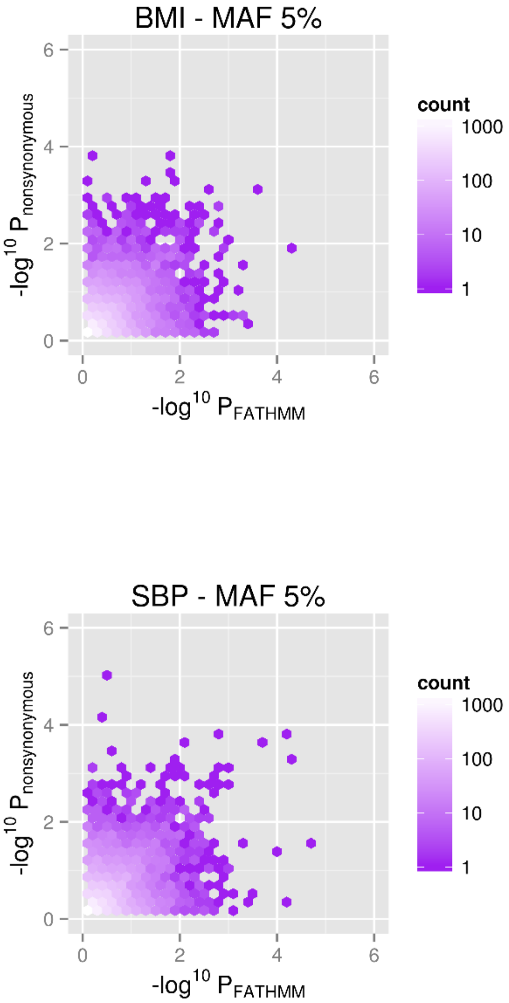

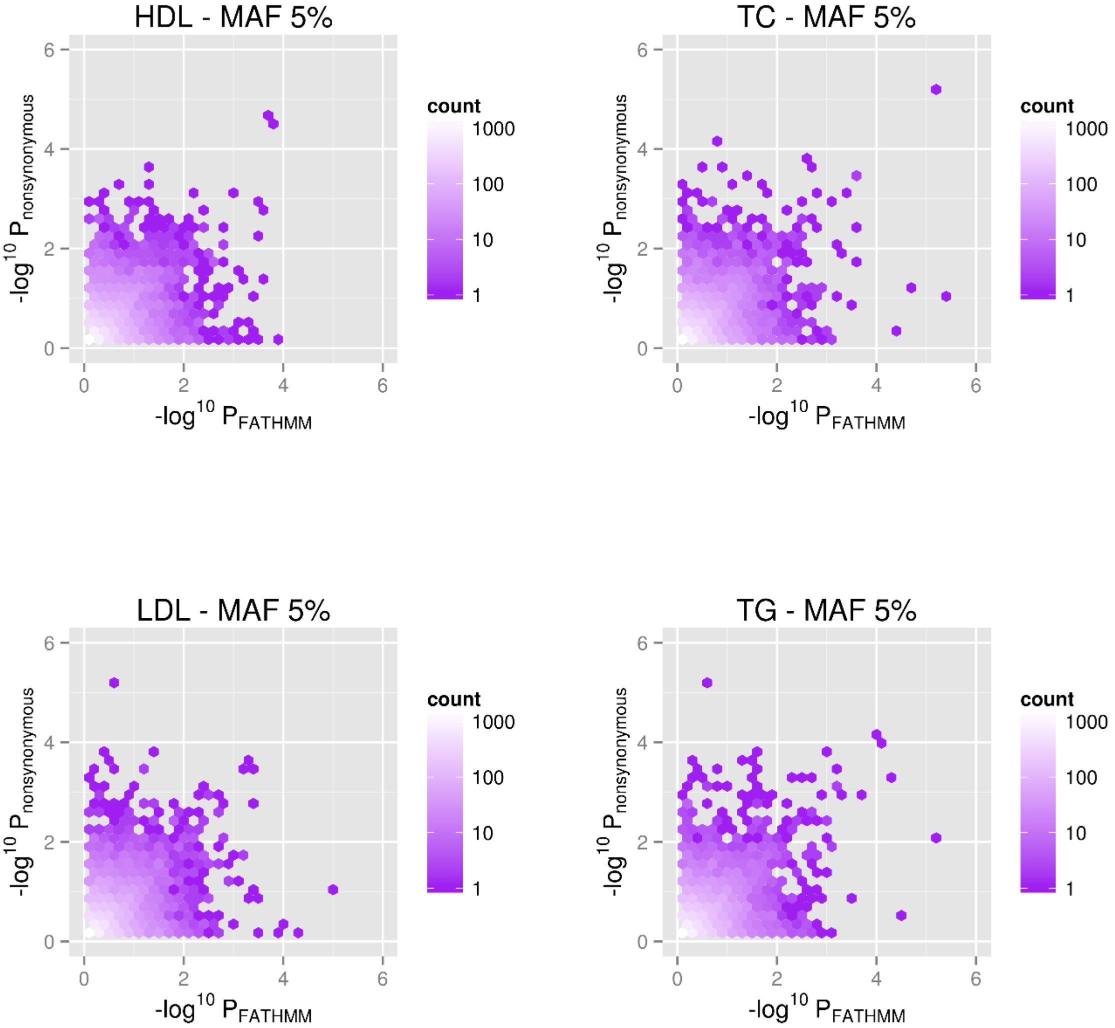

HDL - MAF 5%
TC - MAF 5%
LDL - MAF 5%
TG - MAF 5%
count
1000
100
10
1
-log10 P nonsynonymous
-log10 P FATHMM
0
2
4
6

S14 Figure: Gene-based rare variant analyses across the genome using SKAT-O for 7 cardiovascular traits (compared with nonsynonymous filtering)

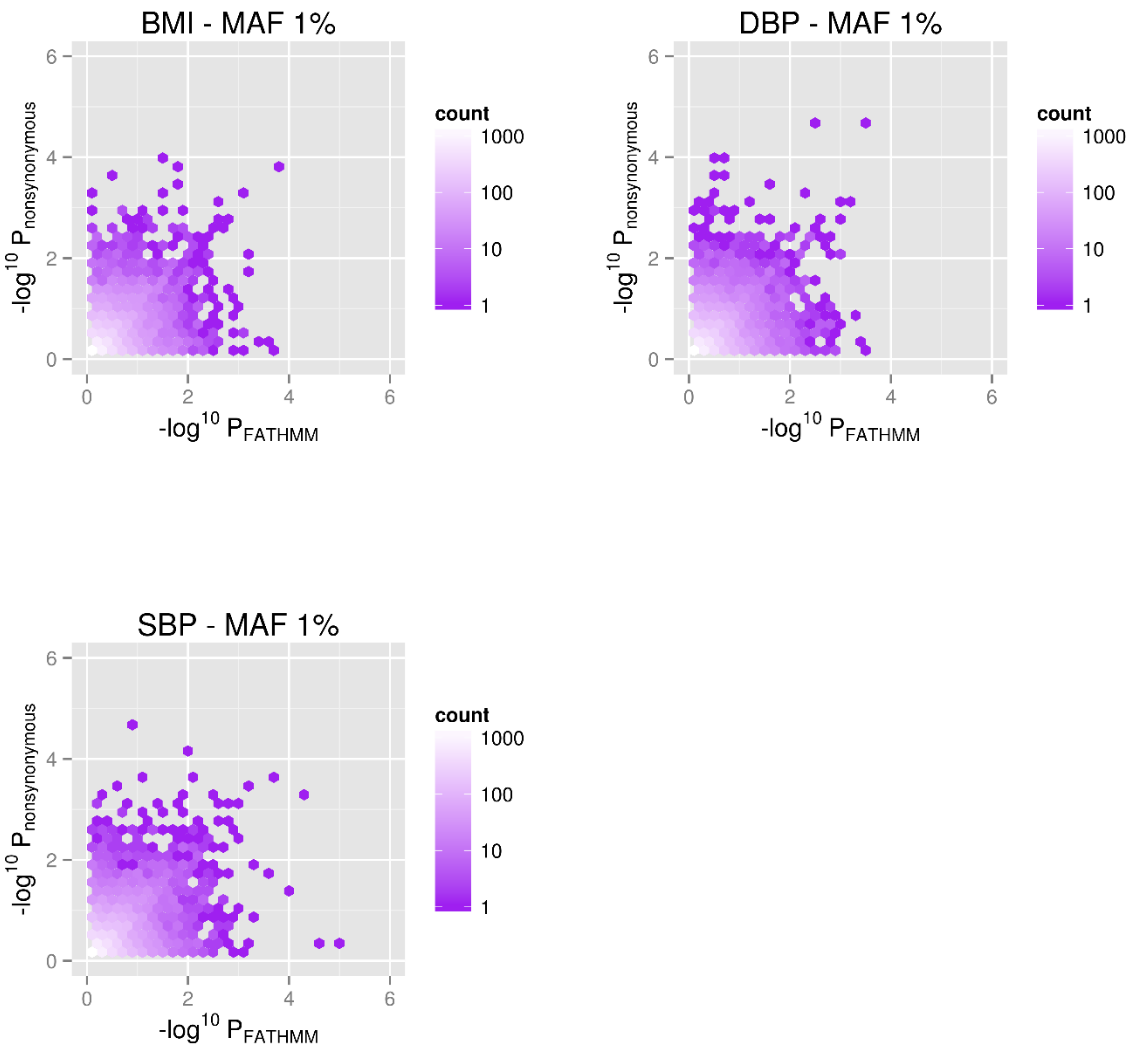

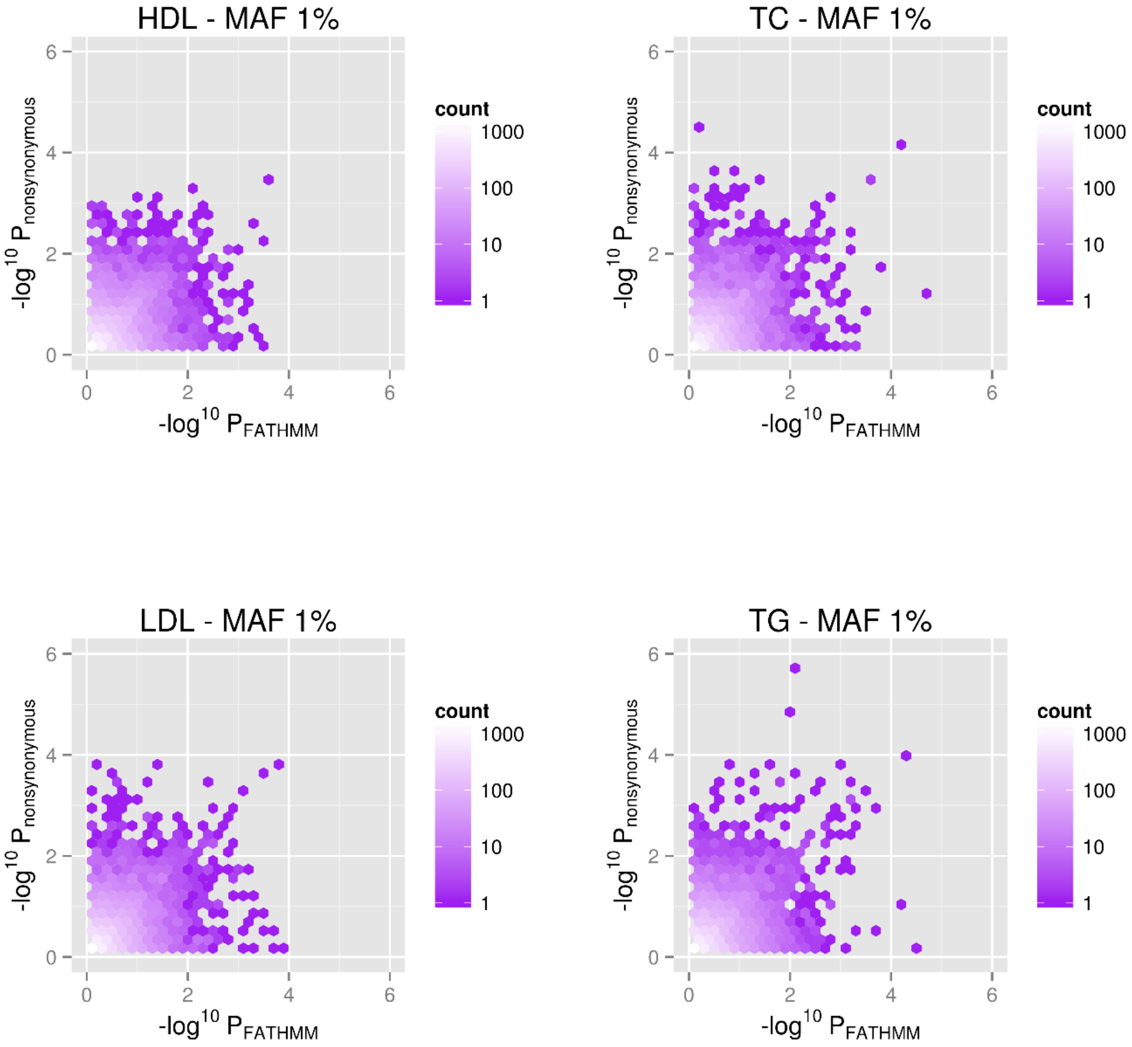
HDL - MAF 1%
TC - MAF 1%
LDL - MAF 1%
TG - MAF 1%
-log10 P nonsynonymous
-log10 P FATHMM
count
1000
100
10
1
0
2
4
6

S15 Figure: Gene-based low frequency variant analyses across the genome using SKAT-O for 7 cardiovascular traits (compared with loss-of-function filtering)

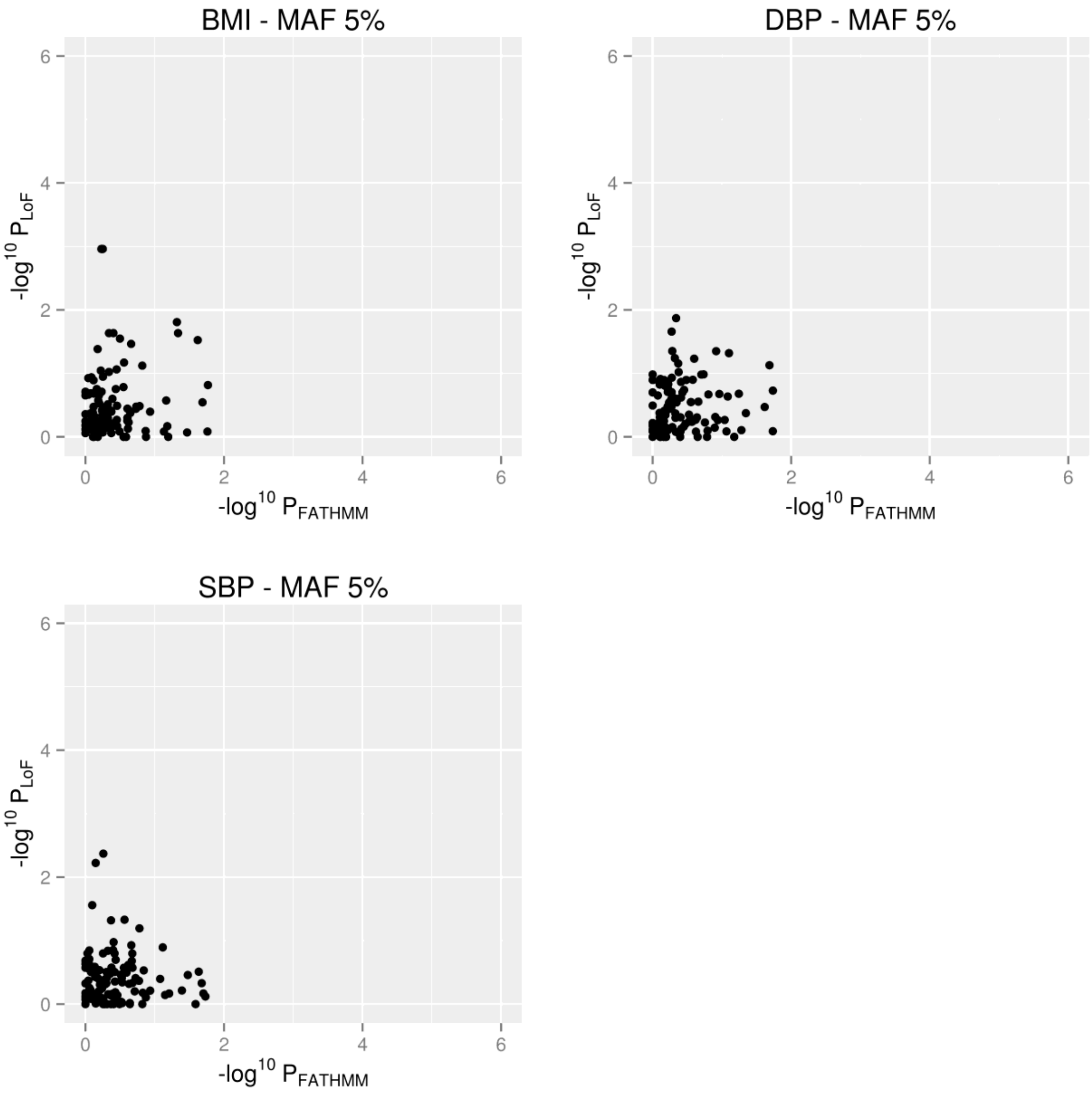

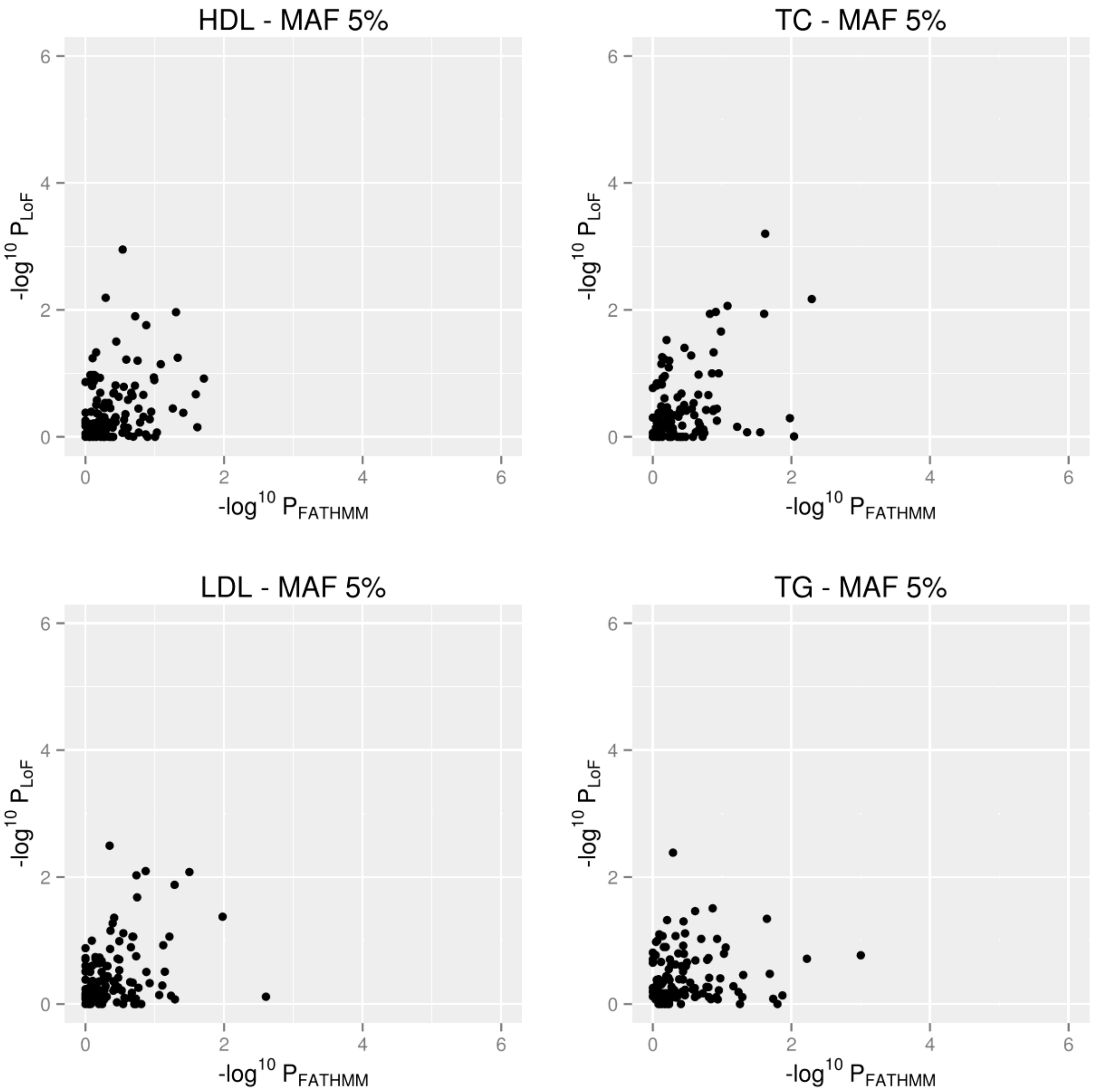

HDL - MAF 5%
TC - MAF 5%
LDL - MAF 5%
TG - MAF 5%
-log10 PLoF
-log10 PFATHMM
0
2
4
6

S16 Figure: Gene-based rare variant analyses across the genome using SKAT-O for 7 cardiovascular traits (compared with loss-of-function filtering)

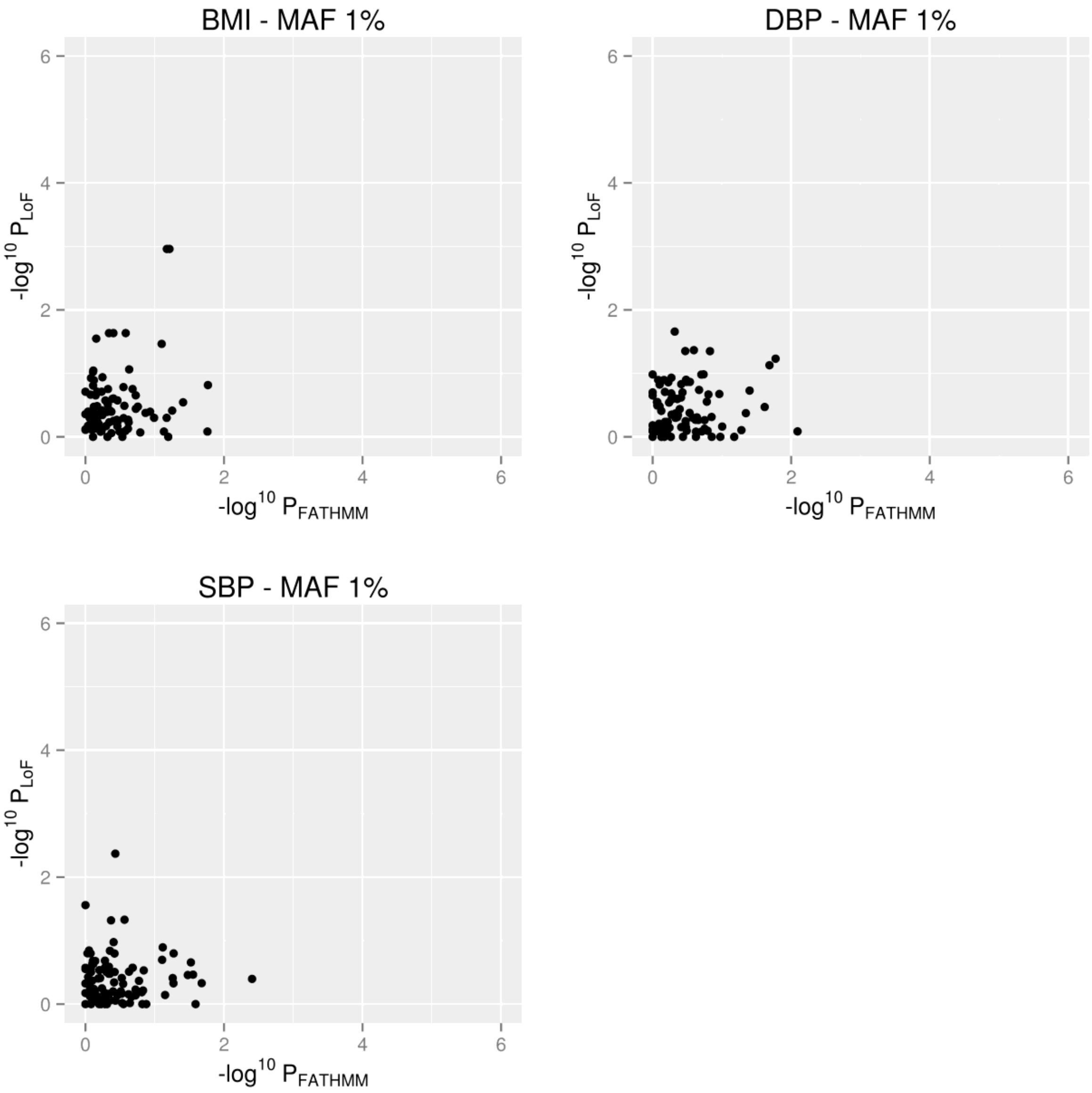

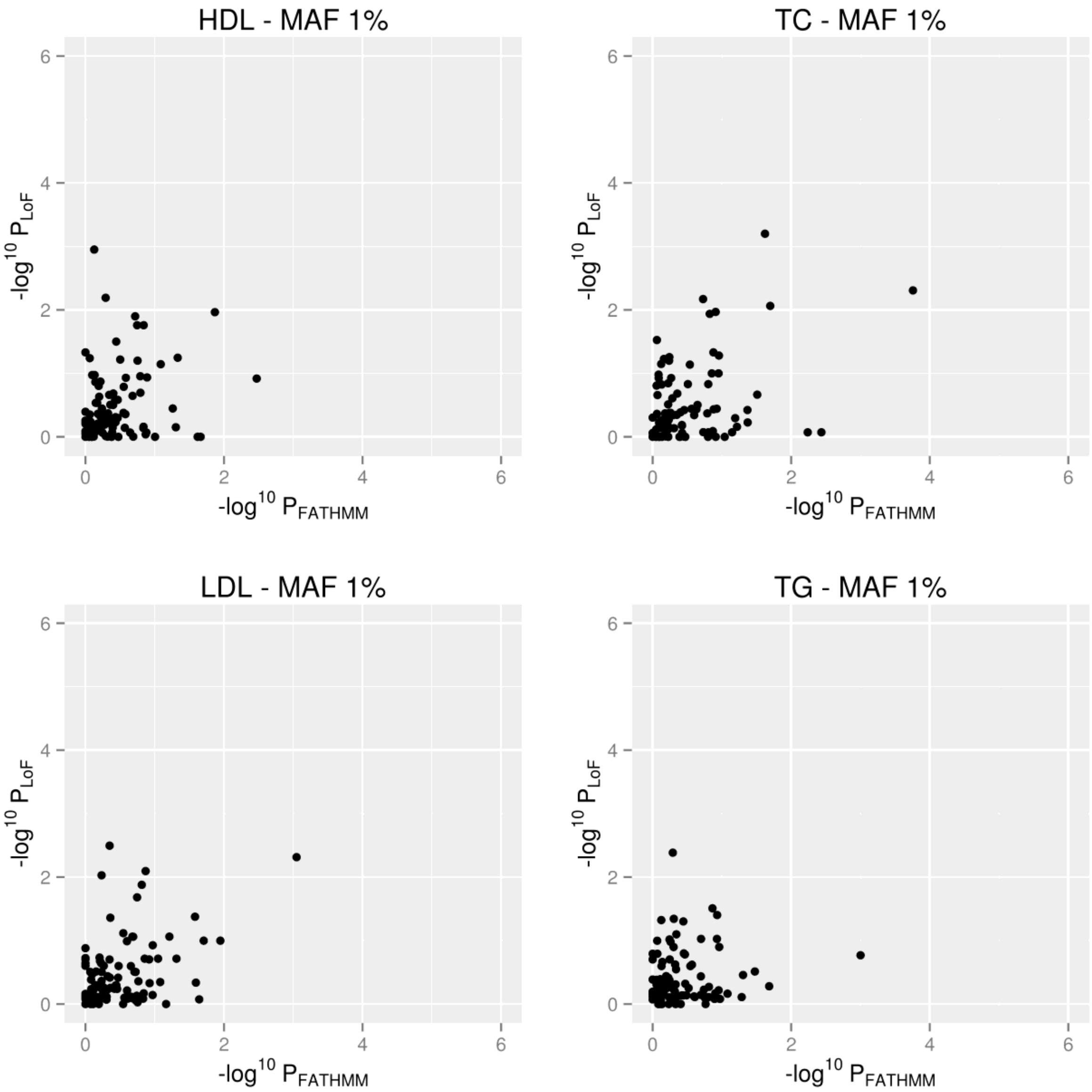

HDL - MAF 1%
TC - MAF 1%
LDL - MAF 1%
TG - MAF 1%
-log10 PLoF
-log10 PFATHMM
0
2
4
6

S17 Figure: Gene-based low frequency variant analyses across the genome using MiST for 7 cardiovascular traits (compared with nonsynonymous filtering)

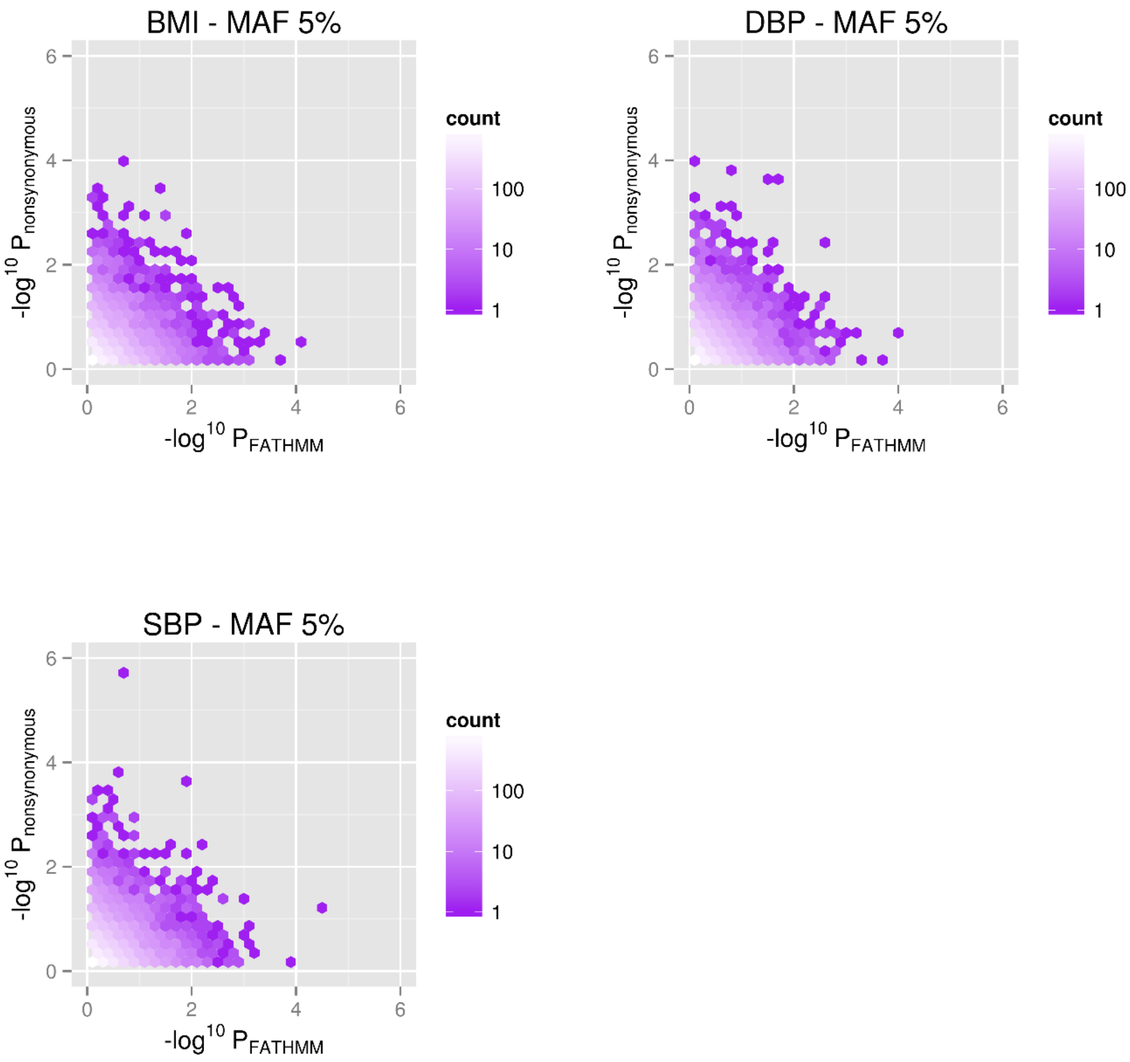

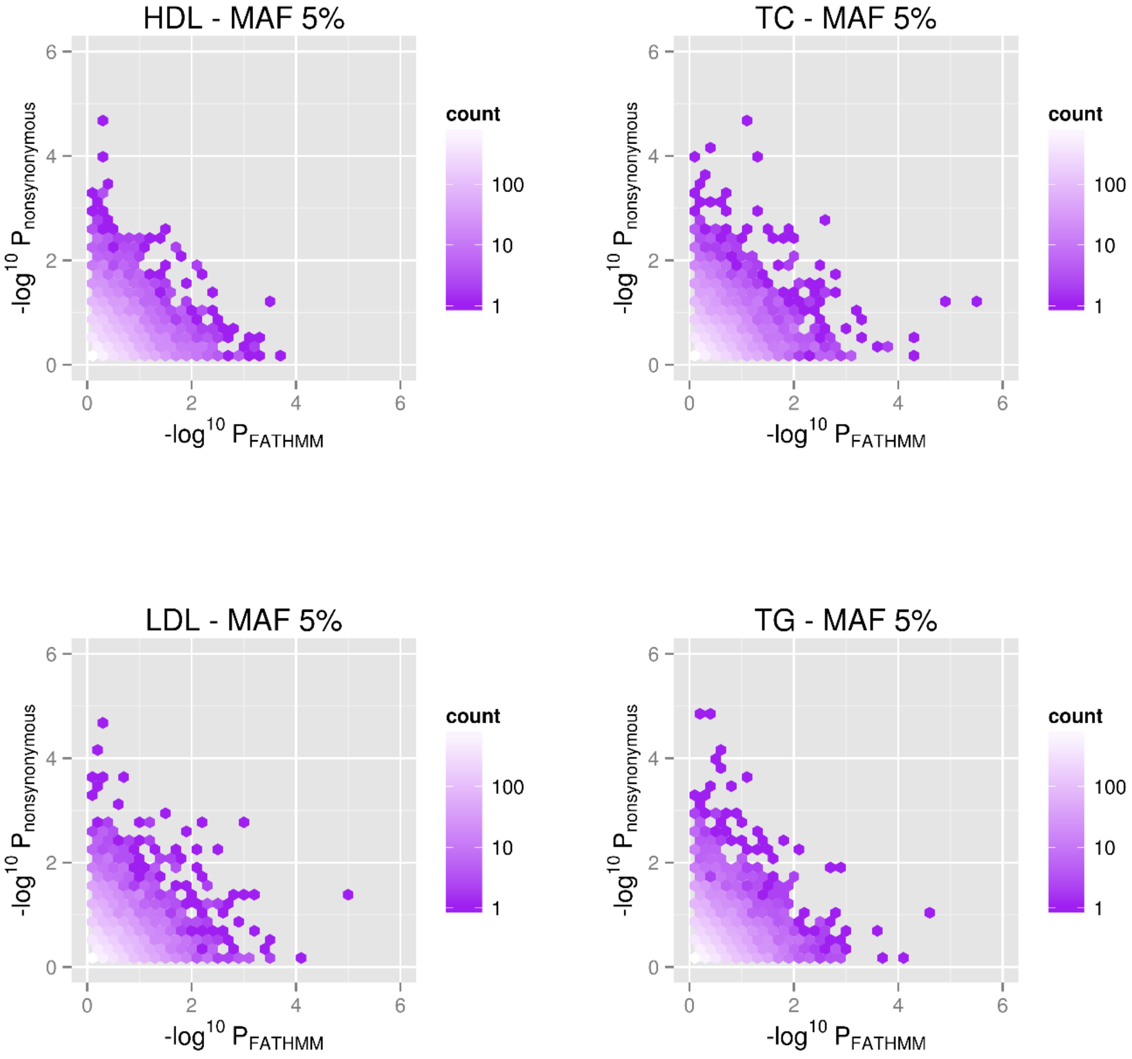

HDL - MAF 5%
TC - MAF 5%
LDL - MAF 5%
TG - MAF 5%
$-\log^{10} P_{nonsynonymous}$
$-\log^{10} P_{FATHMM}$
count
100
10
1
0
2
4
6

S18 Figure: Gene-based rare variant analyses across the genome using MiST for 7 cardiovascular traits (compared with nonsynonymous filtering)

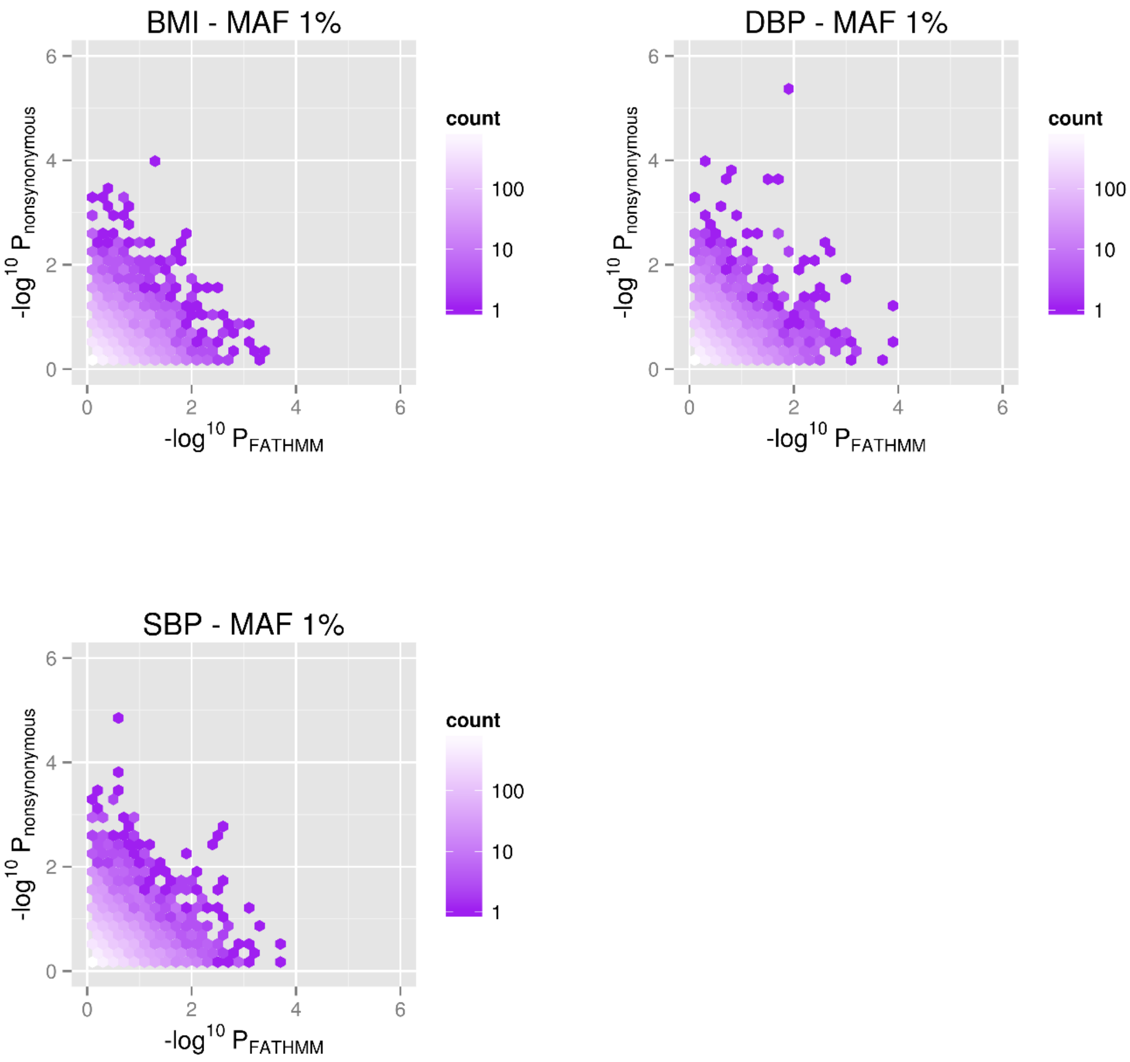

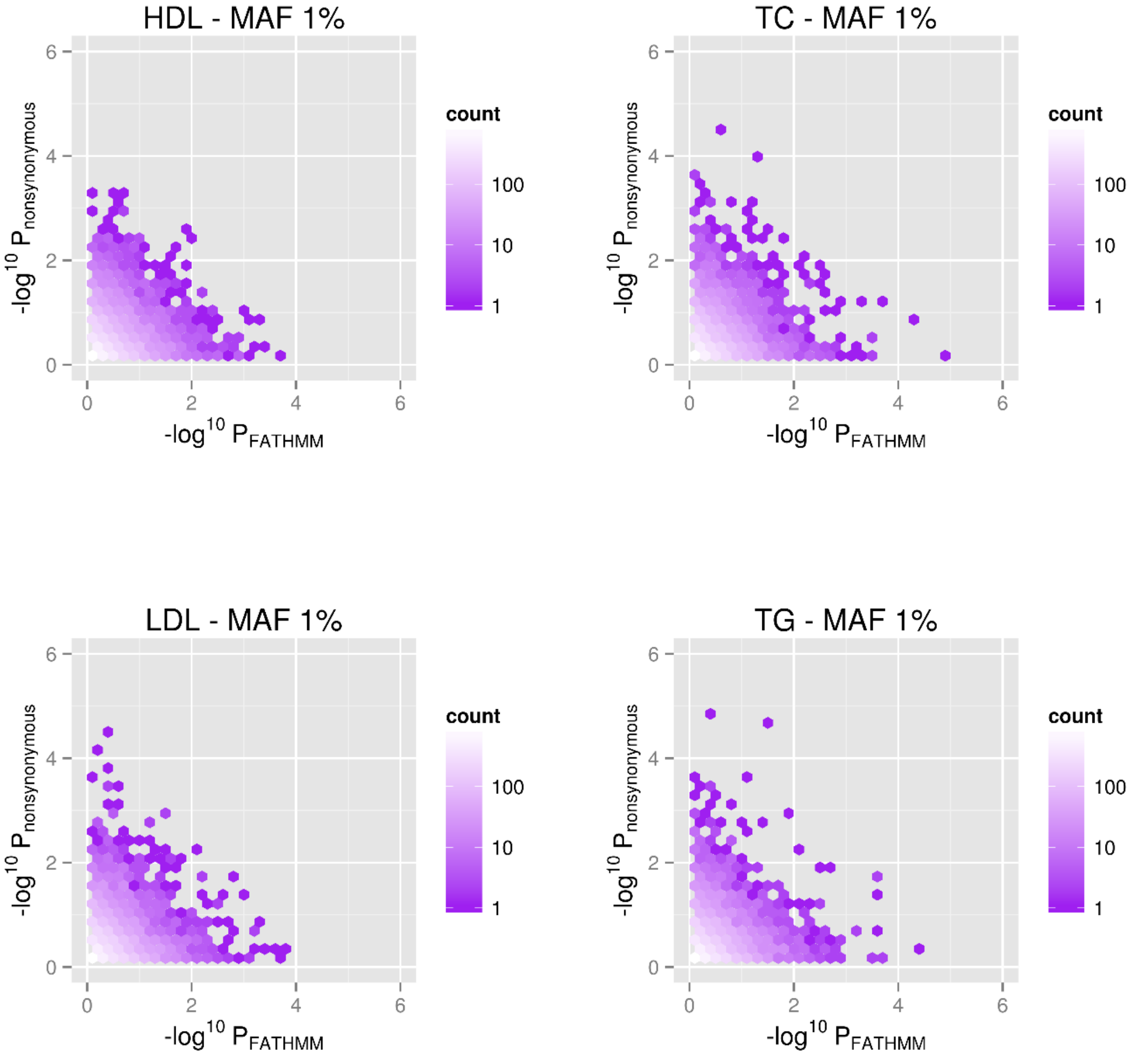
HDL - MAF 1%
TC - MAF 1%
LDL - MAF 1%
TG - MAF 1%
$-\log^{10} P_{nonsynonymous}$
$-\log^{10} P_{FATHMM}$
count
100
10
1
0
2
4
6

S19 Figure: Gene-based low frequency variant analyses across the genome using MiST for 7 cardiovascular traits (compared with loss-of-function filtering)

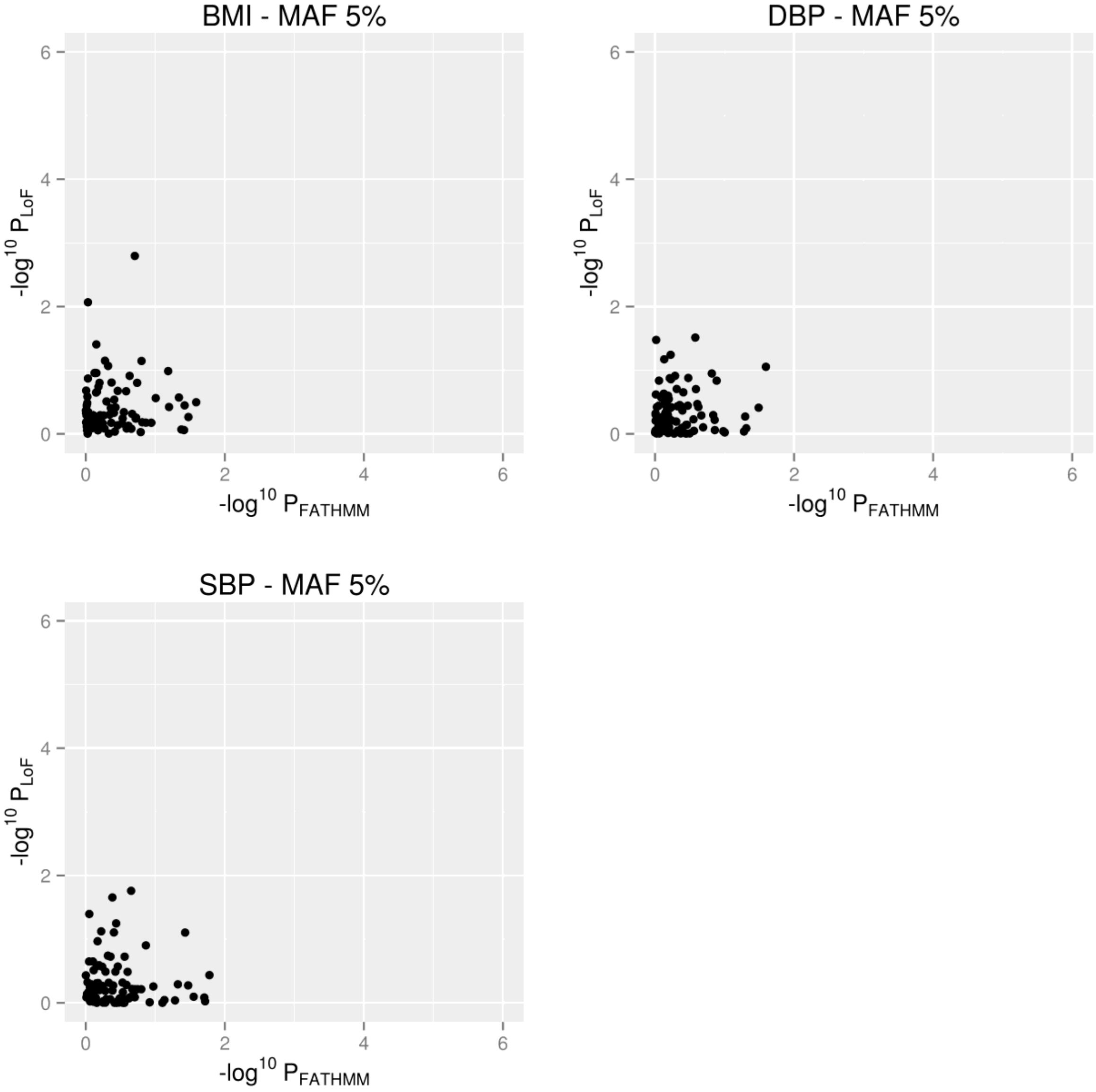

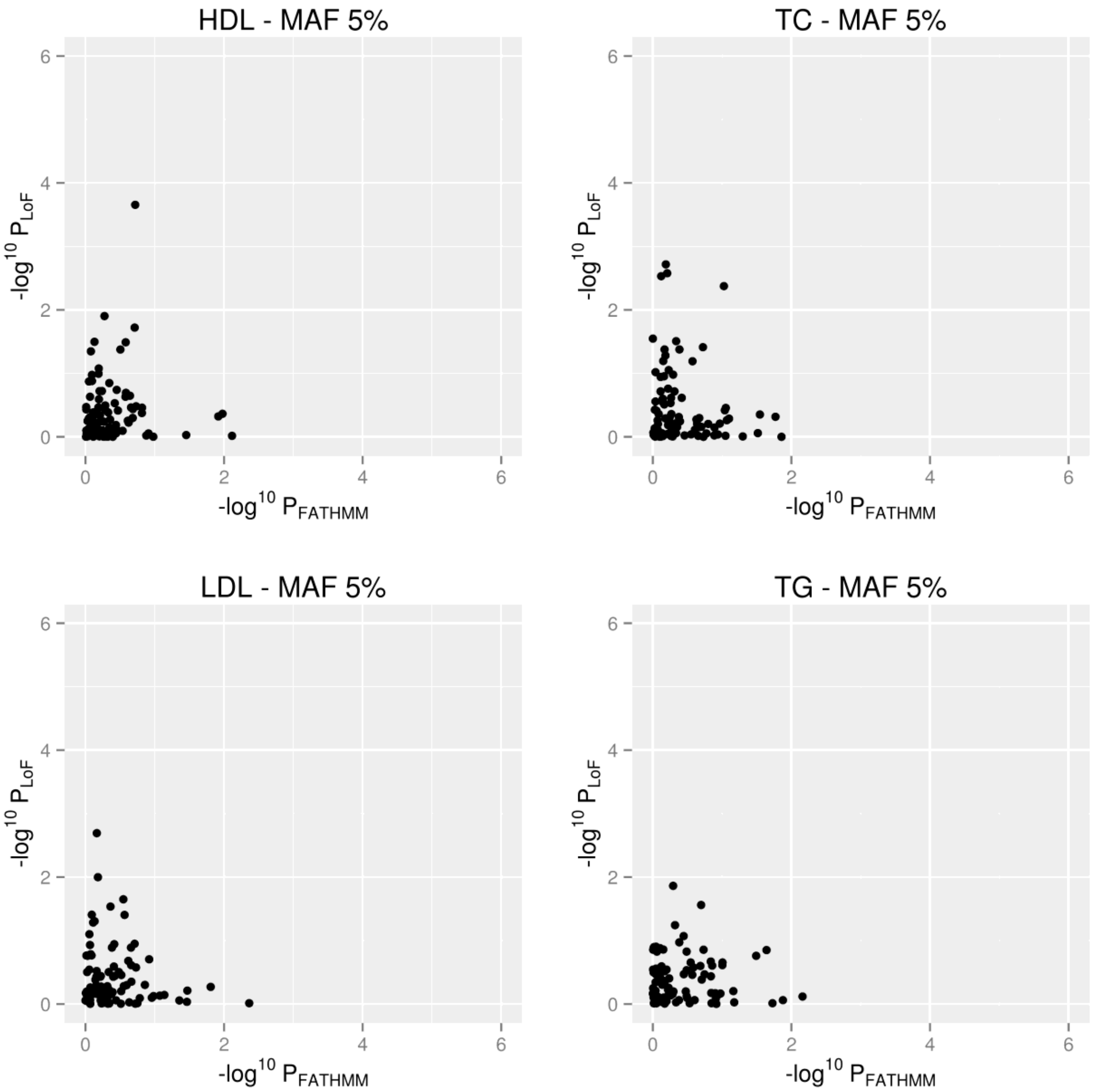

HDL - MAF 5%
TC - MAF 5%
LDL - MAF 5%
TG - MAF 5%
-log10 P_LoF
-log10 P_FATHMM
0
2
4
6

S20 Figure: Gene-based rare variant analyses across the genome using MiST for 7 cardiovascular traits (compared with loss-of-function filtering)

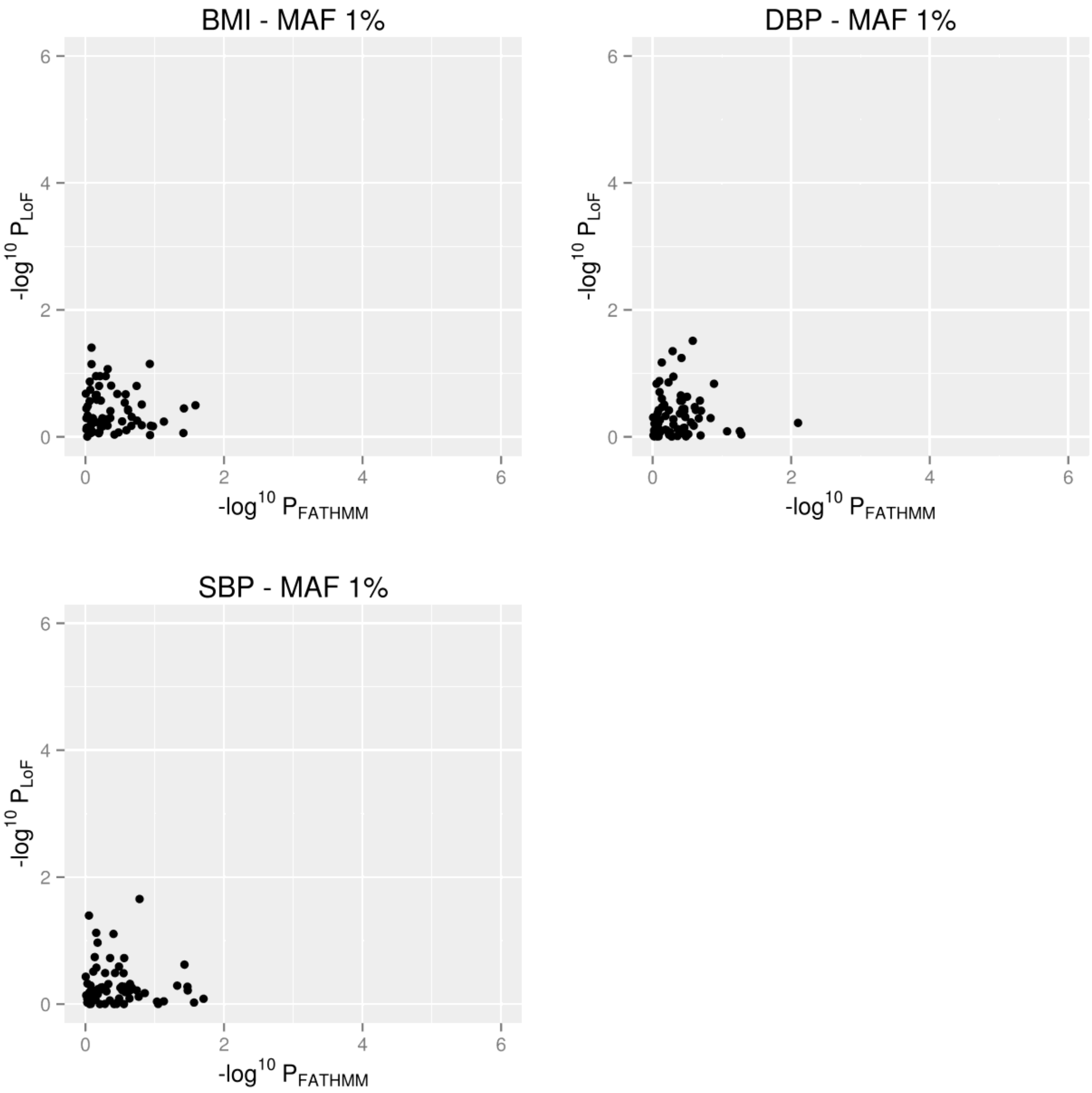

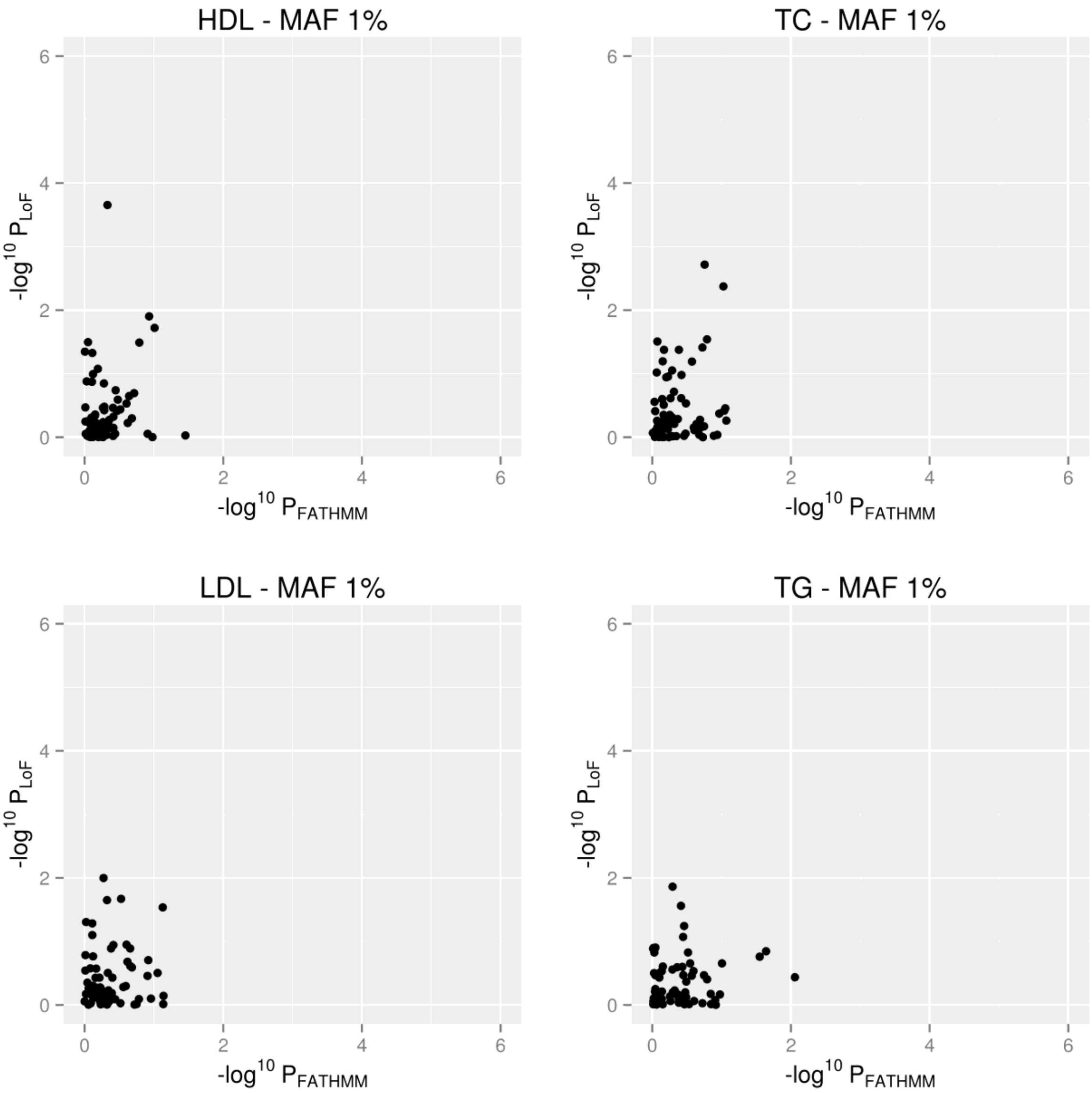
HDL - MAF 1%
TC - MAF 1%
LDL - MAF 1%
TG - MAF 1%
-log10 P_LoF
-log10 P_FATHMM
0
2
4
6

DANN tests

S21 Figure: Gene-based low frequency variant analyses across the genome using SKAT-O for 7 cardiovascular traits (compared with nonsynonymous filtering)

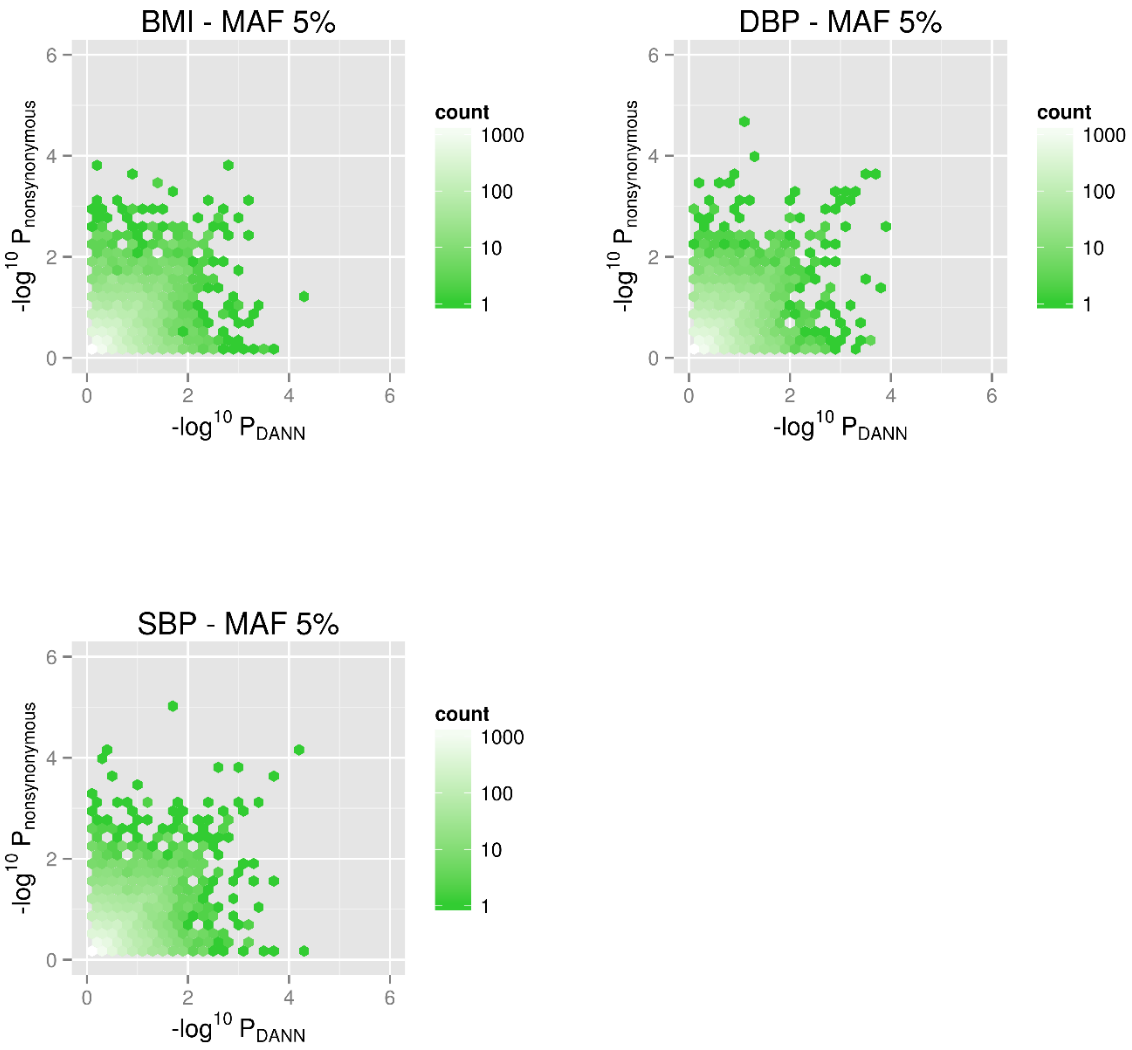

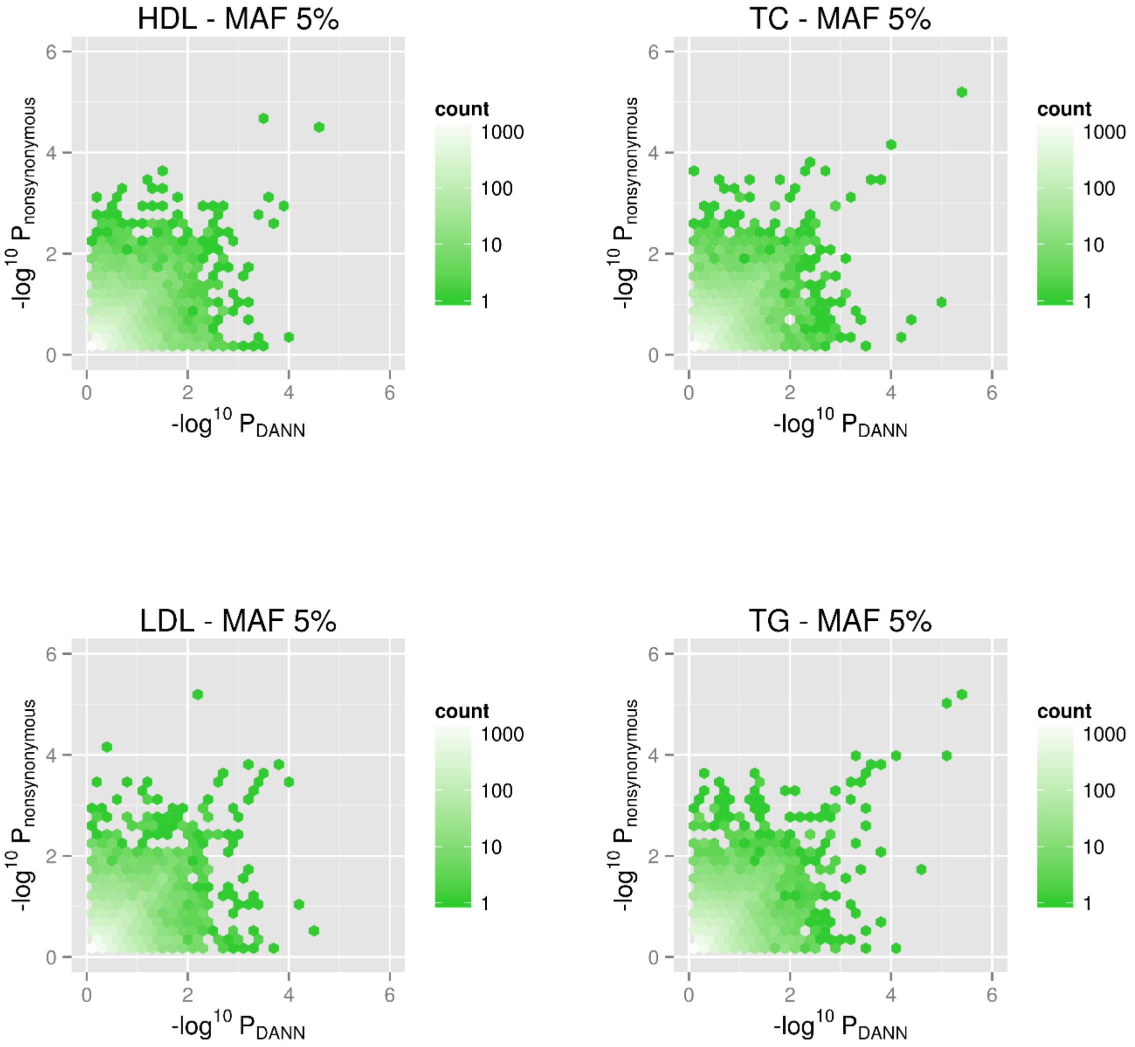
HDL - MAF 5%
TC - MAF 5%
LDL - MAF 5%
TG - MAF 5%
-log10 P nonsynonymous
-log10 P DANN
count
1000
100
10
1
0
2
4
6

S22 Figure: Gene-based rare variant analyses across the genome using SKAT-O for 7 cardiovascular traits (compared with nonsynonymous filtering)

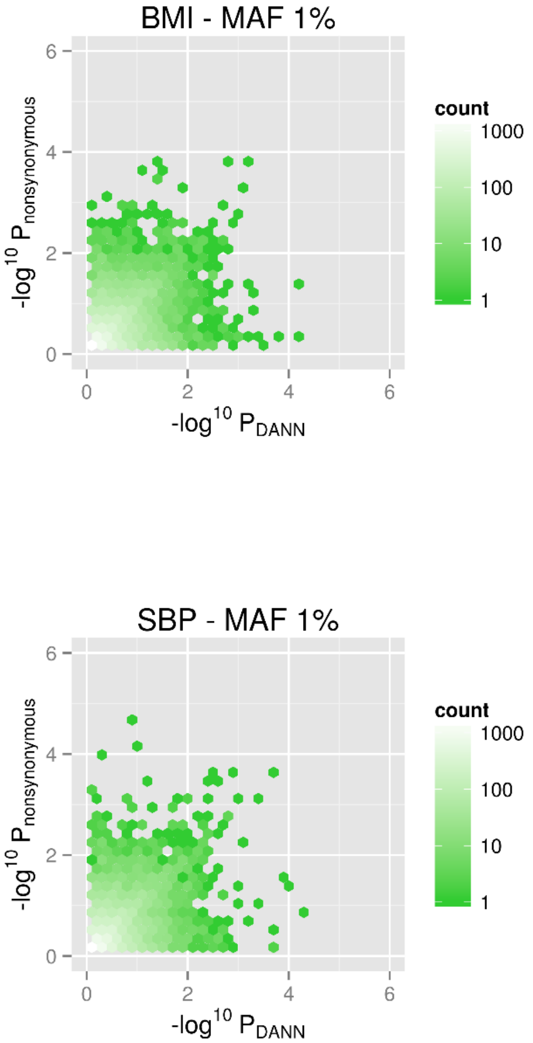

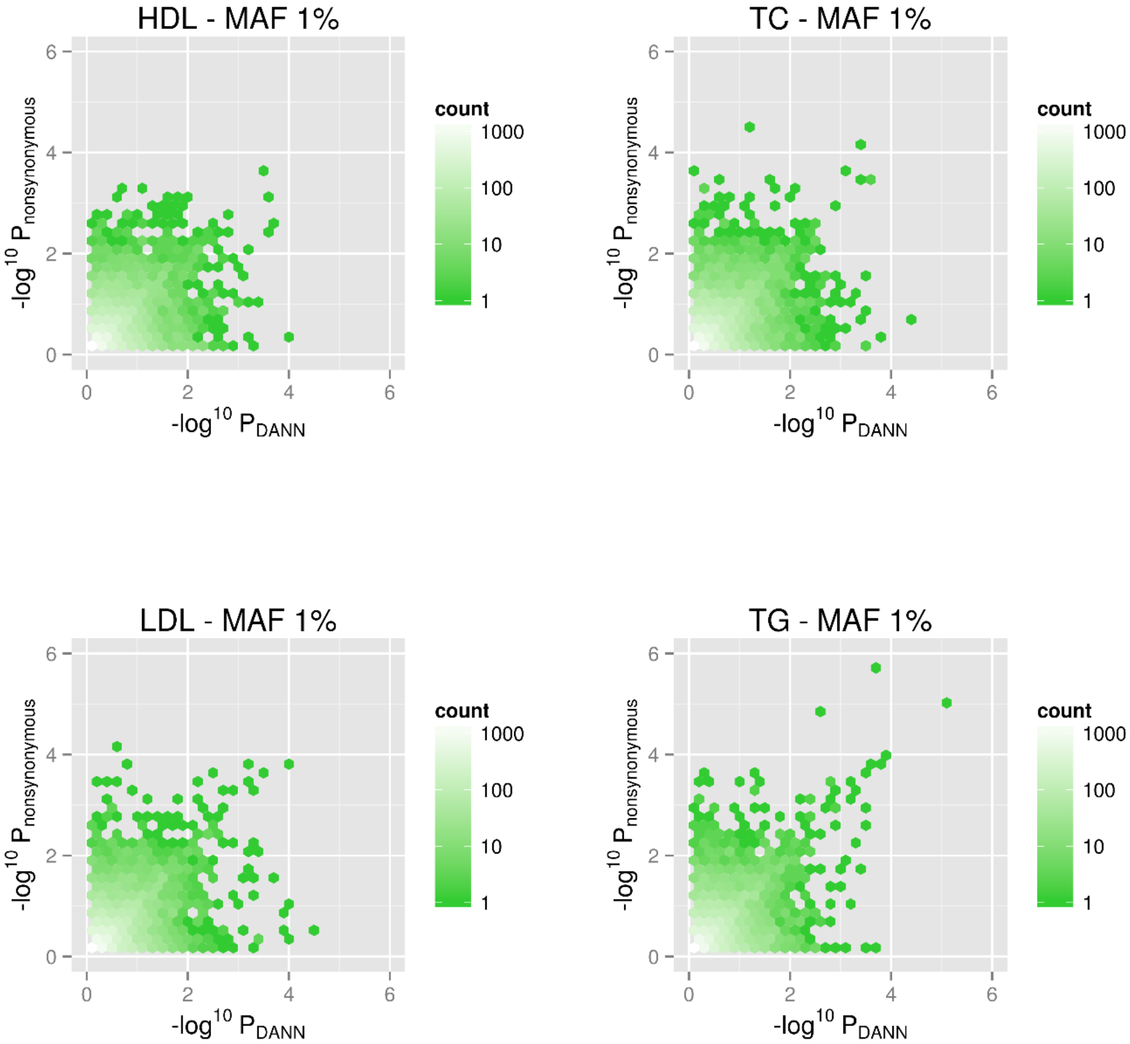
HDL - MAF 1%
TC - MAF 1%
LDL - MAF 1%
TG - MAF 1%
-log10 P_nonsynonymous
-log10 P_DANN
0
2
4
6
count
1000
100
10
1

S23 Figure: Gene-based low frequency variant analyses across the genome using SKAT-O for 7 cardiovascular traits (compared with loss-of-function filtering)

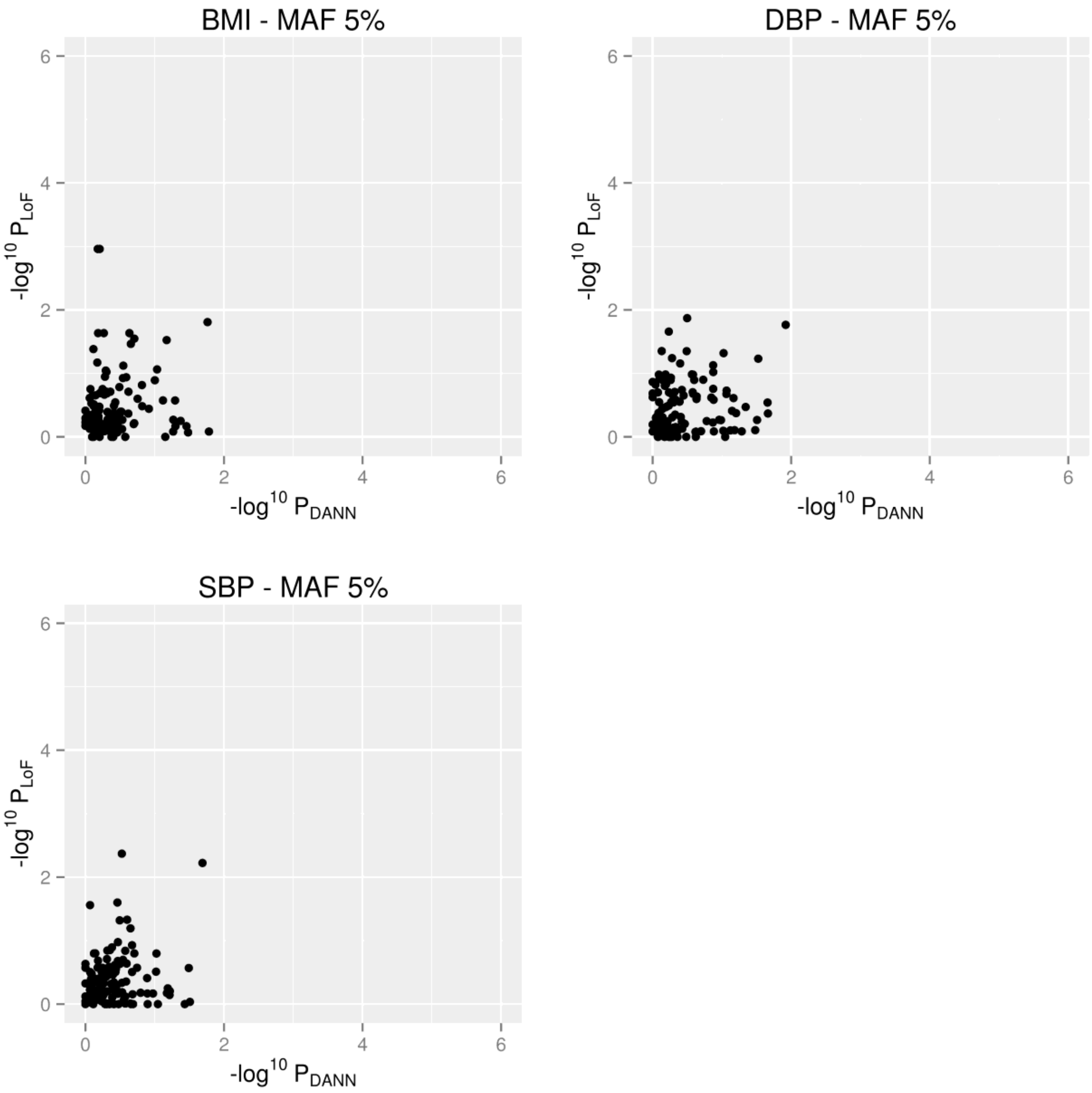

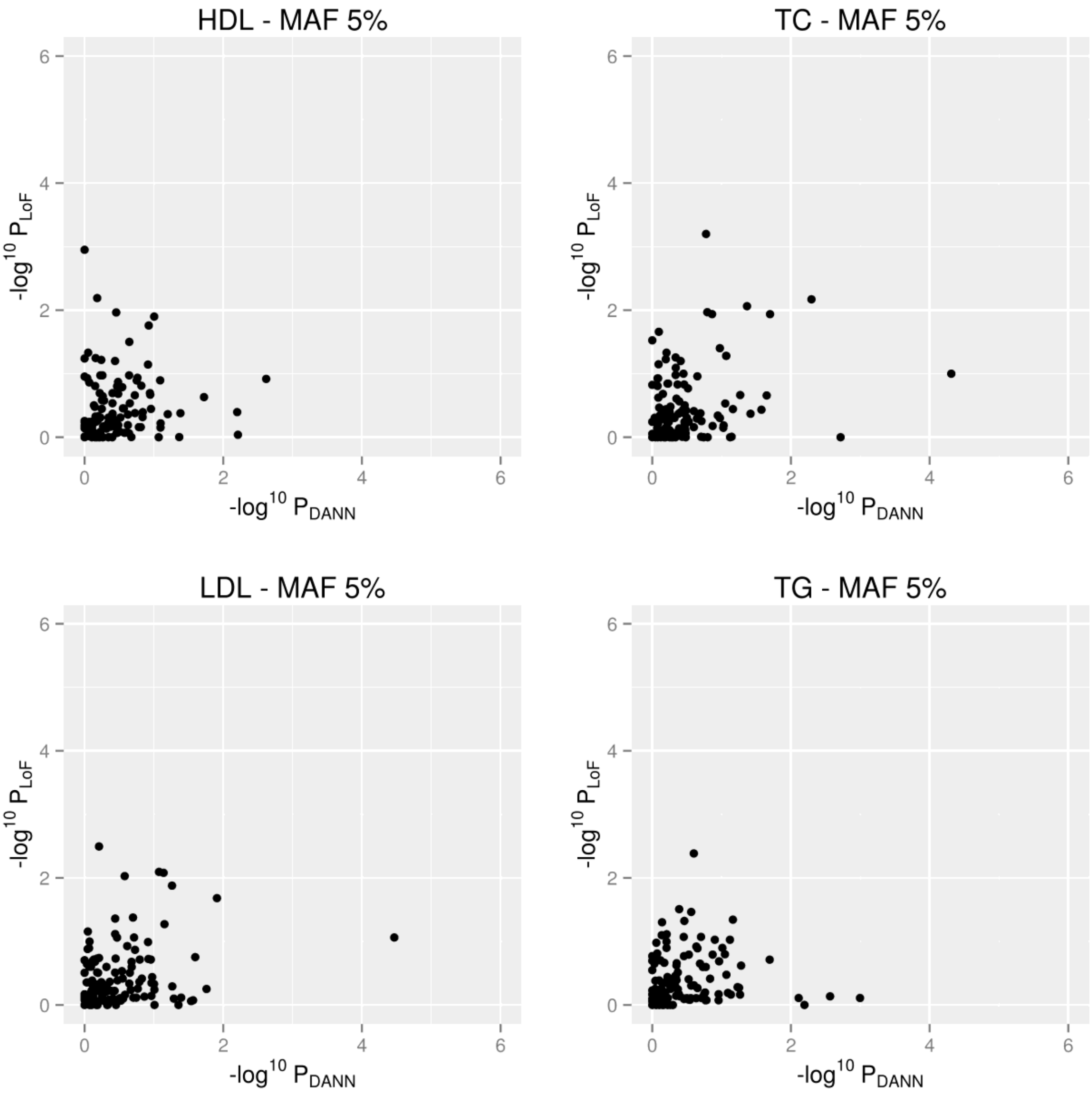

HDL - MAF 5%
TC - MAF 5%
LDL - MAF 5%
TG - MAF 5%
-log10 P_LoF
-log10 P_DANN
0
2
4
6

S24 Figure: Gene-based rare variant analyses across the genome using SKAT-O for 7 cardiovascular traits (compared with loss-of-function filtering)

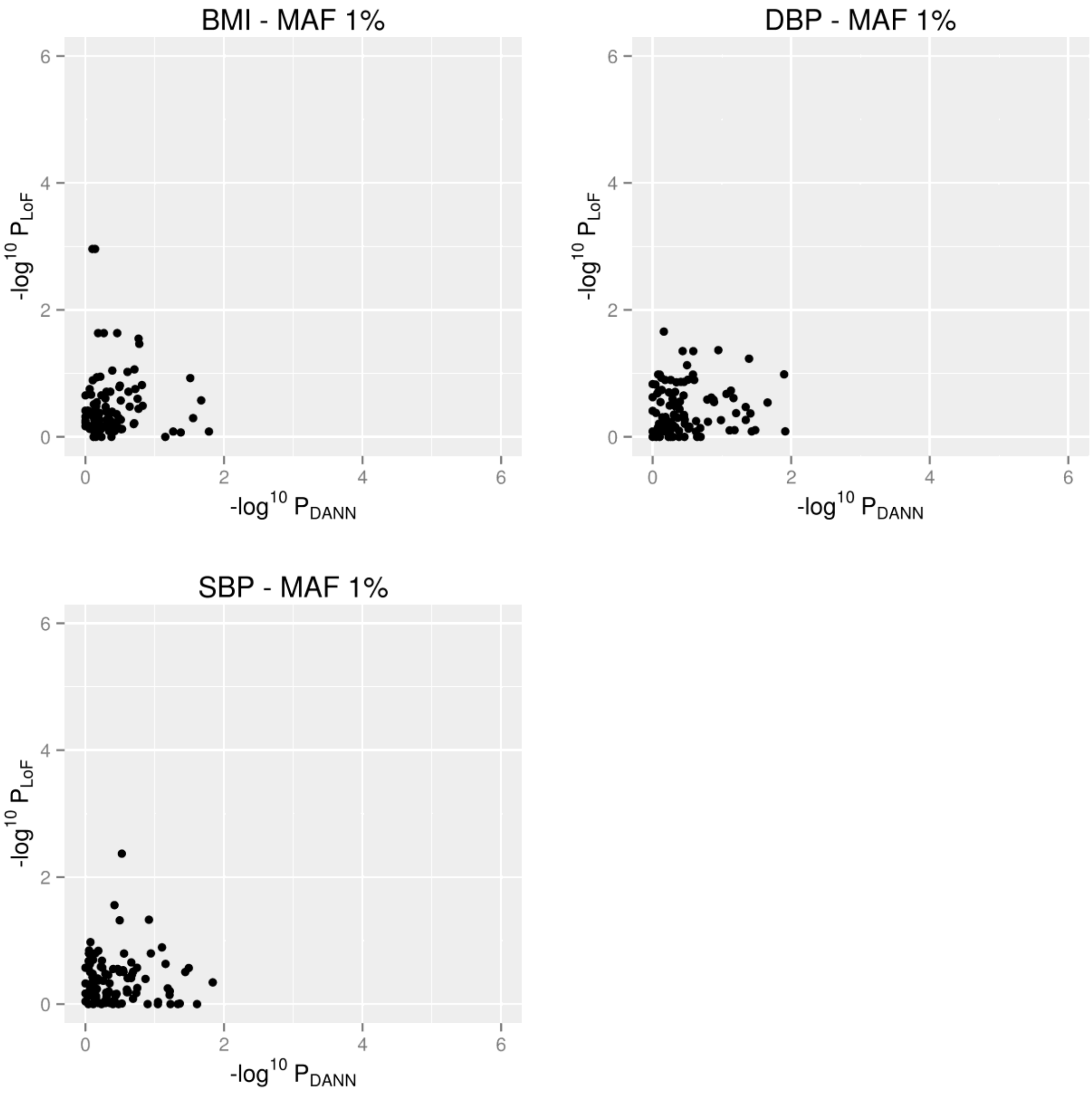

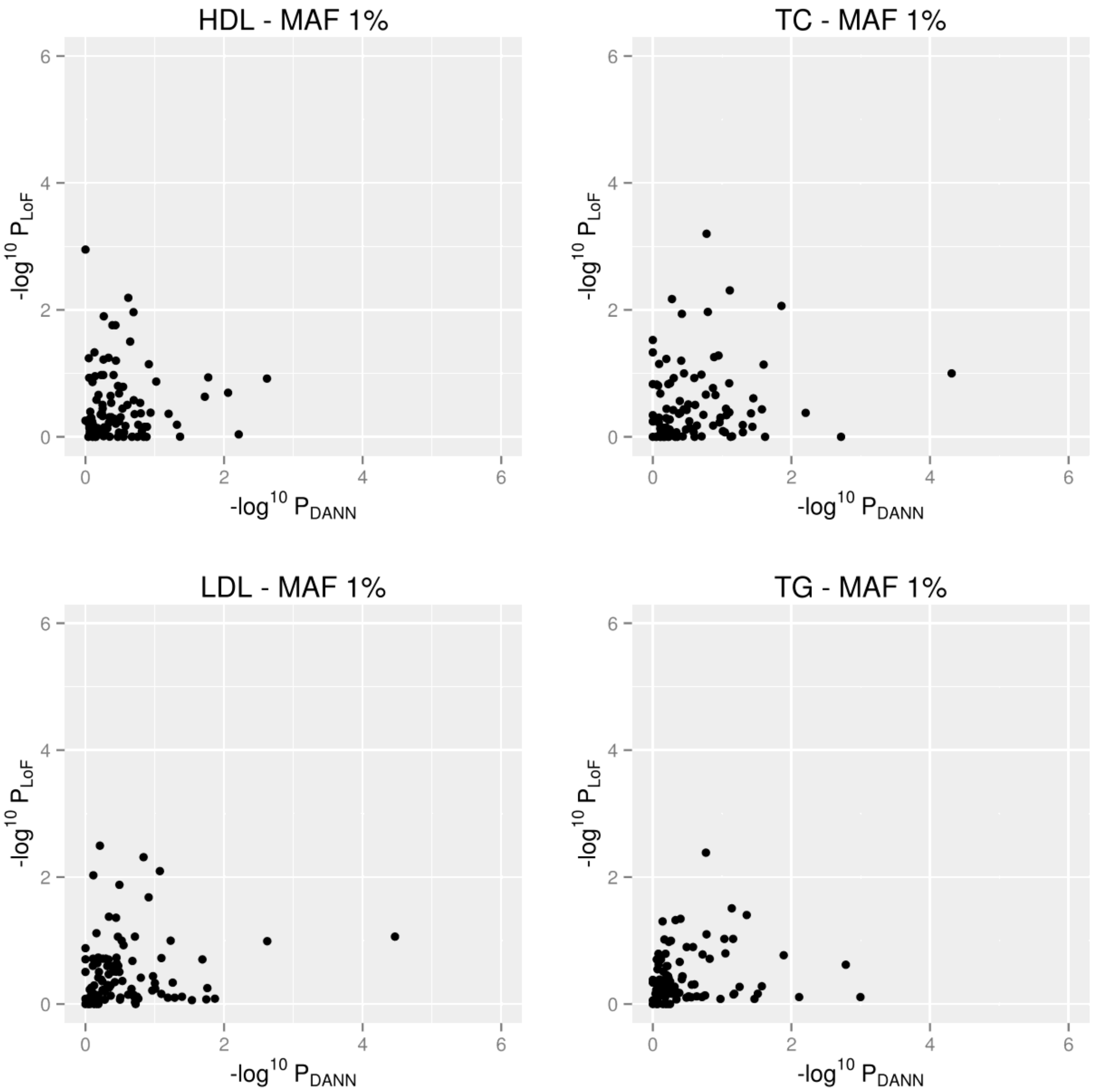

HDL - MAF 1%
TC - MAF 1%
LDL - MAF 1%
TG - MAF 1%
$-\log^{10} P_{LoF}$
$-\log^{10} P_{DANN}$
0
2
4
6

S25 Figure: Gene-based low frequency variant analyses across the genome using MiST for 7 cardiovascular traits (compared with nonsynonymous filtering)

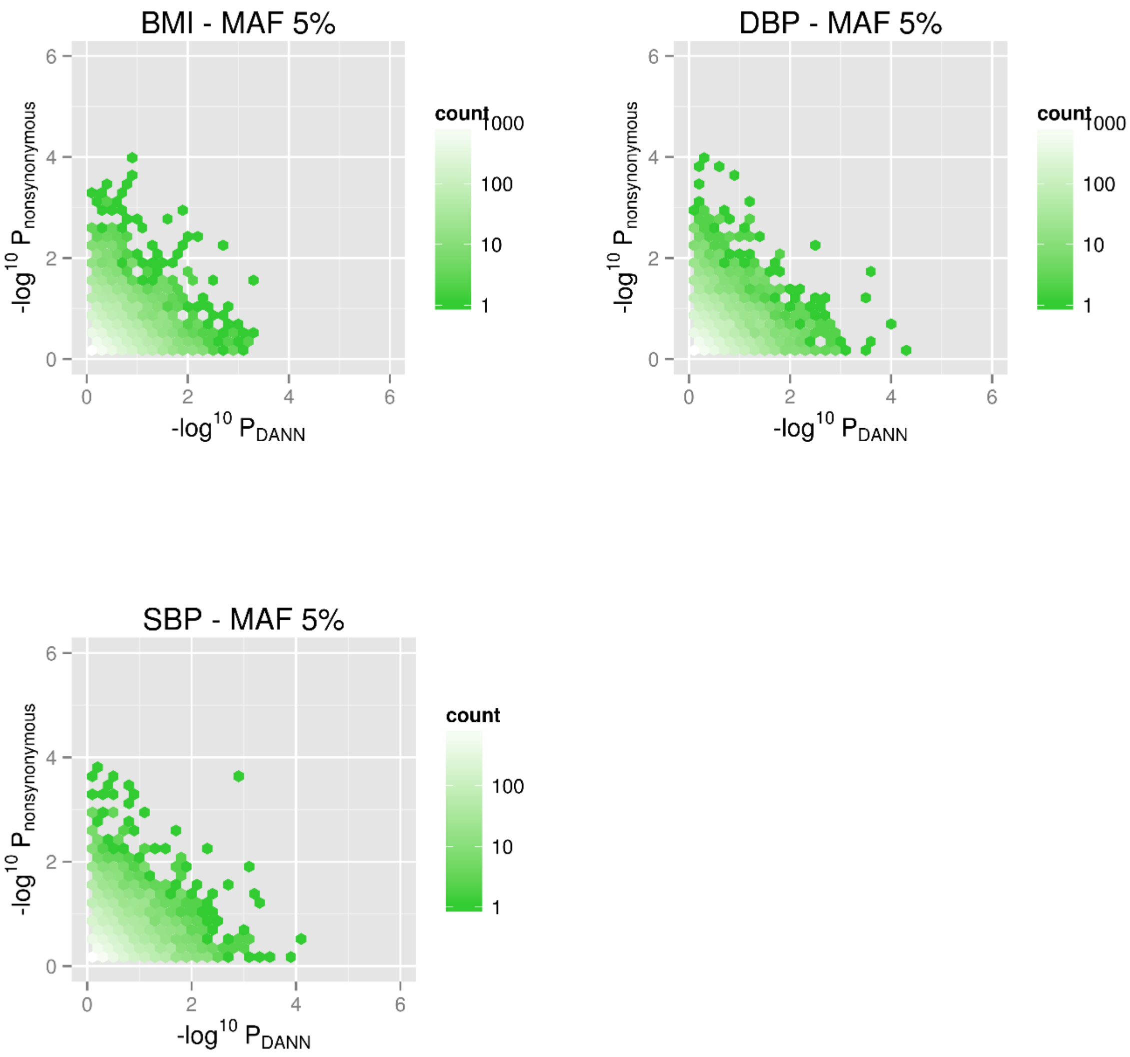

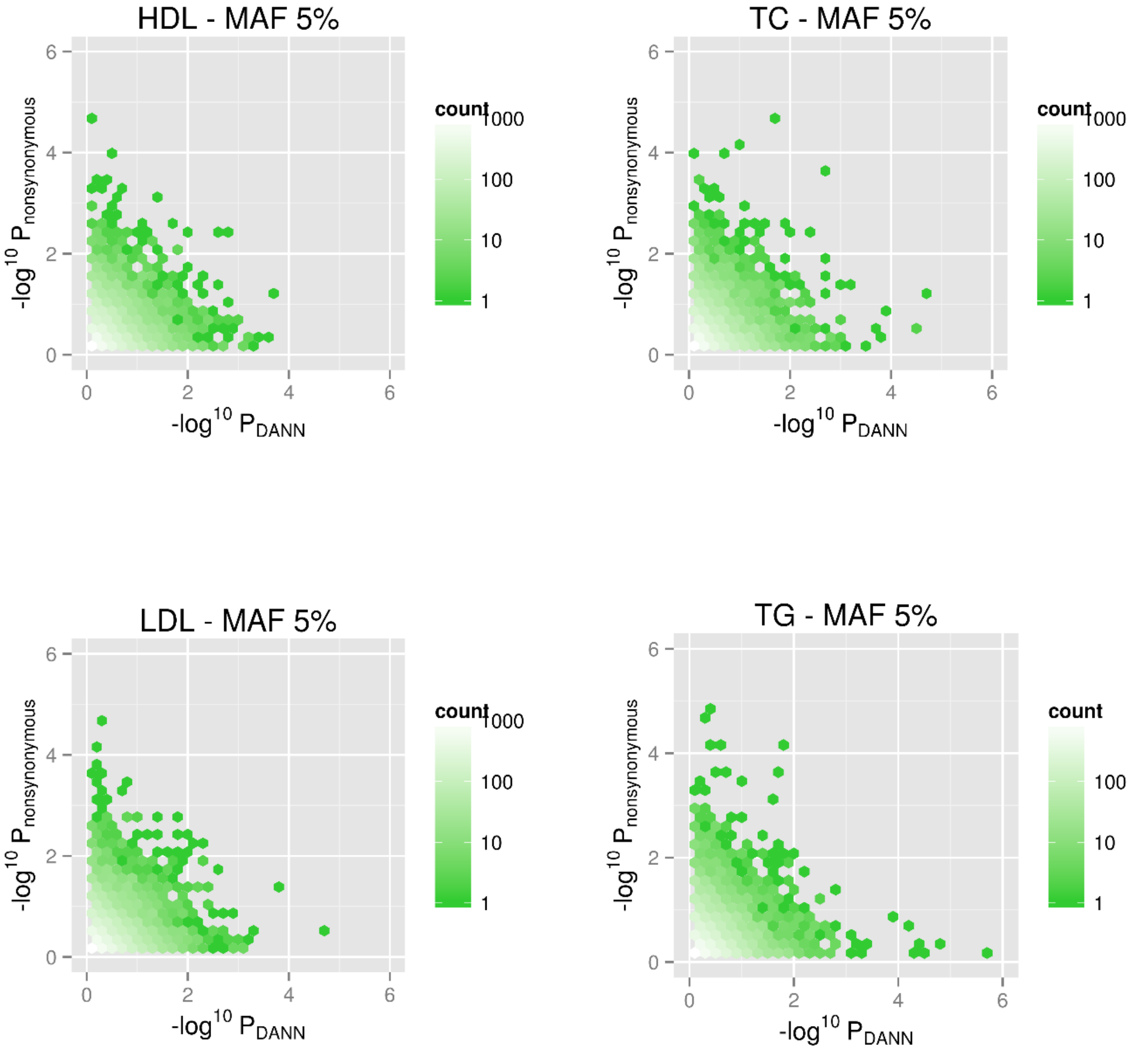

HDL - MAF 5%
TC - MAF 5%
LDL - MAF 5%
TG - MAF 5%
-log10 P nonsynonymous
-log10 P DANN
count
1000
100
10
1
0
2
4
6

S26 Figure: Gene-based rare variant analyses across the genome using MiST for 7 cardiovascular traits (compared with nonsynonymous filtering)

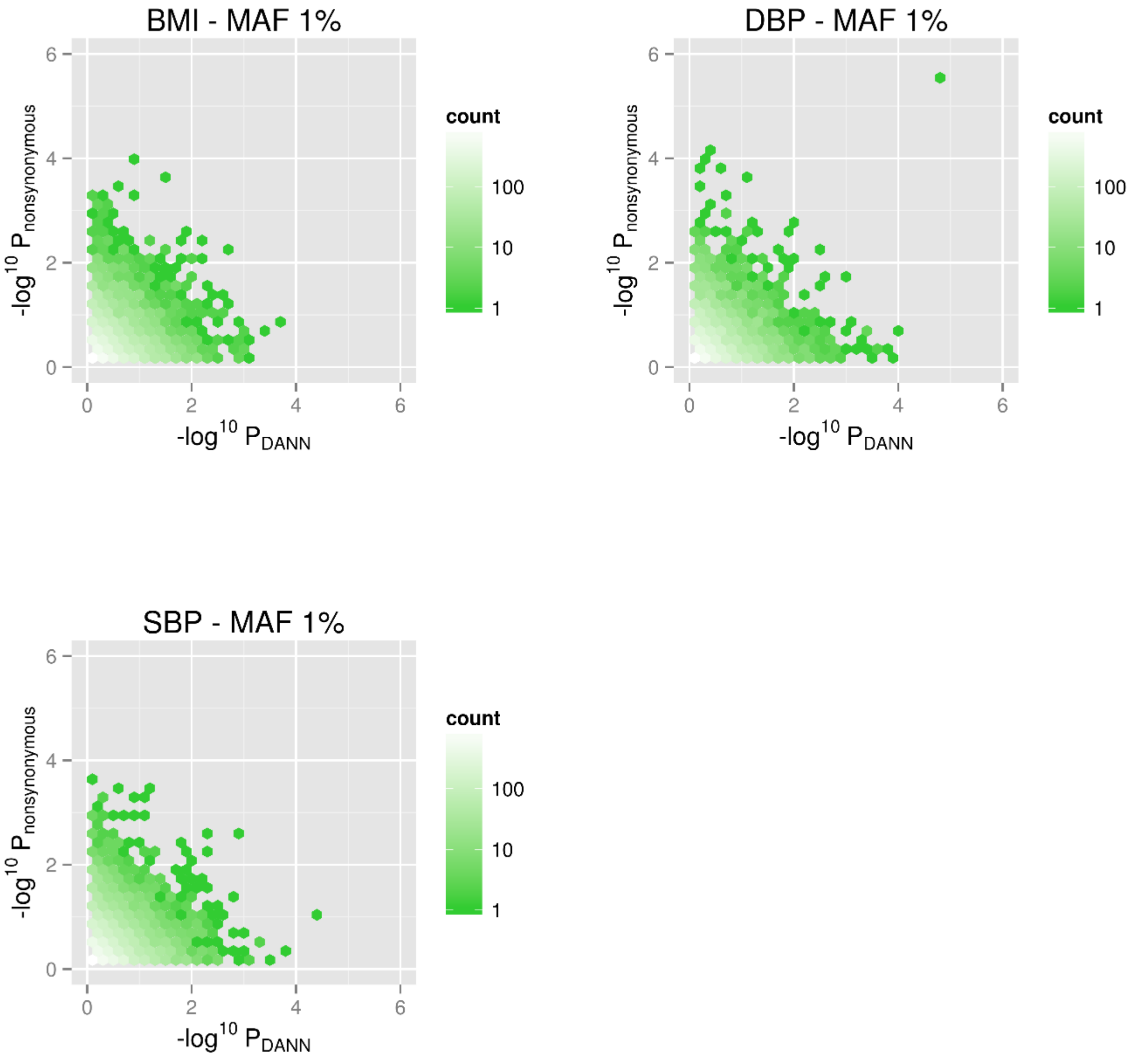

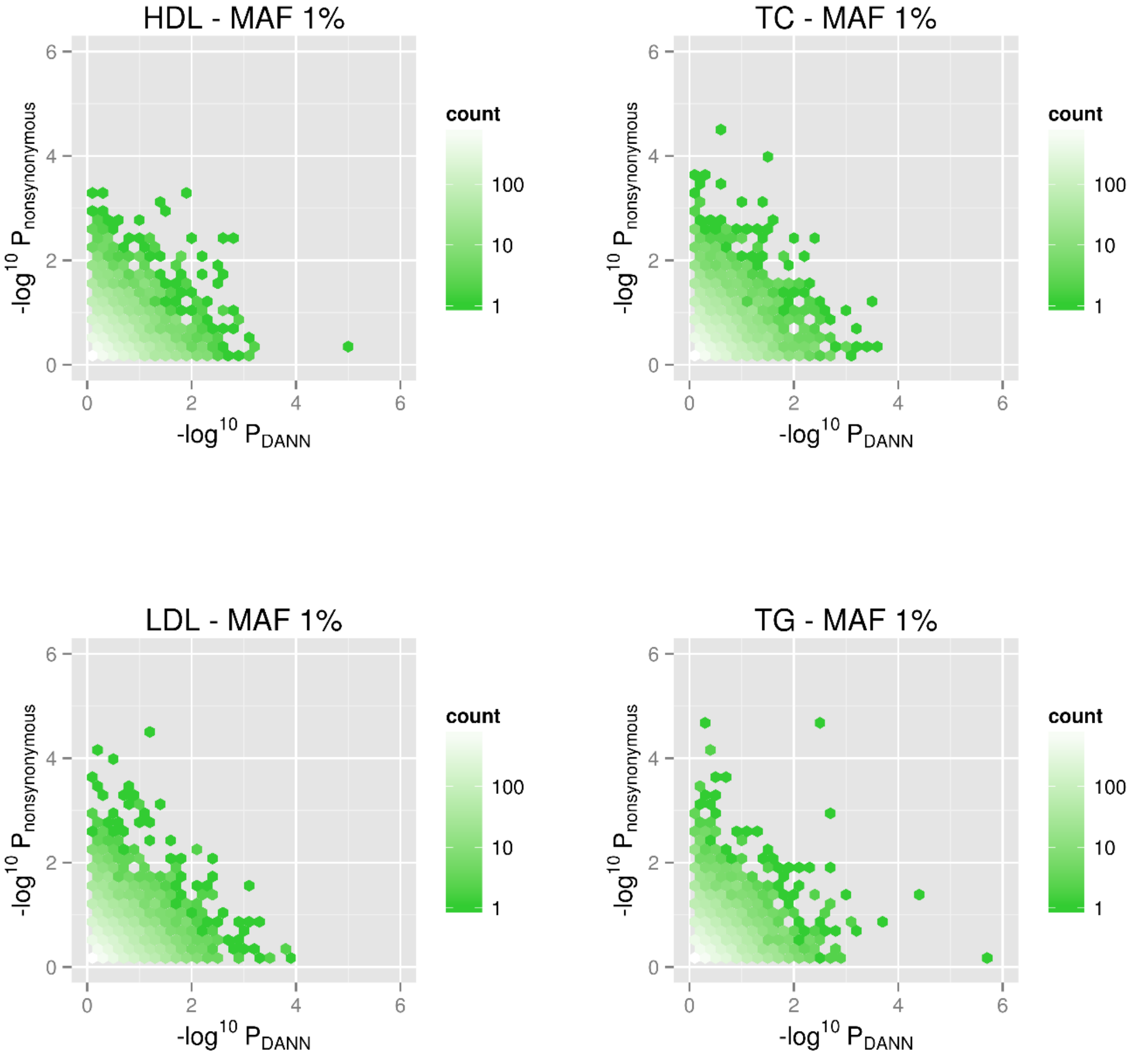

HDL - MAF 1%
TC - MAF 1%
LDL - MAF 1%
TG - MAF 1%
-log10 P nonsynonymous
-log10 P DANN
count
100
10
1
0
2
4
6

S27 Figure: Gene-based low frequency variant analyses across the genome using MiST for 7 cardiovascular traits (compared with loss-of-function filtering)

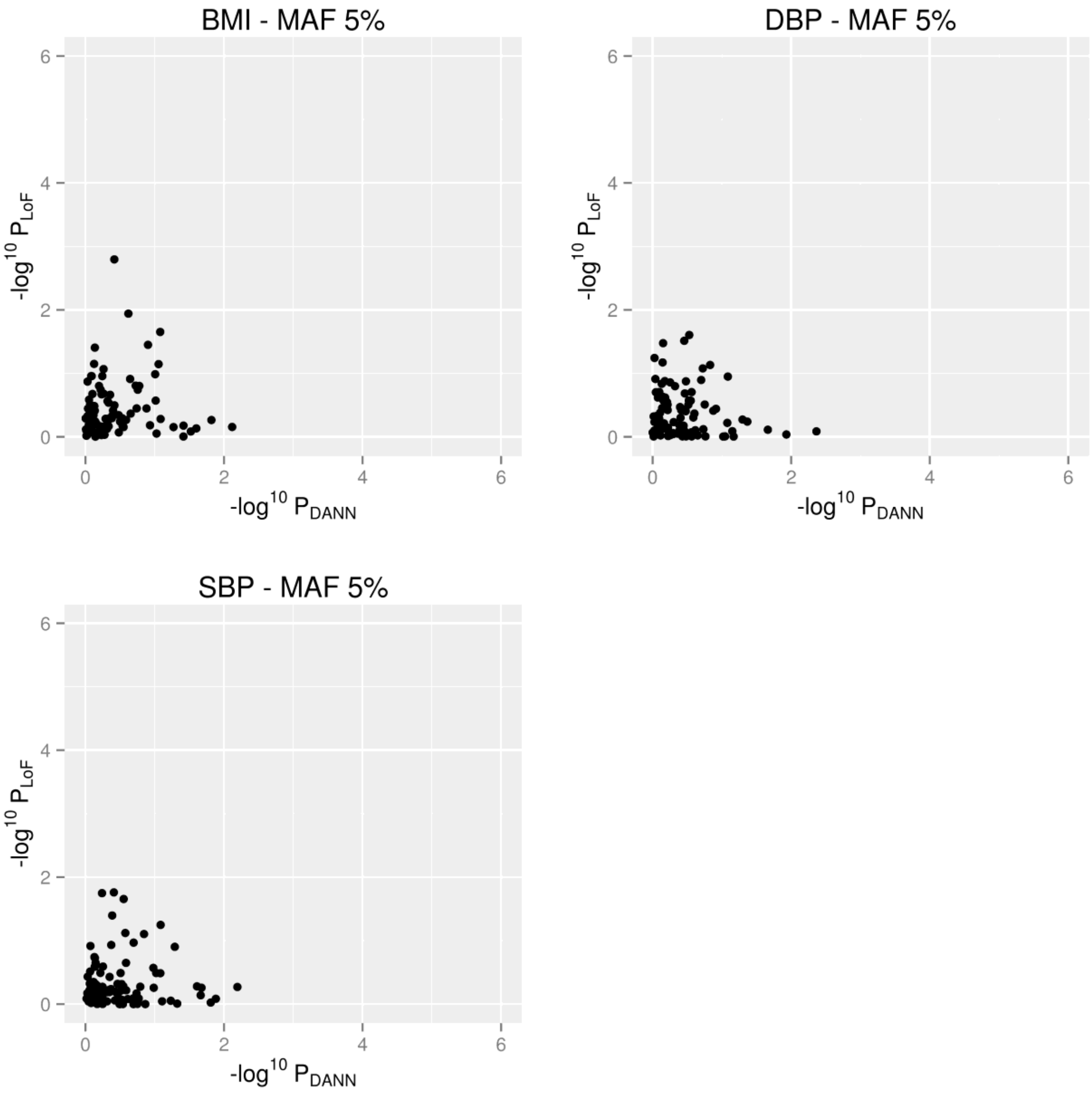

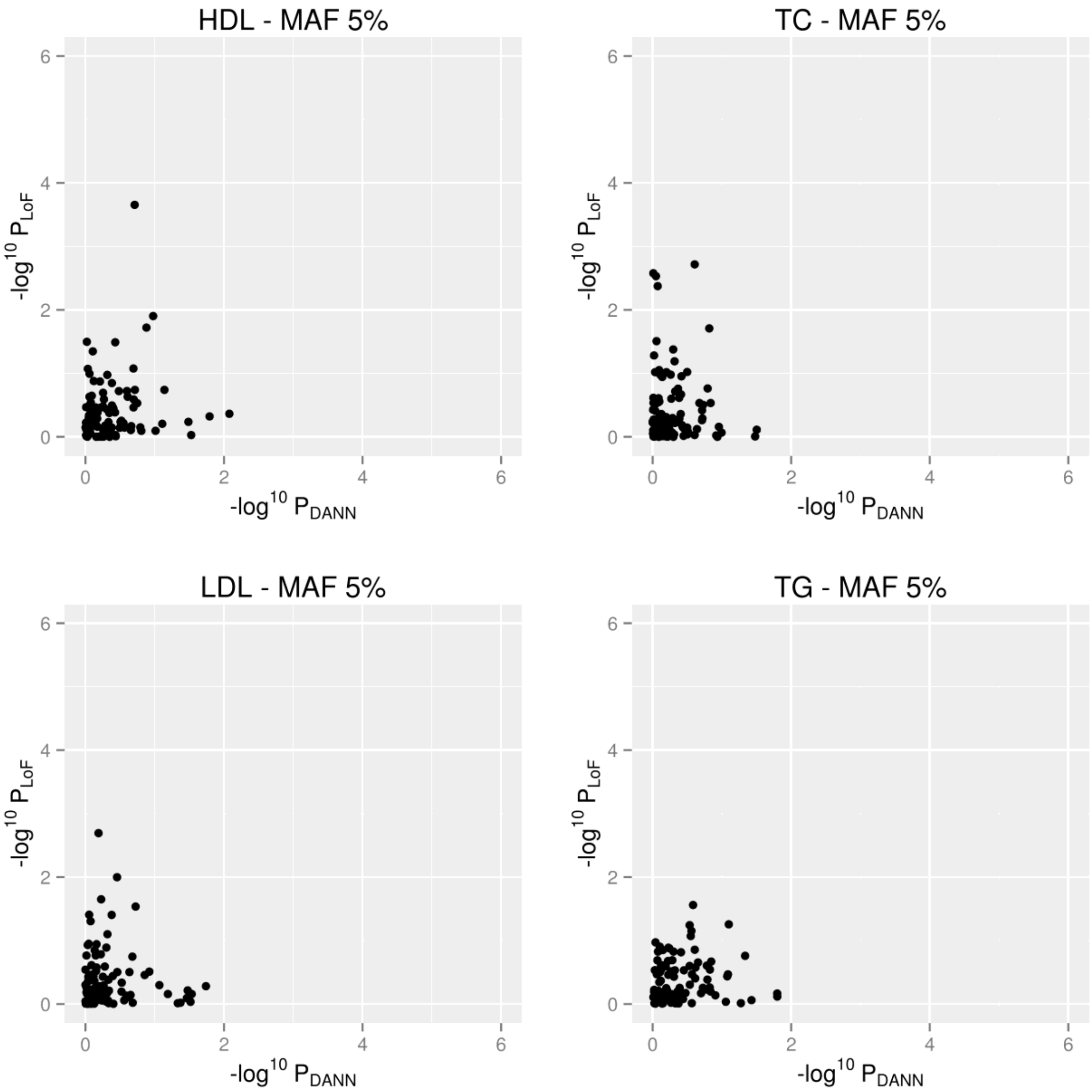

HDL - MAF 5%
TC - MAF 5%
LDL - MAF 5%
TG - MAF 5%
-log10 P_LoF
-log10 P_DANN
0
2
4
6

S28 Figure: Gene-based rare variant analyses across the genome using MiST for 7 cardiovascular traits (compared with loss-of-function filtering)

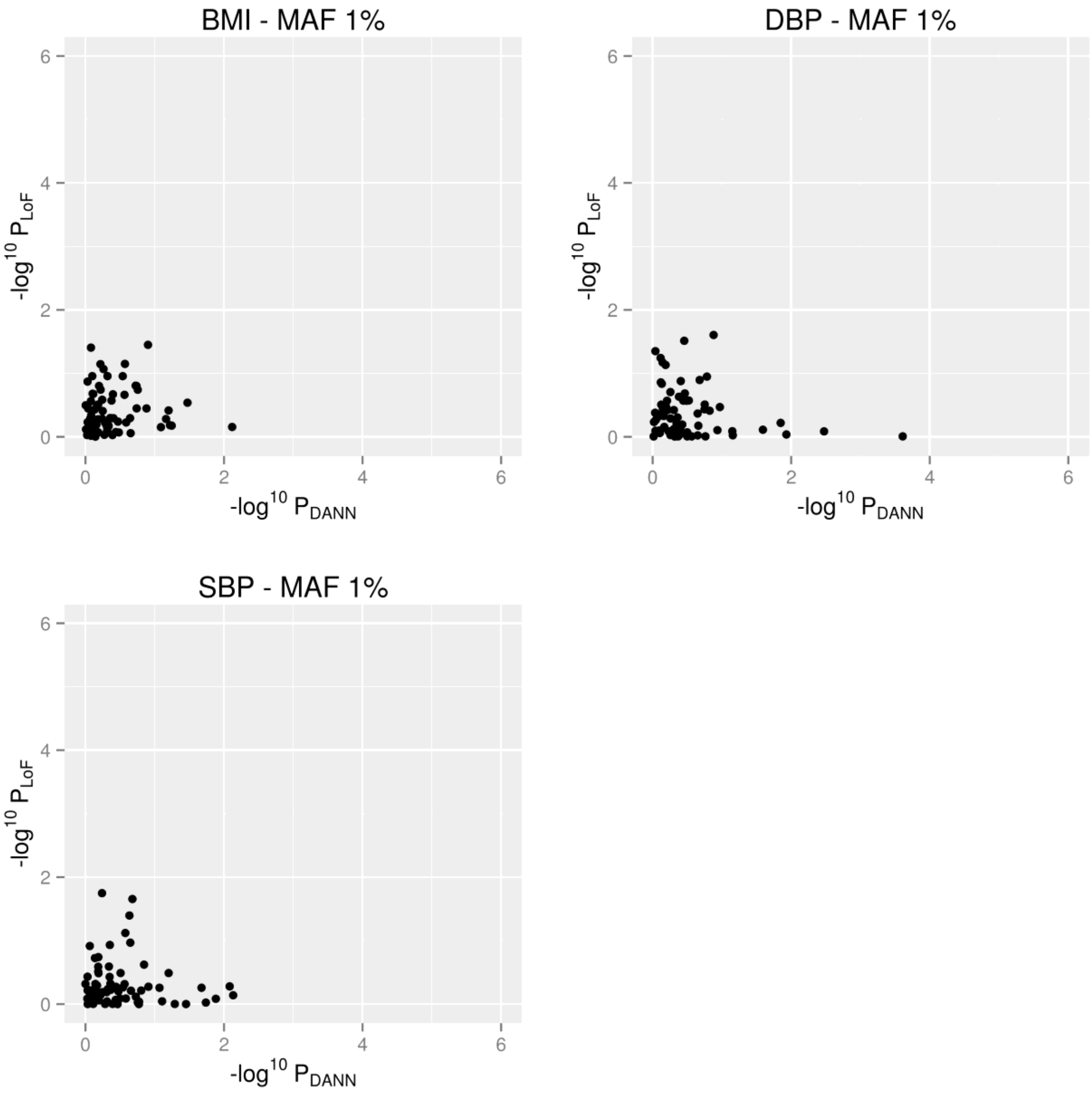

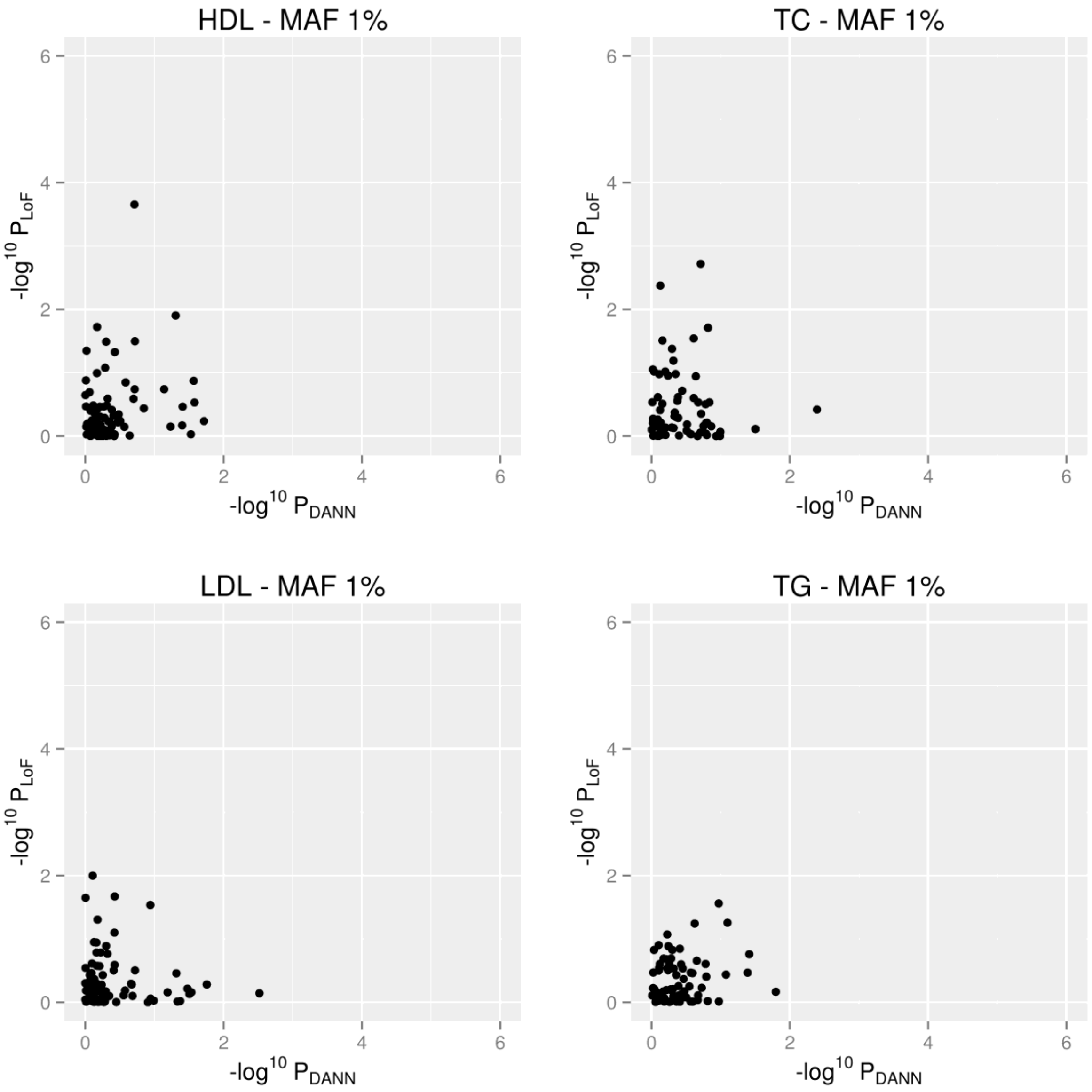

HDL - MAF 1%
TC - MAF 1%
LDL - MAF 1%
TG - MAF 1%
-log10 PLoF
-log10 PDANN
0
2
4
6
